# Supplementary material for: Risk equations for prosthetic joint infections (PJIs) in UK: a retrospective study using the Clinical Practice Research Datalink (CPRD) AURUM and GOLD databases
Source: BMJ Open. 2024 May 7;14(5):e082501. doi: 10.1136/bmjopen-2023-082501 (PMC11086542; doi:10.1136/bmjopen-2023-082501)
Supplement: Supplementary data [file bmjopen-2023-082501supp001.pdf]

# Retrospective study in UK using the Clinical Practice Research Datalink (CPRD) AURUM and GOLD database for the determination of risk equations for prosthetic joint infections (PJI)

*Stefano Perni[1] and Polina Prokopovich[1,]**

School of Pharmacy and Pharmaceutical Sciences, Cardiff University, Cardiff, UK

# Supplementary Material

**Table S 1.** Flow of patients through initial data extraction.

| | **AURUM** | | **GOLD** | |
|---|---|---|---|---|
| | Number of joints | Number of patients | Number of joints | Number of patients |
| Knee or hip replacement in database | 330,173 | 235,249 | 88,458 | 62,672 |
| Over 31 years old at index date | 329,720 | 235,071 | 88,268 | 62,584 |
| Arthroplasty surgery after 2007 | 288,124 | 223,450 | 71,467 | 54,888 |
| With minimum 6 months lookback | 284,048 | 222,060 | 71,467 | 54,888 |
| After removing implausible data | 283,789 | 221,826 | 71,072 | 54,833 |
| Surgery before 2015 | 174,905 | 144,048 | 48,419 | 39,764 |

**Table S 2.** Number of joints not compleating the observation study period (5 years) and reason for loss during follow-up.

| **Reason for not completing study period** | **AURUM** | | **GOLD** | |
|---|---|---|---|---|
| | n | Frequency | n | Frequency |
| Developing PJI | 1,021 | 0.58% | 228 | 0.47% |
| Death | 25,657 | 14.67% | 6,021 | 12.44% |
| Last collection date before end of observation period | 749 | 0.43% | 16,604 | 34.29% |
| Device replaced for any reason | 4,108 | 2.35% | 1,090 | 2.25% |
| Transferred out of CPRD | 22,586 | 12.91% | 4,323 | 8.93% |

**Table S 3.** Follow-up (days) duration in patients not completed study period (5 years).

| | **AURUM** | **GOLD** |
|---|---|---|
| Mean (SD) | 769.61 (578.90) | 798.96 (538.44) |
| Median | 726 | 768 |
| IQR | 197 – 1,281 | 316 – 1,246 |

**Table S 4.** Characteristics of patients at the time of hip or knee implant surgery.

| Variable | All | AURUM | GOLD |
|---|---|---|---|
| **Gender** | | | |
| Female | 137,926 (61.76%) | 107,927 (61.71 %) | 29,999 (61.96 %) |
| Male | 85,398 (38.24%) | 66,979 (38.29 %) | 18,419 (38.04 %) |
| **Age** | | | |
| ≤ 45 | 3,221 (1.44%) | 2,593 (1.48 %) | 628 (1.30 %) |
| 46 – 55 | 13,170 (5.90%) | 10,332 (5.91 %) | 2,838 (5.86 %) |
| 56 – 65 | 43,379 (19.42%) | 34,012 (19.45 %) | 9,367 (19.35 %) |
| 66 – 75 | 75,181 (33.66%) | 58,764 (33.60 %) | 16,417 (33.91 %) |
| 76 – 85 | 65,288 (29.23%) | 51,157 (29.25 %) | 14,131 (29.19 %) |
| > 85 | 23,085 (10.34%) | 18,048 (10.32 %) | 5,037 (10.40 %) |
| Mean (SD) | 71.98 | 71.97 (10.97) | 72.03 (10.88) |
| Median | 73 | 73 | 73 |
| IQR | 65.00 - 80.00 | 65.00 - 80.00 | 65.00 - 80.00 |
| Min, max | 31.00, 109.00 | 31.00, 109.00 | 31.00, 107.00 |
| **BMI** | | | |
| ≤ 20 | 6,364 (2.85%) | 4,111 (2.35 %) | 2,253 (4.65 %) |
| 20 – 25 | 31,896 (14.28%) | 21,173 (12.11 %) | 10,723 (22.15 %) |
| 26 – 30 | 55,729 (24.95%) | 39,077 (22.34 %) | 16,652 (34.39 %) |
| 31 – 35 | 38,395 (17.19%) | 27,950 (15.98 %) | 10,445 (21.57 %) |
| 36 – 50 | 23,629 (10.58%) | 17,849 (10.20 %) | 5,780 (11.94 %) |
| > 50 | 540 (0.24%) | 472 (0.27 %) | 68 (0.14 %) |
| Unknown | 66,771 (29.90%) | 64,274 (36.75 %) | 2,497 (5.16 %) |
| Mean (SD) | 29.22 | 29.52 (6.12) | 28.52 (5.74) |
| Median | 28.6 | 28.9 | 28 |
| IQR | 25.10 - 32.70 | 25.40 - 33.00 | 24.60 - 31.90 |
| Min, max | 10.46, 73.00 | 10.46, 73.00 | 14.50, 67.50 |
| **Smoking status** | | | |
| Non-smoker | 122,373 (54.80%) | 95,096 (54.37 %) | 27,277 (56.34 %) |
| Cigar | 165 (0.07%) | 165 (0.09 %) | 0 (0.00 %) |
| Current | 12,227 (5.48%) | 8,080 (4.62 %) | 4,147 (8.56 %) |
| Ex-smoker | 70,667 (31.64%) | 54,288 (31.04 %) | 16,379 (33.83 %) |
| Heavy | 298 (0.13%) | 230 (0.13 %) | 68 (0.14 %) |
| Light | 772 (0.35%) | 573 (0.33 %) | 199 (0.41 %) |
| Moderate | 496 (0.22%) | 496 (0.28 %) | 0 (0.00 %) |
| Quitting | 674 (0.30%) | 674 (0.39 %) | 0 (0.00 %) |
| Passive smoker | 15 (0.01%) | 0 (0.00 %) | 15 (0.03 %) |
| Unknown | 15,637 (7.00%) | 15,304 (8.75 %) | 333 (0.69 %) |
| **Alcohol consumption** | | | |
| Non-drinker | 8,628 (3.86%) | 3,266 (1.87 %) | 5,362 (11.07 %) |
| Ex drinker | 384 (0.17%) | 270 (0.15 %) | 114 (0.24 %) |
| Heavy drinker | 959 (0.43%) | 899 (0.51 %) | 60 (0.12 %) |

| | | | |
|---|---|---|---|
| Light drinker | 40,588 (18.17%) | 32,762 (18.73 %) | 7,826 (16.16 %) |
| Moderate drinker | 7,284 (3.26%) | 5,416 (3.10 %) | 1,868 (3.86 %) |
| Social drinker | 4,080 (1.83%) | 2,891 (1.65 %) | 1,189 (2.46 %) |
| Very heavy | 690 (0.31%) | 338 (0.19 %) | 352 (0.73 %) |
| Other | 1,546 (0.69%) | 1,080 (0.62 %) | 466 (0.96 %) |
| Unknown | 159,165 (71.27%) | 127,984 (73.17 %) | 31,181 (64.40 %) |

**Table S 5.** Characteristics of hip or knee implant surgery.

| Variable | All | AURUM | GOLD |
|---|---|---|---|
| **Joint replaced** | | | |
| Femur | 31,766 (14.22%) | 24,963 (14.27 %) | 6,803 (14.05 %) |
| Hip | 96,267 (43.11%) | 75,439 (43.13 %) | 20,828 (43.02 %) |
| Knee | 95,291 (42.67%) | 74,504 (42.60 %) | 20,787 (42.93 %) |
| **Laterality of operation** | | | |
| Left | 104,858 (46.95%) | 82,039 (46.90 %) | 22,819 (47.13 %) |
| Right | 118,466 (53.05%) | 92,867 (53.10 %) | 25,599 (52.87 %) |
| **Fixation method** | | | |
| Cemented | 145,726 (65.25%) | 113,248 (64.75 %) | 32,478 (67.08 %) |
| Hybrid | 13,399 (6.00%) | 10,447 (5.97 %) | 2,952 (6.10 %) |
| Non cemented | 53,836 (24.11%) | 43,256 (24.73 %) | 10,580 (21.85 %) |
| Unknown | 10,363 (4.64%) | 7,955 (4.55 %) | 2,408 (4.97 %) |
| **Primary arthroplasty** | | | |
| Yes | 205,321 (91.94%) | 160,883 (91.98 %) | 44,438 (91.78 %) |
| No | 17,226 (7.71%) | 13,404 (7.66 %) | 3,822 (7.89 %) |
| Unknown | 777 (0.35%) | 619 (0.35 %) | 158 (0.33 %) |
| **Admission type** | | | |
| Elective | 184,138 (82.45%) | 144,145 (82.41 %) | 39,993 (82.60 %) |
| A&E | 38,494 (17.24%) | 30,270 (17.31 %) | 8,224 (16.99 %) |
| Unknown | 692 (0.31%) | 491 (0.28 %) | 201 (0.42 %) |
| **Patella resurfacing (* only for knee replacement surgeries)** | | | |
| No | 91,375 (40.92%) | 71,166 (40.69 %) | 20,209 (41.74 %) |
| Yes | 3,916 (1.75%) | 3,338 (1.91 %) | 578 (1.19 %) |
| **Graft** | | | |
| No | 219,645 (98.35%) | 171,999 (98.34 %) | 47,646 (98.41 %) |
| Autograft | 1,673 (0.75%) | 1,331 (0.76 %) | 342 (0.71 %) |
| Autograft + other | 67 (0.03%) | 58 (0.03 %) | 9 (0.02 %) |
| Other graft | 1,939 (0.87%) | 1,518 (0.87 %) | 421 (0.87 %) |
| **Replacement year** | | | |
| 2007 | 24,539 (10.99%) | 18,787 (10.74 %) | 5,752 (11.88 %) |
| 2008 | 26,289 (11.77%) | 20,182 (11.54 %) | 6,107 (12.61 %) |
| 2009 | 26,426 (11.83%) | 20,507 (11.72 %) | 5,919 (12.22 %) |
| 2010 | 28,061 (12.57%) | 21,742 (12.43 %) | 6,319 (13.05 %) |
| 2011 | 28,433 (12.73%) | 22,180 (12.68 %) | 6,253 (12.91 %) |
| 2012 | 29,069 (13.02%) | 22,961 (13.13 %) | 6,108 (12.62 %) |
| 2013 | 29,718 (13.31%) | 23,753 (13.58 %) | 5,965 (12.32 %) |
| 2014 | 30,788 (13.79%) | 24,793 (14.18 %) | 5,995 (12.38 %) |

**Table S 6.** Medical history of patients at the time of hip or knee implant surgery.

| Variable | All | AURUM | GOLD |
|---|---|---|---|
| **Atrial Fibrillation** | | | |
| No | 204,588 (91.61%) | 159,979 (91.47 %) | 44,609 (92.13 %) |
| Yes | 18,736 (8.39%) | 14,927 (8.53 %) | 3,809 (7.87 %) |
| **Liver failure** | | | |
| No | 222,841 (99.78%) | 174,529 (99.78 %) | 48,312 (99.78 %) |
| Yes | 483 (0.22%) | 377 (0.22 %) | 106 (0.22 %) |
| **CKD** | | | |
| No | 193,781 (86.77%) | 146,389 (83.70 %) | 47,392 (97.88 %) |
| Yes | 29,543 (13.23%) | 28,517 (16.30 %) | 1,026 (2.12 %) |
| **Pulmonary Embolism** | | | |
| No | 218,350 (97.77%) | 171,043 (97.79 %) | 47,307 (97.71 %) |
| Yes | 4,974 (2.23%) | 3,863 (2.21 %) | 1,111 (2.29 %) |
| **Deep Vein Thrombosis** | | | |
| No | 213,096 (95.42%) | 167,939 (96.02 %) | 45,157 (93.26 %) |
| Yes | 10,228 (4.58%) | 6,967 (3.98 %) | 3,261 (6.74 %) |
| **Diabetes** | | | |
| No | 191,953 (85.95%) | 151,777 (86.78 %) | 40,176 (82.98 %) |
| Yes | 31,371 (14.05%) | 23,129 (13.22 %) | 8,242 (17.02 %) |
| **Osteoarthritis** | | | |
| No | 72,266 (32.36%) | 55,705 (31.85 %) | 16,561 (34.20 %) |
| Yes | 151,058 (67.64%) | 119,201 (68.15 %) | 31,857 (65.80 %) |
| **Rheumatoid Arthritis** | | | |
| No | 175,330 (78.51%) | 130,011 (74.33 %) | 45,319 (93.60 %) |
| Yes | 47,994 (21.49%) | 44,895 (25.67 %) | 3,099 (6.40 %) |
| **Active Cancer** | | | |
| No | 150,190 (67.25%) | 113,646 (64.98 %) | 36,544 (75.48 %) |
| Yes | 73,134 (32.75%) | 61,260 (35.02 %) | 11,874 (24.52 %) |
| **Heart Failure** | | | |
| No | 218,251 (97.73%) | 170,804 (97.65 %) | 47,447 (97.99 %) |
| Yes | 5,073 (2.27%) | 4,102 (2.35 %) | 971 (2.01 %) |
| **Myocardial Infarction** | | | |
| No | 202,834 (90.82%) | 156,757 (89.62 %) | 46,077 (95.17 %) |
| Yes | 20,490 (9.18%) | 18,149 (10.38 %) | 2,341 (4.83 %) |
| **Ischaemic Heart Disease** | | | |
| No | 198,515 (88.89%) | 154,504 (88.34 %) | 44,011 (90.90 %) |
| Yes | 24,809 (11.11%) | 20,402 (11.66 %) | 4,407 (9.10 %) |
| **Hypertension** | | | |
| No | 99,688 (44.64%) | 79,303 (45.34 %) | 20,385 (42.10 %) |
| Yes | 123,636 (55.36%) | 95,603 (54.66 %) | 28,033 (57.90 %) |
| **COPD** | | | |
| No | 207,899 (93.09%) | 162,676 (93.01 %) | 45,223 (93.40 %) |

| | | | |
|---|---|---|---|
| Yes | 15,425 (6.91%) | 12,230 (6.99 %) | 3,195 (6.60 %) |
| **Haemorrhagic stroke** | | | |
| No | 219,436 (98.26%) | 171,293 (97.93 %) | 48,143 (99.43 %) |
| Yes | 3,888 (1.74%) | 3,613 (2.07 %) | 275 (0.57 %) |
| **Ischemic stroke** | | | |
| No | 215,807 (96.63%) | 170,417 (97.43 %) | 45,390 (93.75 %) |
| Yes | 7,517 (3.37%) | 4,489 (2.57 %) | 3,028 (6.25 %) |
| **Dementia** | | | |
| No | 216,525 (96.96%) | 169,637 (96.99 %) | 46,888 (96.84 %) |
| Yes | 6,799 (3.04%) | 5,269 (3.01 %) | 1,530 (3.16 %) |
| **Thyroidism** | | | |
| No | 215,894 (96.67%) | 173,581 (99.24 %) | 42,313 (87.39 %) |
| Yes | 7,430 (3.33%) | 1,325 (0.76 %) | 6,105 (12.61 %) |
| **Anaemia** | | | |
| No | 199,438 (89.30%) | 151,549 (86.65 %) | 47,889 (98.91 %) |
| Yes | 23,886 (10.70%) | 23,357 (13.35 %) | 529 (1.09 %) |
| **Osteoporosis** | | | |
| No | 205,345 (91.95%) | 161,061 (92.08 %) | 44,284 (91.46 %) |
| Yes | 17,979 (8.05%) | 13,845 (7.92 %) | 4,134 (8.54 %) |
| **PJI before in same joint** | | | |
| No | 222,353 (99.57%) | 174,100 (99.54 %) | 48,253 (99.66 %) |
| Yes | 971 (0.43%) | 806 (0.46 %) | 165 (0.34 %) |
| **PJI before in another joint (* not only hip or knee)** | | | |
| No | 220,838 (98.89%) | 173,074 (98.95 %) | 47,764 (98.65 %) |
| Yes | 2,486 (1.11%) | 1,832 (1.05 %) | 654 (1.35 %) |

**Table S 7.** Treatment history of patients at the time of hip or knee implant surgery.

| Variable | All | AURUM | GOLD |
|---|---|---|---|
| **Steroids injection** | | | |
| No use | 206,245 (92.35%) | 164,047 (93.79 %) | 42,198 (87.15 %) |
| Use < 3 months | 1,256 (0.56%) | 810 (0.46 %) | 446 (0.92 %) |
| Use 3 - 6 months | 2,276 (1.02%) | 1,456 (0.83 %) | 820 (1.69 %) |
| Use > 6 months | 13,547 (6.07%) | 8,593 (4.91 %) | 4,954 (10.23 %) |
| **Chondroitin glucosamine** | | | |
| No use | 219,306 (98.20%) | 173,018 (98.92 %) | 46,288 (95.60 %) |
| Use < 3 months | 930 (0.42%) | 482 (0.28 %) | 448 (0.93 %) |
| Use 3 - 6 months | 259 (0.12%) | 134 (0.08 %) | 125 (0.26 %) |
| Use > 6 months | 2,829 (1.27%) | 1,272 (0.73 %) | 1,557 (3.22 %) |
| **NSAID** | | | |
| No use | 97,243 (43.54%) | 89,492 (51.17 %) | 7,751 (16.01 %) |
| Use < 3 months | 48,354 (21.65%) | 37,077 (21.20 %) | 11,277 (23.29 %) |
| Use 3 - 6 months | 13,169 (5.90%) | 9,851 (5.63 %) | 3,318 (6.85 %) |
| Use > 6 months | 64,558 (28.91%) | 38,486 (22.00 %) | 26,072 (53.85 %) |
| **Methotrexate** | | | |
| No use | 218,503 (97.84%) | 171,414 (98.00 %) | 47,089 (97.26 %) |
| Use < 3 months | 3,255 (1.46%) | 2,559 (1.46 %) | 696 (1.44 %) |
| Use 3 - 6 months | 174 (0.08%) | 124 (0.07 %) | 50 (0.10 %) |
| Use > 6 months | 1,392 (0.62%) | 809 (0.46 %) | 583 (1.20 %) |
| **DMRAD** | | | |
| No use | 219,948 (98.49%) | 172,997 (98.91 %) | 46,951 (96.97 %) |
| Use < 3 months | 1,626 (0.73%) | 1,188 (0.68 %) | 438 (0.90 %) |
| Use 3 - 6 months | 155 (0.07%) | 112 (0.06 %) | 43 (0.09 %) |
| Use > 6 months | 1,595 (0.71%) | 609 (0.35 %) | 986 (2.04 %) |
| **Antibacterial** | | | |
| No use | 91,794 (41.10%) | 68,716 (39.29 %) | 23,078 (47.66 %) |
| Use < 3 months | 35,730 (16.00%) | 34,370 (19.65 %) | 1,360 (2.81 %) |
| Use 3 - 6 months | 15,961 (7.15%) | 14,903 (8.52 %) | 1,058 (2.19 %) |
| Use > 6 months | 79,839 (35.75%) | 56,917 (32.54 %) | 22,922 (47.34 %) |
| **Antifungal** | | | |
| No use | 192,800 (86.33%) | 148,117 (84.68 %) | 44,683 (92.29 %) |
| Use < 3 months | 4,900 (2.19%) | 4,747 (2.71 %) | 153 (0.32 %) |
| Use 3 - 6 months | 2,842 (1.27%) | 2,721 (1.56 %) | 121 (0.25 %) |
| Use > 6 months | 22,782 (10.20%) | 19,321 (11.05 %) | 3,461 (7.15 %) |
| **Direct Oral Anticoagulant** | | | |
| No use | 222,761 (99.75%) | 174,417 (99.72 %) | 48,344 (99.85 %) |
| Use < 3 months | 324 (0.15%) | 288 (0.16 %) | 36 (0.07 %) |
| Use 3 - 6 months | 54 (0.02%) | 51 (0.03 %) | 3 (0.01 %) |
| Use > 6 months | 185 (0.08%) | 150 (0.09 %) | 35 (0.07 %) |
| **Heparin** | | | |
| No use | 221,208 (99.05%) | 173,345 (99.11 %) | 47,863 (98.85 %) |

| | | | |
|---|---|---|---|
| Use < 3 months | 334 (0.15%) | 317 (0.18 %) | 17 (0.04 %) |
| Use 3 - 6 months | 163 (0.07%) | 142 (0.08 %) | 21 (0.04 %) |
| Use > 6 months | 1,619 (0.72%) | 1,102 (0.63 %) | 517 (1.07 %) |
| **Warfarin** | | | |
| No use | 208,608 (93.41%) | 164,436 (94.01 %) | 44,172 (91.23 %) |
| Use < 3 months | 9,494 (4.25%) | 7,499 (4.29 %) | 1,995 (4.12 %) |
| Use 3 - 6 months | 879 (0.39%) | 695 (0.40 %) | 184 (0.38 %) |
| Use > 6 months | 4,343 (1.94%) | 2,276 (1.30 %) | 2,067 (4.27 %) |
| **Intra-articular injections (n)** | | | |
| 0 | 215,281 (96.40%) | 168,424 (96.29 %) | 46,857 (96.78 %) |
| 1 | 5,856 (2.62%) | 4,709 (2.69 %) | 1,147 (2.37 %) |
| 2 | 1,320 (0.59%) | 1,063 (0.61 %) | 257 (0.53 %) |
| 3 | 444 (0.20%) | 367 (0.21 %) | 77 (0.16 %) |
| 4 | 164 (0.07%) | 130 (0.07 %) | 34 (0.07 %) |
| 5 | 117 (0.05%) | 96 (0.05 %) | 21 (0.04 %) |
| > 5 | 142 (0.06%) | 117 (0.07 %) | 25 (0.05 %) |
| **Last intra-articular injection** | | | |
| No use | 215,281 (96.40%) | 168,424 (96.29 %) | 46,857 (96.78 %) |
| Use < 3 months | 341 (0.15%) | 279 (0.16 %) | 62 (0.13 %) |
| Use 3 - 6 months | 1,309 (0.59%) | 1,036 (0.59 %) | 273 (0.56 %) |
| Use > 6 months | 6,393 (2.86%) | 5,167 (2.95 %) | 1,226 (2.53 %) |
| **Intra-articular injection in other joints (n)** | | | |
| 0 | 200,901 (89.96%) | 156,820 (89.66 %) | 44,081 (91.04 %) |
| 1 | 258 (0.12%) | 200 (0.11 %) | 58 (0.12 %) |
| 2 | 3,014 (1.35%) | 2,401 (1.37 %) | 613 (1.27 %) |
| 3 | 6,579 (2.95%) | 5,270 (3.01 %) | 1,309 (2.70 %) |
| 4 | 4,315 (1.93%) | 3,438 (1.97 %) | 877 (1.81 %) |
| 5 | 1,407 (0.63%) | 1,130 (0.65 %) | 277 (0.57 %) |
| > 5 | 6,850 (3.07%) | 5,647 (3.23 %) | 1,203 (2.48 %) |
| **Last intra-articular injection in other joints** | | | |
| No use | 200,901 (89.96%) | 156,820 (89.66 %) | 44,081 (91.04 %) |
| Use < 3 months | 974 (0.44%) | 777 (0.44 %) | 197 (0.41 %) |
| Use 3 - 6 months | 2,885 (1.29%) | 2,305 (1.32 %) | 580 (1.20 %) |
| Use > 6 months | 18,564 (8.31%) | 15,004 (8.58 %) | 3,560 (7.35 %) |

**Table S 8.** Summary of fitting performance measuremement for fitting results from CPRD AURUM database with different parametric models.

| Parametric model | AIC | BIC | Loglik |
|---|---|---|---|
| Exponential | 26671.5 | 27749.2 | -13730.0 |
| Gaussian | 28030.7 | 29118.5 | -15416.5 |
| Logistic | 28097.6 | 29185.4 | -14439.8 |
| Log-logistic | 26190.8 | 27278.6 | -13479.9 |
| Lognormal | 26187.4 | 27275.2 | -13459.8 |
| Weibull | 26192.6 | 27280.4 | -13480.2 |

**Table S 9**. Summary of fitting performance measuremement for fitting results from CPRD GOLD database with different parametric models.

| Parametric model | AIC | BIC | Loglik |
|---|---|---|---|
| Exponential | 7674.76 | 8573.56 | -3927.22 |
| Gaussian | 8097.86 | 9005.83 | -4134.94 |
| Logistic | 8116.71 | 9024.69 | -4146.93 |
| Log-logistic | 7543.99 | 8451.96 | -3857.65 |
| Lognormal | 7463.78 | 7793.95 | -3852.10 |
| Weibull | 7544.66 | 8452.64 | -3857.71 |

**Table S 10.** Regression coefficients and standard error (SE) for log-normal risk equation of time to PJI derived from CPRD AURUM and CPRD GOLD.

| **Variable** | **AURUM** | **GOLD** | **SE AURUM** | **SE GOLD** | ***P* value** |
|---|---|---|---|---|---|
| **Scale** | 5.194 | 4.759 | 1.404 | 1.650 | 0.841 |
| **Intercept** | 19.470 | 20.618 | 0.132 | 0.264 | < 0.001 |
| **Male** | -0.813 | -0.952 | 0.408 | 0.967 | 0.895 |
| **Age** | | | | | |
| ≤ 45 | Reference | | | | |
| 46 – 55 | 1.247 | 0.134 | 0.378 | 0.931 | 0.268 |
| 56 – 65 | 1.611 | 0.843 | 0.379 | 0.939 | 0.448 |
| 66 – 75 | 1.968 | 1.241 | 0.389 | 0.958 | 0.482 |
| 76 – 85 | 2.044 | 1.299 | 0.498 | 1.274 | 0.586 |
| > 85 | 3.390 | 3.329 | 0.550 | 0.988 | 0.957 |
| **BMI** | | | | | |
| ≤ 20 | Reference | | | | |
| 20 - 25 | -0.271 | -0.946 | 0.535 | 0.983 | 0.546 |
| 25 - 30 | -0.347 | -0.493 | 0.54 | 0.992 | 0.897 |
| 30 - 35 | -0.715 | -0.934 | 0.547 | 1.012 | 0.849 |
| 35 - 50 | -1.186 | -1.271 | 0.979 | 2.093 | 0.971 |
| >50 | -1.628 | -2.693 | 0.526 | 1.132 | 0.394 |
| Unknown | -1.116 | -0.653 | 1.955 | 0.432 | 0.817 |
| **Smoking status** | | | | | |
| Non-smoker | Reference | | | | |
| Cigar smoker | 0.249 | NA | 0.284 | NA | NA |
| Current smoker | -0.181 | -0.091 | 0.137 | 0.265 | 0.763 |
| Ex-smoker | -0.194 | 0.157 | 1.359 | 2.274 | 0.895 |
| Heavy smoker | -0.623 | -1.003 | 1.112 | 1.361 | 0.829 |
| Light smoker | 0.609 | -1.455 | 1.140 | 0.001 | 0.070 |
| Moderate smoker | 0.127 | NA | 1.004 | NA | NA |
| Quitting | 0.469 | NA | 0.22 | NA | NA |
| Unknown | -0.083 | 0.074 | 1.391 | 1.854 | 0.946 |
| Passive smoker | NA | NA | NA | 1.483 | NA |
| **Alcohol consumption** | | | | | |
| Non-drinker | Reference | | | | |
| Ex drinker | 0.866 | NA | 1.199 | 0.465 | NA |
| Heavy drinker | 0.828 | -0.218 | 1.231 | 0.759 | 0.47 |
| Light drinker | 0.966 | 0.761 | 1.302 | 0.973 | 0.901 |
| Moderate drinker | 1.706 | NA | 1.36 | NA | NA |
| Other | 0.825 | 0.805 | 1.267 | 0.405 | 0.988 |
| Social drinker | 0.783 | 0.962 | 1.194 | 0.002 | 0.881 |
| Unknown | 1.098 | -0.118 | 1.547 | 0.241 | 0.437 |
| Very heavy drinker | 0.091 | NA | 0.124 | 1.334 | NA |
| **Right side of operation** | 0.060 | 0.474 | 0.285 | 0.544 | 0.504 |
| **Type of fixation** | | | | | |

| | | | | | |
|---|---|---|---|---|---|
| Cemented | Reference | | | | |
| Hybrid | 0.155 | -0.801 | 0.164 | 0.369 | 0.018 |
| Non cemented | 0.123 | 0.106 | 0.269 | 0.51 | 0.976 |
| Unknown | 0.048 | -0.024 | 0.181 | 0.365 | 0.860 |
| **Primary arthroplasty** | | | | | |
| Yes | Reference | | | | |
| No | -2.655 | -1.927 | 0.656 | 1.953 | 0.724 |
| Unknown | -2.674 | -0.622 | 0.211 | 0.451 | < 0.001 |
| **Admission type** | | | | | |
| Elective | Reference | | | | |
| A&E | -1.013 | -1.256 | 1.157 | 1.767 | 0.908 |
| Unknown | 0.618 | -0.630 | 0.490 | 0.763 | 0.169 |
| **Patella resurfacing** | | | | | |
| No | NA | NA | NA | NA | NA |
| Yes | 0.736 | -0.862 | 0.151 | 0.326 | < 0.001 |
| N/A | 0.437 | 1.463 | 0.635 | 81880.48 | 1 |
| **Graft** | | | | | |
| No | Reference | | | | |
| Autograft | -0.048 | 26.046 | 2.238 | 2.630 | < 0.001 |
| Autograft + other | -0.458 | -6.485 | 0.486 | 1.256 | < 0.001 |
| Other graft | 0.152 | 0.597 | 0.263 | 0.525 | 0.449 |
| **Replacement year** | | | | | |
| 2007 | Reference | | | | |
| 2008 | 0.387 | 0.428 | 0.283 | 0.482 | 0.942 |
| 2009 | 0.506 | 0.031 | 0.284 | 0.467 | 0.385 |
| 2010 | 0.378 | -0.238 | 0.288 | 0.489 | 0.278 |
| 2011 | 0.35 | 0.065 | 0.294 | 0.493 | 0.62 |
| 2012 | 0.521 | -0.062 | 0.302 | 0.472 | 0.298 |
| 2013 | 0.637 | -0.674 | 0.305 | 0.487 | 0.023 |
| 2014 | 0.635 | -0.383 | 0.028 | 0.058 | < 0.001 |
| **Atrial Fibrillation** | -0.259 | -0.784 | 0.182 | 1.128 | 0.646 |
| **CKD** | -0.094 | 0.813 | 1.227 | 0 | 0.46 |
| **Liver failure** | 0.299 | NA | 0.394 | 0.751 | NA |
| **Pulmonary Embolism** | -0.071 | -0.517 | 0.276 | 0.511 | 0.443 |
| **Deep Vein Thrombosis** | -0.531 | 0.362 | 0.168 | 0.321 | 0.014 |
| **Diabetes** | -0.468 | 0.205 | 0.145 | 0.272 | 0.029 |
| **Osteoarthritis** | -0.249 | -0.199 | 0.143 | 0.506 | 0.924 |
| **Rheumatoid Arthritis** | -0.005 | -0.274 | 0.136 | 0.287 | 0.397 |
| **Active Cancer** | 0.005 | -0.159 | 0.425 | 1.034 | 0.883 |
| **Heart Failure** | 0.071 | 0.651 | 0.322 | 0.691 | 0.447 |
| **Myocardial Infarction** | -0.616 | 0.717 | 0.312 | 0.464 | 0.017 |
| **Ischaemic Heart Disease** | 0.286 | -0.130 | 0.130 | 0.255 | 0.146 |

| | | | | | |
|---|---|---|---|---|---|
| **Hypertension** | -0.127 | -0.254 | 0.256 | 0.496 | 0.820 |
| **COPD** | 0.298 | -0.213 | 0.639 | 1.655 | 0.773 |
| **Haemorrhagic Stroke** | 0.616 | -0.263 | 0.549 | 0.539 | 0.253 |
| **Ischaemic Stroke** | 0.094 | 0.022 | 0.563 | 0.975 | 0.949 |
| **Dementia** | 0.473 | -0.466 | 0.701 | 0.369 | 0.235 |
| **Thyroidism** | -0.309 | -0.197 | 0.182 | 0 | 0.538 |
| **Anaemia** | -0.026 | NA | 0.260 | 0.557 | NA |
| **Osteoporosis** | 0.135 | 0.648 | 0.397 | 0.895 | 0.600 |
| **PJI before in same joint** | -2.493 | -0.872 | 0.333 | 0.596 | 0.018 |
| **PJI before in another joint** | -1.726 | -2.178 | 0.766 | 1.204 | 0.751 |
| **Steroids injection** | | | | | |
| No use | Reference | | | | |
| Use ≤ 3 months | -0.629 | 0.165 | 0.543 | 0.979 | 0.478 |
| Use 3 - 6 months | -0.962 | 0.777 | 0.258 | 0.364 | < 0.001 |
| Use > 6 months | -0.383 | -0.079 | 1.368 | 1.607 | 0.885 |
| **Chondroitin Glucosamine** | | | | | |
| No use | Reference | | | | |
| Use ≤ 3 months | 0.920 | 1.314 | 1.841 | 0 | 0.831 |
| Use 3 - 6 months | -0.791 | NA | 0.638 | 0.692 | NA |
| Use > 6 months | -0.334 | 0.622 | 0.162 | 0.474 | 0.056 |
| **NSAID** | | | | | |
| No use | Reference | | | | |
| Use ≤ 3 months | -0.678 | -1.552 | 0.257 | 0.568 | 0.161 |
| Use 3 - 6 months | -0.472 | -1.482 | 0.177 | 0.448 | 0.036 |
| Use > 6 months | 0.136 | -0.459 | 0.440 | 0.980 | 0.580 |
| **Methotrexate** | | | | | |
| No use | Reference | | | | |
| Use ≤ 3 months | -0.787 | 0.358 | 0 | 0 | < 0.001 |
| Use 3 - 6 months | NA | NA | 0.771 | 0.936 | NA |
| Use > 6 months | -0.668 | -0.329 | 0.897 | 0.87 | 0.786 |
| **DMRAD** | | | | | |
| No use | Reference | | | | |
| Use ≤ 3 months | 1.494 | -2.089 | 0 | 0 | < 0.001 |
| Use 3 - 6 months | NA | NA | 0.875 | 0.826 | NA |
| Use > 6 months | -0.434 | 0.023 | 0.19 | 0.767 | 0.563 |
| **Antibacterial** | | | | | |
| No use | Reference | | | | |
| Use ≤ 3 months | -0.394 | 0.376 | 0.233 | 0.686 | 0.288 |
| Use 3 - 6 months | -0.568 | -0.889 | 0.174 | 0.249 | 0.291 |
| Use > 6 months | -0.362 | -0.136 | 0.324 | 0 | 0.485 |
| **Antifungal** | | | | | |
| No use | Reference | | | | |
| Use ≤ 3 months | -0.728 | NA | 0.467 | 0 | NA |

| | | | | | |
|---|---|---|---|---|---|
| Use 3 - 6 months | -0.115 | NA | 0.186 | 0.364 | NA |
| Use > 6 months | -0.434 | -1.086 | 1.112 | 2.132 | 0.786 |
| **Direct Oral Anticoagulant** | | | | | |
| No use | Reference | | | | |
| Use ≤ 3 months | -1.508 | -3.789 | 0 | 0 | < 0.001 |
| Use 3 - 6 months | NA | NA | 2.132 | 0 | NA |
| Use > 6 months | 0.694 | NA | 1.081 | 0 | NA |
| **Heparin** | | | | | |
| No use | Reference | | | | |
| Use ≤ 3 months | -0.878 | NA | 1.239 | 0 | NA |
| Use 3 - 6 months | -2.067 | NA | 0.732 | 1.011 | NA |
| Use > 6 months | 0.162 | -0.484 | 0.350 | 0.661 | 0.388 |
| **Warfarin** | | | | | |
| No use | Reference | | | | |
| Use ≤ 3 months | -0.021 | -0.914 | 0.832 | 1.476 | 0.598 |
| Use 3 - 6 months | -0.433 | -1.07 | 0.531 | 0.640 | 0.444 |
| Use > 6 months | 0.273 | -0.053 | 1.714 | 3527.096 | 1 |
| **Intra-articular injection** | | | | | |
| No use | Reference | | | | |
| Use ≤ 3 months | 0.308 | 19.912 | 1.647 | 0 | < 0.001 |
| Use 3 - 6 months | -4.414 | NA | 0.396 | 0.915 | NA |
| Use > 6 months | -0.171 | -0.238 | 0.973 | 1.973 | 0.976 |
| **Intra-articular injections in other joints** | | | | | |
| No use | Reference | | | | |
| Use ≤ 3 months | -0.358 | -0.131 | 1.519 | 1.222 | 0.907 |
| Use 3 - 6 months | 3.417 | -0.589 | 0.239 | 0.529 | < 0.001 |
| Use > 6 months | -0.144 | 0.349 | 0.270 | 0.498 | 0.384 |

**Table S 11.** C- statistic values for pooled risk equation at different follow-up.

| Follow-up | Mean | 95% CI |
|---|---|---|
| 1 year | 0.733 | 0.712 - 0.756 |
| 2 years | 0.727 | 0.708 - 0.744 |
| 3 years | 0.725 | 0.708 - 0.743 |
| 4 years | 0.723 | 0.708 - 0.738 |
| 5 years | 0.721 | 0.706 - 0.736 |
