# Supplementary material for: Risk equations for prosthetic joint infections (PJIs) in UK: a retrospective study using the Clinical Practice Research Datalink (CPRD) AURUM and GOLD databases
Source: BMJ Open. 2024 May 7;14(5):e082501. doi: 10.1136/bmjopen-2023-082501 (PMC11086542; doi:10.1136/bmjopen-2023-082501)
Supplement: Author's manuscript [file bmjopen-2023-082501.draft_revisions.pdf]

BMJ Open is committed to open peer review. As part of this commitment we make the peer review history of every article we publish publicly available.

When an article is published we post the peer reviewers' comments and the authors' responses online. We also post the versions of the paper that were used during peer review. These are the versions that the peer review comments apply to.

The versions of the paper that follow are the versions that were submitted during the peer review process. They are not the versions of record or the final published versions. They should not be cited or distributed as the published version of this manuscript.

BMJ Open is an open access journal and the full, final, typeset and author-corrected version of record of the manuscript is available on our site with no access controls, subscription charges or pay-per-view fees (http://bmjopen.bmj.com).

If you have any questions on BMJ Open's open peer review process please email info.bmjopen@bmj.com

BMJ Open

# Risk equations for prosthetic joint infections (PJI) using the Clinical Practice Research Datalink (CPRD) AURUM and GOLD database


| | |
|---|---|
| Journal: | *BMJ Open* |
| Manuscript ID | bmjopen-2023-082501 |
| Article Type: | Original research |
| Date Submitted by the Author: | 28-Nov-2023 |



| | |
|---|---|
| Complete List of Authors: | Perni, Stefano; Cardiff University<br>Prokopovich, Polina; Cardiff University |



| | |
|---|---|
| Keywords: | Epidemiology < INFECTIOUS DISEASES, Knee < ORTHOPAEDIC & TRAUMA SURGERY, Hip < ORTHOPAEDIC & TRAUMA SURGERY, Orthopaedic & trauma surgery < SURGERY, Electronic Health Records |


SCHOLARONE™
Manuscripts

*I, the Submitting Author has the right to grant and does grant on behalf of all authors of the Work (as defined in the below author licence), an exclusive licence and/or a non-exclusive licence for contributions from authors who are: i) UK Crown employees; ii) where BMJ has agreed a CC-BY licence shall apply, and/or iii) in accordance with the terms applicable for US Federal Government officers or employees acting as part of their official duties; on a worldwide, perpetual, irrevocable, royalty-free basis to BMJ Publishing Group Ltd ("BMJ") its licensees and where the relevant Journal is co-owned by BMJ to the co-owners of the Journal, to publish the Work in this journal and any other BMJ products and to exploit all rights, as set out in our licence.*

*The Submitting Author accepts and understands that any supply made under these terms is made by BMJ to the Submitting Author unless you are acting as an employee on behalf of your employer or a postgraduate student of an affiliated institution which is paying any applicable article publishing charge ("APC") for Open Access articles. Where the Submitting Author wishes to make the Work available on an Open Access basis (and intends to pay the relevant APC), the terms of reuse of such Open Access shall be governed by a Creative Commons licence – details of these licences and which Creative Commons licence will apply to this Work are set out in our licence referred to above.*

*Other than as permitted in any relevant BMJ Author's Self Archiving Policies, I confirm this Work has not been accepted for publication elsewhere, is not being considered for publication elsewhere and does not duplicate material already published. I confirm all authors consent to publication of this Work and authorise the granting of this licence.*

For peer review only

# Risk equations for prosthetic joint infections (PJI) using the Clinical Practice Research Datalink (CPRD) AURUM and GOLD database

*Stefano Perni[1] and Polina Prokopovich[1,]**

[1] School of Pharmacy and Pharmaceutical Sciences, Cardiff University, Cardiff, UK

WORD COUNT: 3138

**Corresponding author: Dr Polina Prokopovich**

School of Pharmacy and Pharmaceutical Sciences

Cardiff University

Redwood Building, King Edward VII Avenue

Cardiff, UK

CF10 3NB

E-mail address: prokopovichp@cf.ac.uk



For peer review only - http://bmjopen.bmj.com/site/about/guidelines.xhtml

# 1 Abstract

**Background:** Prosthetic joint infections (PJI) are a serious negative outcome of arthroplasty with incidence of about 1%. Risk of PJI could depend on local treatment policies and guidelines; no UK specific risk scoring is currently available.

**Objective:** to use electronic health records to determine a risk quantification model for the development of PJI

**Design:** Records in Clinical practice research datalink (CPRD) GOLD and AURUM of patients undergoing hip or knee arthroplasty between Jan 2007 and July 2020, with linkage to Hospital episode statistic (HES) and office of national statistic (ONS), were obtained. Cohorts' characteristics and risk equations through parametric models were developed and compared between the two databases. Pooled cohort risk equations were determined for the UK population and simplified through stepwise selection.

**Results:** After applying the inclusion/exclusion criteria 283,789 joint (1,402 developed PJI) were identified in CPRD AURUM and 71,071 joint (288 developed PJI) in CPRD GOLD. Patients undergoing hip or knee arthroplasty in both databases exhibited different sociodemographic characteristics and medical/drug history. However, the quantification of the impact of such covariate (coefficients of parametric models fitted to the survival curves) in the risk of PJI are not statistically different between the two cohorts. The log-normal model fitted to the pooled cohorts after stepwise selection had a C-statistic > 0.7.

**Conclusions:** the risk prediction tool developed here could help preventing PJI through identifying modifiable risk factors pre-surgery and identify the patients most likely to benefit from close monitoring/preventive actions. As derive from the UK population, such tool will help the NHS in reducing the impact of PJI on its resources and patient lives.


**Keywords:** PJI, CPRD, pooled cohort equations, risk prediction



For peer review only - http://bmjopen.bmj.com/site/about/guidelines.xhtml

## 2 List of abbreviations

| | |
|---|---|
| AC | Active cancer |
| A&E | Accident and Emergency |
| AF | Atrial Fibrillation |
| AIC | Akaike Information Criterion |
| BIC | Bayesian Information Criterion |
| BMI | Body mass index |
| CKD | Chronic kidney disease |
| COPD | Chronic obstructive pulmonary disease |
| DAIR | Debridement, antibiotic implant retention |
| DMRAD | Disease modifying rheumatoid arthritis drug |
| DOAC | Direct oral anticoagulant |
| DVT | Deep vein thrombosis |
| HES | Hospital episode statistic |
| HF | Heart failure |
| IHD | Ischaemic heart disease |
| IQR | Interquartile range |
| LoS | Length of Stay |
| MI | Myocardial infarction |
| MTX | Methotrexate |
| NHS | National health service |
| NSAID | Non-steroidal anti-inflammatory drug |
| PE | Pulmonary embolism |
| PJI | Prostatic joint infection |
| OA | Osteoarthritis |
| ONS | Office national statistic |
| RA | Rheumatoid arthritis |

For peer review only

# 3 Strengths and Limitations

- We sampled a large cohort of patients undergoing hip or knee replacement and considered a wide-range of covariates and of drugs to establish the medical and medication history.
- , It is plausible some confounding variables are unavailable, either because they are not routinely recorded (for example, nutritional status and genetic factors) or unavailable (for example, hospital prescribing and use of over-the-counter medications).
- The use of registry-based data incurs in the possibility of error in the recording of data or codes may be a source of information bias.
- The specific geographical location (UK) may limit the availability of data regarding the impact of ethnicity and not have the same predictive power when applied to other countries.
- As the CPRD is a retrospective observational database, clinical measurements will be collected through routine primary care rather than being mandated through a study protocol, as in a prospective study leading to missing data.
- Study population is restricted to patients with complete data at baseline for certain variables. This may invite bias, as the patient profile of those with complete data may be different to those without. However, a workable dataset is required to fulfil the study objectives and, therefore, this practical approach will be adopted to identify a suitable study population.



For peer review only - http://bmjopen.bmj.com/site/about/guidelines.xhtml

# 4 Introduction

Infections associated to arthroplasty, known as prosthetic joint infections (PJIs), have and incidence of about 1% after primary arthroplasty and about 5 times higher after secondary arthroplasty resulting from a previous PJI.[1 2] The consequences of PJI can be extremely debilitating long-term for patients [3] and, with an estimate mean cost to the NHS of ~£30,000 per PJI in knee joints [4] and ~£24,000 for PJI in hips, also health care providers incur high costs.

Numerous risk factors for PJI have identified and risk quantification tools developed [5-9] as the possibility of identifying patients at risks for PJI would allow a more proactive targeted approach to prevention; however, the quantification of the individual probability to develop PJI is often impacted by the size of the cohort used in establishing such risk equations and the covariates available in the datasets employed for such analysis.[10 11] Such limitations reduce the ability to translate conclusions to other settings/countries and limit the accuracy of the estimate risk for patients.

Moreover, UK specific risk and contemporary equations have not been developed yet and such PJI risk estimates are calculated based on results from different countries (such as Sweden [5]) where treatment algorithms and local medical guidelines may differ from UK. Clinical Practice Research Datalink (CPRD) is a UK government, not-for-profit research service; that collects health records from participating GP practices that have agreed to contribute data to this database related to diagnoses, symptoms, prescriptions, referrals and tests for patients it has provided anonymized primary care data to conduct public health research.[12] CPRD also provide linkage to Office of national statistics (ONS), providing accurate date and cause of death, along with Hospital episode statistics (HES) contains records of secondary care activities including dates, specialty, clinical diagnoses and procedures.[12] Due to some differences in structure and clinical coding in IT systems



For peer review only - http://bmjopen.bmj.com/site/about/guidelines.xhtml

employed by the GP practices,[13] CPRD data are provided as separate databases: CPRD GOLD contains data contributed by practices using Vision® software, [14] while CPRD AURUM contains data from practices using EMIS® Web.[12] In September 2018, CPRD AURUM included records of 19 million patients registered in 738 practices (~ 10% of all English practices), of whom 7 million were alive and actively contributing (13% of the population of England). In 2013, CPRD GOLD contained records from 11 million patients registered in 674 practices; including 4.4 million patients active (alive, currently registered), approximately 6.9% of the UK population.[14] Therefore, they both offer a representative population of UK patients; however when linkage to HES and ONS is employed only the UK regions in England will be represented in the final dataset.

Because of the lack of contemporary and UK specific rick equations for the development of PJI, the objectives of this study were multiples. First, we wanted to compare the characteristics of patients developing PJI in both CPRD databases, to estimate risk equations from individual databases and compare the contribution of each individual covariate in both databases to assess the possibility of pooling participants. Secondly, relevant risk factors were identified from a stepwise regression of the pooled cohorts risk equation and the predictive ability of the newly developed risk equation assessed.

# 5 Methods

## *5.1 Data sources*

Data were obtained from both databases available in the CPRD; Furthermore, CPRD data were linked to HES secondary care data and ONS mortality data.

This study protocol (19_009) was reviewed by the Independent Scientific Advisory Committee (ISAC) and approved in January 2020; a minor amendment was subsequently



For peer review only - http://bmjopen.bmj.com/site/about/guidelines.xhtml

approved on 1st December 2021. Data were extracted in December 2020. The research team had access to only de-identified data therefore the requirement for patient consent was waived.

## 5.2 Study design and population

This was a retrospective study of patients undergoing hip or knee arthroplasty; the index date for inclusion was the day of arthroplasty. Patients had to be aged ≥ 30 years at index date and have arthroplasty after 01/01/2007. Patients were excluded from the study if they were registered on the CPRD for < 6 months prior to index date with the most recent CPRD up-to-standard (UTS) date > 6 months prior to index date.

This research is largely descriptive rather than inferential in nature in that it aims to characterize patterns of PJI and verify the generalizability of existing risk equations. A sample size and power calculation have, therefore, not been undertaken with respect to identifying differences in outcomes between groups.

Eligible patients were identified through OPCS Classification of Interventions and Procedures (OPCS-4) codes [2] for hip or knee arthroplasty in HES records and they were followed-up for 5 years or the first occurrence of: device replaced, death or loss to follow-up. Loss to follow-up was be defined as the date a patient was transferred out of the practice, the date that the practice left the database, or the last recorded event date.

## 5.3 Study measures

Covariate related to patient characteristics were extracted from the CPRD database; surgery properties were derived from the HES database according to OPCS codes (for type of fixation, laterality fixation, grafts ...) and admission codes. Medical history was determined by the presence before index date of disease specific ICD-10 codes in HES or either MedCodeID codes in the CPRD AURUM database or READ codes in CPRD GOLD



For peer review only - http://bmjopen.bmj.com/site/about/guidelines.xhtml

database. Prescription history was assessed from relevant BNF codes reported in the CPRD database.[2] Data cleaning was performed to remove implausible entries such as day of death before index date, BMI < 10 or BMI > 80.

PJI occurrence was assess by the presence of the ICD-10 code T84.5 in the HES database; the date of PJI occurrence was set as the day of hospitalization. To ascertain that PJI diagnosed were affecting the joint of interest as patients could have multiple devices, only a diagnosis of PJI and a record to OPCS any procedure in the joint of interest during the hospitalization were considered.

## 5.4 Survival analysis

Survival curves for each database were fitted with parametric models (Exponential, Weibull, normal, log-normal, logistic, log-logistic) and ranked according to AIC and BIC. Stepwise regression was employed to reduce the number of covariates in the risk equation using AIC as criteria to identify which variable to add/remove or to stop the selection process.

## 5.5 Statistical analysis

Descriptive analyses were generated, characterizing patient demographics, clinical and treatment characteristics. Summary statistics (e.g. mean, standard deviation, standard error, median, inter-quartile range, minimum, and maximum) were calculated for continuous variables (age and BMI) while number and proportion/percentage were calculated for categorical variables. For multivariate regression, age was categorised as ≤ 45, 46–55, 56–65, 66–75, 76–85 and > 85 while BMI was categorised as ≤ 20, 20–25, 26–30, 31–35, 36–50 and > 50. The number and proportion of patients with missing data are also determined for each of the variables of interest, missing data were handled through categorical binning.



For peer review only - http://bmjopen.bmj.com/site/about/guidelines.xhtml

Kaplan-Meier curves were generated, and the log-rank test was used to compare survival curves between the CPRD AURUM and CPRD GOLD databases.

PJI rates were calculated dividing the number of PJI cases observed by the total duration of the follow-up.

Z-test for each factor was performed to compare of the regression coefficients between risk equations developed using CPRD AURUM and CPRD GOLD.[15]

$$Z_i = \frac{\beta_{i,AURUM} - \beta_{i,GOLD}}{\sqrt{SE^2_{i,AURUM} + SE^2_{i,GOLD}}}$$

Where:

$\beta_i$ = regression coefficient for factor *i*

$SE_i$ = Standard error of regression coefficient for factor *i*

The predictive ability of the final risk equation was assess calculating the area under the precision-recall and receiver-operator curves at time-varying follow-ups.

C-statistic was evaluated after different follow-up; 95% confidence intervals were estimated through bootstrapping with 500 replicates for each timepoint.

All data collection, analysis and visualization were performed using R (ver 4.2.2) and relevant packages.[16 17]



For peer review only - http://bmjopen.bmj.com/site/about/guidelines.xhtml

# 6 Results

## 6.1 Patient counts

After linkage with both HES and ONS, records for 330,173 hip of knee arthroplasty, corresponding to 235,249 patients were identified in CPRD AURUM; similarly, CPRD GOLD contained information for 88,458 hip or knee joint subjected to arthroplasty related to 62,672 patients. After applying the inclusion/exclusion criteria the records of 283,789 joint (221,826 patients) were identified in CPRD AURUM and 71,071 joint (54,833 patients) in CPRD GOLD (Table S1).

In patients indexed in either CPRD AURUM or GOLD, the most common reason for not completing the study observation period (5 years) was the termination of the data collection, 27.06% and 35.45% in CPRD AURUM and GOLD, respectively. The second most common reason in CPRD AURUM was transfer out of the CPRD database (in 12.63% of cases) while in CPRD GOLD it was death (in 10.52% of cases); replacement of the implanted device in the first 5 years after surgery occurred in about 1.9% of cases in both databases. A diagnosis of PJI in the first years after implant occurred in 1,402 joint (0.49%) in CPRD AURUM and 288 (0.40%) joints in CPRD GOLD (Table S2).

## 6.2 Baseline characteristics

The demographic and basic characteristics of patients in both CPRD databases were generally similar for all covariates tested (e.g. gender, age, BMI, alcohol and smoking status) (Table S3); the level on unknown BMI was much greater in GOLD than AURUM. The characteristics of the arthroplasty exhibited similar distribution in both databases (Table S4). Patients undergoing hip or knee arthroplasty had similar prevalence of most of the diseases included in this study, also RA and cancer had different distributions in AURUM and GOLD

For peer review only

(Table S5). Moreover, the also history of prescriptions in patients with hip and knee arthroplasty in CPRD AURUM and GOLD were comparable (Table S6).

## 6.3 PJI occurrence

Unadjusted Kaplan-Meier curve for time to PJI diagnosis showed a decrease hazard rate with time; 0.21% and 0.30% of joints in CPRD AURUM developed PJI in the first 6 and 12 months, respectively. After 2 and 4 years the proportion of joints with PJI was 0.41% and 0.54% respectively. The probability of not developing PJI in the joints from CPRD GOLD was 0.15%, 0.23%, 0.37% and 0.47% after 6 months, 1 year, 2 years and 4 years respectively. The log-rank test between the two curves had *P* value < 0.001 (Figure 1a). Likewise, the Kaplan-Meier curves for time to PJI in the two CPRD databases exhibited comparable patterns with *P* values were generally < 0.001 (Figure 1b and c). The distribution of the PJI rate different across the different England regions defined in CPRD ranging from ~100 PJI cases/100000 patients year in the East Midlands to ~180 PJI cases/100000 patients year in the North East (Figure 1d).

## 6.4 Risk equations

Survival curves of time to PJI diagnosis in CPRD GOLD and CPRD AURUM were fitted independently with parametric models; for both databases the log-normal distribution returned the best fitting (e.g. lowest AIC, lowest BIC and highest loglik) (Table S7 and Table S8). The coefficients of the log-normal parametric models for both databases were compared with Z-test and generally *P* values were > 0.05 (Figure 2 and Table S9).

The risk equations using the log-normal survival model developed using the pooled cohorts performance in categorizing a patients as at risk of PJI or not over different follow-up times increased with longer periods as revealed by the area under the precision-recall curve; the



For peer review only - http://bmjopen.bmj.com/site/about/guidelines.xhtml

area under the receiver operator curve (AUC) and C-statistic, did not show exhibit the same increase with longer follow-up instead (Figure 3 and Table S10).

The stepwise variable selection was employed to reduce the number of covariates in the risk equation to those most significant. The final list of coefficients revealed that gender, age, BMI were patient characteristics risk factors for PJI. The type of surgery (primary or secondary), admission through A&E or elective and the resurfacing of the patella were the risk factors related to the surgical procedure. A history of AF, PE, diabetes, RA, MI and hypertension were all increasing the risk of PJI, while a history of osteoporosis was linked to a reduction of such risk. Previous PJIs were also further medical risk factors for a further PJI. The use of steroids, NSAID, antifungal drug and DOAC before arthroplasty also were risk factors (Table 1). Examples of the fitting of the Kaplan-Meier curves with the log-normal stepwise regression model showed a general good agreement (Figure 4).

# 7 Discussion

PJI is a relative rare but highly impactful outcome, both in terms of patients' quality of life and resources consumption,[3,4,18-21] of arthroplasty. Prevention or early identification of PJI through stringent medical follow-up is not a viable option because of the large population undergoing hip and knee arthroplasty and the potential long follow-up time, as shown here and in multiple studies PJI can be diagnosed even years after surgery.[2,22,23]

Patients identified in CPRD AURUM were statistically different than those identified in CPRD GOLD (Table S3-S6) likely as a consequence of the different geographical coverage of England by the two databases associated to the population characteristics variation around the country in term of age and diseases.[24,25] However, as expected the impact of almost all covariates on the risk of developing PJI was the same ($p > 0.05$) when estimated with the

For peer review only



For peer review only - http://bmjopen.bmj.com/site/about/guidelines.xhtml

same parametric model. The hazard of PJI (Figure 1) exhibited a monotonically decreasing profile typical of other studies investigating such occurrence [19 26-29] and, in both databases, the log-normal model returned the best fitting of the data as this model is suitable for this type of risk profile.[30] Similarly, the log-logistic, another model suitable for decreasing hazard,[31] also returned good fitting (Table S7b and Table S8).

As regression coefficients in the risk equations obtained from both databases were not statistically different for all covariates included in this study, both cohorts were pooled to increase the population size and, consequently, reduce the error associated with the coefficients estimation.[32 33] The regression model built using the pooled cohort performance was evaluated using the area under the receiver operator curve that represents the balance between sensitivity (correctly identified cases) and specificity (correctly identified non-cases). The model performance did not significantly vary when applied to the likelihood of developing PJI over different follow-up time, probability due to the unbalance nature of the cohort in terms of patients developing PJI and patients not developing PJI. The area under the precision-recall curve (Figure 3b) instead is not impacted by unbalanced samples and the model performed better with increasing follow-up time.[34]

Stepwise selection is a classic variable reduction technique and was employed here to simplify the model minimizing the loss of performance. Negative coefficients indicate an increased risk of PJI compared to the reference, analogously positive coefficients indicate a reduced risk of PJI compared to the reference. The greater the absolute value of the coefficient the greater the impact of the risk of PJI. The risk factors identified through this approach are in line with those commonly associated to PJI (Table 1) as male gender, increased BMI and a previous PJI in the same joint or in another joint were retained in the list of covariates after stepwise selection.[2 9 28 35] Smoking is sometimes reported as a risk factor PJI [9 36] but such sometime the opposite is reported [37] as here where patient's smoking status



For peer review only - http://bmjopen.bmj.com/site/about/guidelines.xhtml

was not included in the final model. Moreover, the active role of numerous medications provides a range of modifiable risk factors that could also inform surgery scheduling when arthroplasty is an elective procedure as the risk of PJI after NSAID and antifungal use decrease with increasing interval between the last use and the arthroplasty.

The model performance after stepwise selection was still high as shown by the C-statistic (Table S10) and the visual fit of the curves (Figure 4) demonstrating how the retained covariates are those pivotal in determining a patients risk to develop PJI after arthroplasty.

The model developed here provides an objective quantification of the risk to develop PJI after hip or knee arthroplasty allowing personalized pre-surgery drug prescription consideration and follow-up care.

# 8 Conclusions

Patients undergoing hip or knee arthroplasty present in CPRD AURUM and CPRD GOLD exhibit different sociodemographic characteristics and medical/drug history. However, the coefficients of parametric models, fitted to the survival curves were not statistically different allowing for pooling cohorts.

The model developed here demonstrated a good ability to identify patients at risk (based on C-statistic) and is based on the UK specific population. Such capability would allow the NHS to review the current guidelines, potentially devising a targeted program of medical treatment pre-surgery, when appropriate, and of close monitoring to a small fraction of the patients after undergoing hip or knee arthroplasty allowing to reduce the incidence and impact of PJI.

The newly developed risk equations will also provide contemporary inputs for the economic evaluation of new technologies for the prevention of PJI supporting health technology assessment (HTA) from The National Institute for Health and Care Excellence (NICE).



For peer review only - http://bmjopen.bmj.com/site/about/guidelines.xhtml

# 9 Data Availability

This study is based in part on data from the Clinical Practice Research Datalink obtained under license from the UK Medicines and Healthcare products Regulatory Agency. The data is provided by patients and collected by the NHS as part of their care and support. The interpretation and conclusions contained in this study are those of the author/s alone. The authors did not have any special access privileges that others would not have. Other researchers can apply for access to the raw data at the following URL: https://cprd.com/data-access/.

# 10 Patient and public involvement

Patients and/or the public were not involved in the design, or conduct, or reporting, or dissemination plans of this research.

# 11 Competing interests statement

The authors have not conflict of interest to declare in the relation to this work.

# 12 Authors contribution

SP and PP conceived the idea for the study. SP carried out the statistical analysis and wrote the first draft of the manuscript. PP supervised the study and analysis. All authors reviewed and revised the manuscript. PP acts as guarantor.



For peer review only - http://bmjopen.bmj.com/site/about/guidelines.xhtml

# 13 Funding

This work has also been supported by Pathfinder Fund of Wellcome Trust (WT109455MA). The funder had no role in the study design, data collection and analysis, decision to publish, or preparation of the manuscript.


For peer review only

# 14 References

1. Osmon DR, Berbari EF, Berendt AR, et al. Diagnosis and management of prosthetic joint infection: clinical practice guidelines by the Infectious Diseases Society of America. *Clinical infectious diseases : an official publication of the Infectious Diseases Society of America* 2013;56(1):e1-e25. doi: 10.1093/cid/cis803 [published Online First: 2012/12/12]
2. Perni S, Bojan B, Prokopovich P. A retrospective study of risk factors, causative micro-organisms and healthcare resources consumption associated with prosthetic joint infections (PJI) using the Clinical Practice Research Datalink (CPRD) Aurum database. *PLOS ONE* 2023;18(3):e0282709. doi: 10.1371/journal.pone.0282709
3. Moore AJ, Blom AW, Whitehouse MR, et al. Deep prosthetic joint infection: a qualitative study of the impact on patients and their experiences of revision surgery. *BMJ Open* 2015;5(12):e009495. doi: 10.1136/bmjopen-2015-009495 [published Online First: 2015/12/09]
4. Kallala R. F., Vanhegan I. S, Ibrahim M. S., et al. Financial analysis of revision knee surgery based on NHS tariffs and hospital costs. *The Bone & Joint Journal* 2015;97-B(2):197-201. doi: 10.1302/0301-620x.97b2.33707
5. Bülow E, Hahn U, Andersen IT, et al. Prediction of Early Periprosthetic Joint Infection After Total Hip Arthroplasty. *Clin Epidemiol* 2022;14:239-53. doi: 10.2147/clep.S347968 [published Online First: 20220304]
6. Kuo F-C, Hu W-H, Hu Y-J. Periprosthetic Joint Infection Prediction via Machine Learning: Comprehensible Personalized Decision Support for Diagnosis. *The Journal of Arthroplasty* 2022;37(1):132-41. doi: https://doi.org/10.1016/j.arth.2021.09.005
7. Berbari E, Osmon D, Lahr B, et al. The Mayo Prosthetic Joint Infection Risk Score: Implication for Surgical Site Infection Reporting and Risk Stratification. *Infection control and hospital epidemiology : the official journal of the Society of Hospital Epidemiologists of America* 2012;33:774-81. doi: 10.1086/666641
8. Klemt C, Yeo I, Harvey M, et al. The Use of Artificial Intelligence for the Prediction of Periprosthetic Joint Infection Following Aseptic Revision Total Knee Arthroplasty. *J Knee Surg* 2023(EFirst) doi: 10.1055/s-0043-1761259
9. Kunutsor SK, Whitehouse MR, Blom AW, et al. Systematic review of risk prediction scores for surgical site infection or periprosthetic joint infection following joint arthroplasty. *Epidemiology & Infection* 2017;145(9):1738-49. doi: 10.1017/S0950268817000486 [published Online First: 2017/03/07]
10. Taur SR. Observational designs for real-world evidence studies. *Perspect Clin Res* 2022;13(1):12-16. doi: 10.4103/picr.picr_217_21 [published Online First: 20220106]
11. Rassen JA, Blin P, Kloss S, et al. High-dimensional propensity scores for empirical covariate selection in secondary database studies: Planning, implementation, and reporting. *Pharmacoepidemiology and Drug Safety* 2023;32(2):93-106. doi: https://doi.org/10.1002/pds.5566
12. Wolf A, Dedman D, Campbell J, et al. Data resource profile: Clinical Practice Research Datalink (CPRD) Aurum. *International Journal of Epidemiology* 2019;48(6):1740-40g. doi: 10.1093/ije/dyz034
13. CPRD. Primary care data for public health research [Available from: https://cprd.com/primary-care-data-public-health-research accessed 15 March 2023.
14. Herrett E, Gallagher AM, Bhaskaran K, et al. Data Resource Profile: Clinical Practice Research Datalink (CPRD). *International Journal of Epidemiology* 2015;44(3):827-36. doi: 10.1093/ije/dyv098
15. Barzi F, Patel A, Gu D, et al. Cardiovascular risk prediction tools for populations in Asia. *J Epidemiol Community Health* 2007;61(2):115-21. doi: 10.1136/jech.2005.044842
16. R Core Team. R: A language and environment for statistical computing. R Foundation for Statistical Computing. Vienna, Austria., 2019.
17. Therneau T. A Package for Survival Analysis in S. version 2.38. 2015



For peer review only - http://bmjopen.bmj.com/site/about/guidelines.xhtml

18. Bozic KJ, Kamath AF, Ong K, et al. Comparative Epidemiology of Revision Arthroplasty: Failed THA Poses Greater Clinical and Economic Burdens Than Failed TKA. *Clinical orthopaedics and related research* 2015;473(6):2131-8. doi: 10.1007/s11999-014-4078-8 [published Online First: 2014/12/04]

19. Kurtz SM, Ong KL, Lau E, et al. Prosthetic joint infection risk after TKA in the Medicare population. *Clinical orthopaedics and related research* 2010;468(1):52-6. doi: 10.1007/s11999-009-1013-5 [published Online First: 20090808]

20. Li T, Zhang H, Chan PK, et al. Risk factors associated with surgical site infections following joint replacement surgery: a narrative review. *Arthroplasty* 2022;4(1):11. doi: 10.1186/s42836-022-00113-y [published Online First: 2022/05/01]

21. Garfield K, Noble S, Lenguerrand E, et al. What are the inpatient and day case costs following primary total hip replacement of patients treated for prosthetic joint infection: a matched cohort study using linked data from the National Joint Registry and Hospital Episode Statistics. *BMC Med* 2020;18(1):335-35. doi: 10.1186/s12916-020-01803-7

22. Tande AJ, Patel R. Prosthetic joint infection. *Clinical microbiology reviews* 2014;27(2):302-45. doi: 10.1128/cmr.00111-13 [published Online First: 2014/04/04]

23. Huotari K, Peltola M, Jämsen E. The incidence of late prosthetic joint infections: a registry-based study of 112,708 primary hip and knee replacements. *Acta orthopaedica* 2015;86(3):321-5. doi: 10.3109/17453674.2015.1035173 [published Online First: 20150327]

24. DIgital N. Patients Registered at GP practices in England as at 1 March 2023 2023 [Available from: https://digital.nhs.uk/data-and-information/publications/statistical/patients-registered-at-a-gp-practice/march-2023.

25. digital N. CCG Outcomes Indicator Set - October 2022 2022 [Available from: https://digital.nhs.uk/data-and-information/publications/statistical/ccg-outcomes-indicator-set/october-2022.

26. Iannotti F, Prati P, Fidanza A, et al. Prevention of Periprosthetic Joint Infection (PJI): A Clinical Practice Protocol in High-Risk Patients. *Trop Med Infect Dis* 2020;5(4) doi: 10.3390/tropicalmed5040186 [published Online First: 2020/12/17]

27. Izakovicova P, Borens O, Trampuz A. Periprosthetic joint infection: current concepts and outlook. *EFORT Open Rev* 2019;4(7):482-94. doi: 10.1302/2058-5241.4.180092 [published Online First: 20190729]

28. Lenguerrand E, Whitehouse MR, Beswick AD, et al. Description of the rates, trends and surgical burden associated with revision for prosthetic joint infection following primary and revision knee replacements in England and Wales: an analysis of the National Joint Registry for England, Wales, Northern Ireland and the Isle of Man. *BMJ Open* 2017;7(7):e014056. doi: 10.1136/bmjopen-2016-014056 [published Online First: 2017/07/13]

29. Pulido L, Ghanem E, Joshi A, et al. Periprosthetic joint infection: the incidence, timing, and predisposing factors. *Clinical orthopaedics and related research* 2008;466(7):1710-15. doi: 10.1007/s11999-008-0209-4 [published Online First: 2008/04/18]

30. Gupta RC, Kannan N, Raychaudhuri A. Analysis of lognormal survival data. *Mathematical Biosciences* 1997;139(2):103-15. doi: https://doi.org/10.1016/S0025-5564(96)00133-2

31. Bennett S. Log-Logistic Regression Models for Survival Data. *Journal of the Royal Statistical Society Series C: Applied Statistics* 2018;32(2):165-71. doi: 10.2307/2347295

32. Khera R, Pandey A, Ayers CR, et al. Performance of the Pooled Cohort Equations to Estimate Atherosclerotic Cardiovascular Disease Risk by Body Mass Index. *JAMA Network Open* 2020;3(10):e2023242-e42. doi: 10.1001/jamanetworkopen.2020.23242

33. Vasan RS, van den Heuvel E. Differences in estimates for 10-year risk of cardiovascular disease in Black versus White individuals with identical risk factor profiles using pooled cohort equations: an in silico cohort study. *The Lancet Digital Health* 2022;4(1):e55-e63. doi: 10.1016/S2589-7500(21)00236-3

34. Max Kuhn, Johnson K. Applied Predictive Modeling: Springer New York, NY 2013.

35. Lenguerrand E, Whitehouse MR, Beswick AD, et al. Risk factors associated with revision for prosthetic joint infection after hip replacement: a prospective observational cohort study. *The Lancet Infectious Diseases* 2018;18(9):1004-14. doi: https://doi.org/10.1016/S1473-3099(18)30345-1

For peer review only



For peer review only - http://bmjopen.bmj.com/site/about/guidelines.xhtml

36. Bojan B, Perni S, Prokopovich P. Systematic Review and Meta-Analysis of Tobacco Use as a Risk Factor for Prosthetic Joint Infection After Total Hip Replacement. *Arthroplast Today* 2020;6(4):959-71. doi: 10.1016/j.artd.2020.07.011 [published Online First: 20201207]
37. Ren X, Ling L, Qi L, et al. Patients' risk factors for periprosthetic joint infection in primary total hip arthroplasty: a meta-analysis of 40 studies. *BMC Musculoskeletal Disorders* 2021;22(1):776. doi: 10.1186/s12891-021-04647-1

For peer review only



For peer review only - http://bmjopen.bmj.com/site/about/guidelines.xhtml

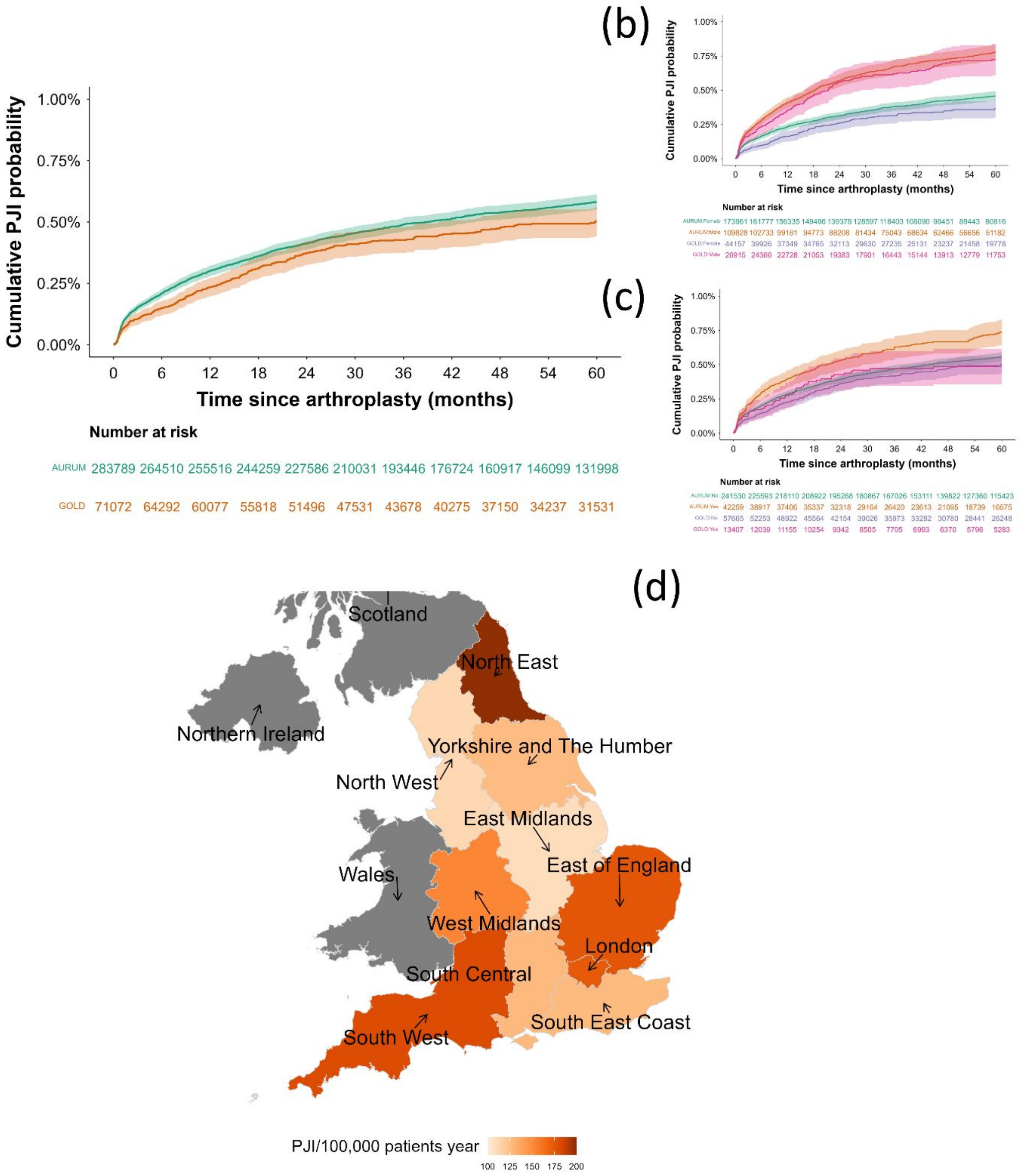


**Figure 1.** Examples of unadjusted Kaplan-Meier curves of cumulative risk of PJI (and 95% confidence intervals) for the entire cohorts (a) and stratified based on gender (b) and history of diabetes at baseline (c). Geographical distribution of PJI rates in the CPRD regions of England (d)

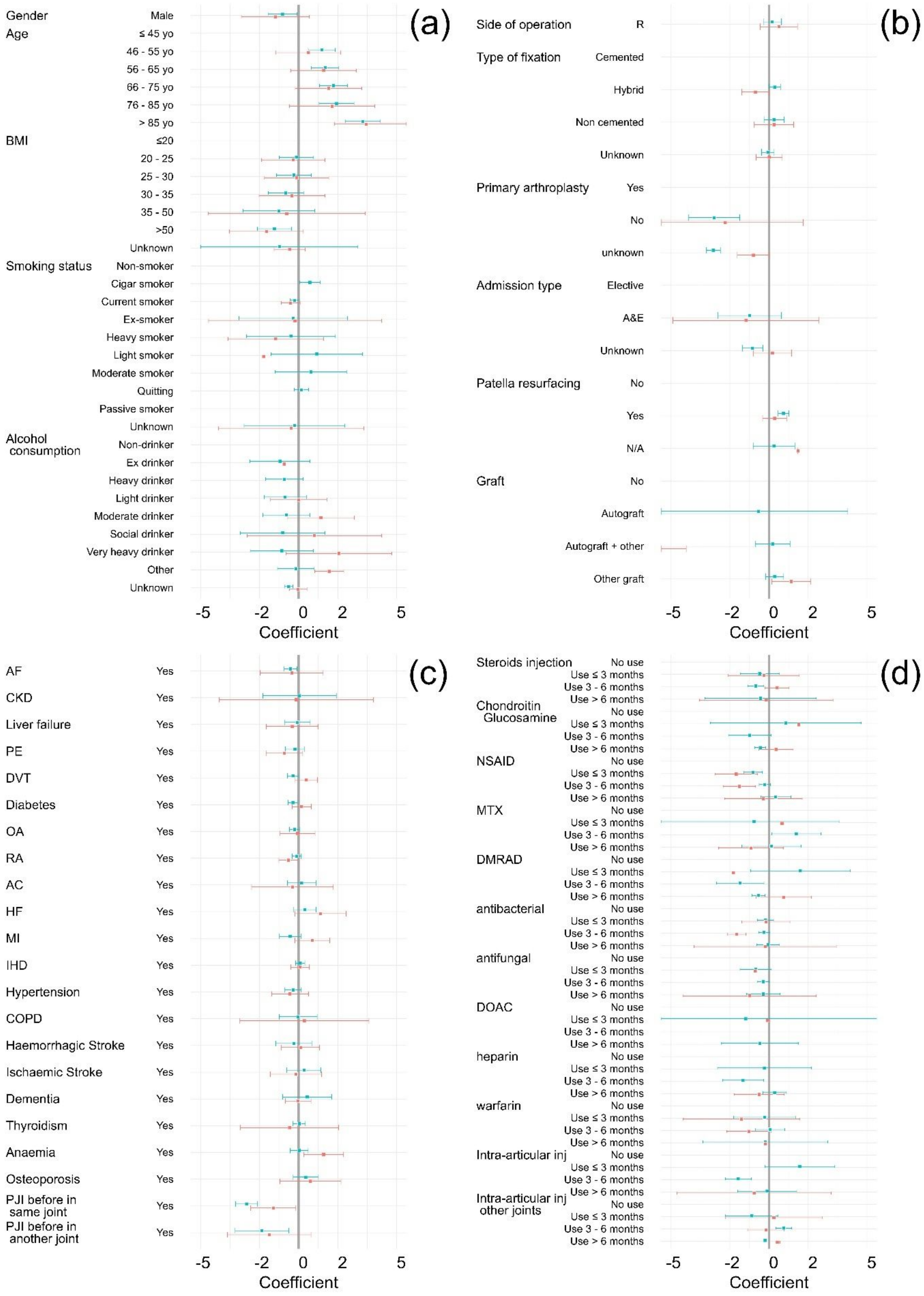


**Figure 2.** Coefficients for time to PJI multivariate regression with log-normal model for data from CPRD GOLD (——) and AURUM database (——).

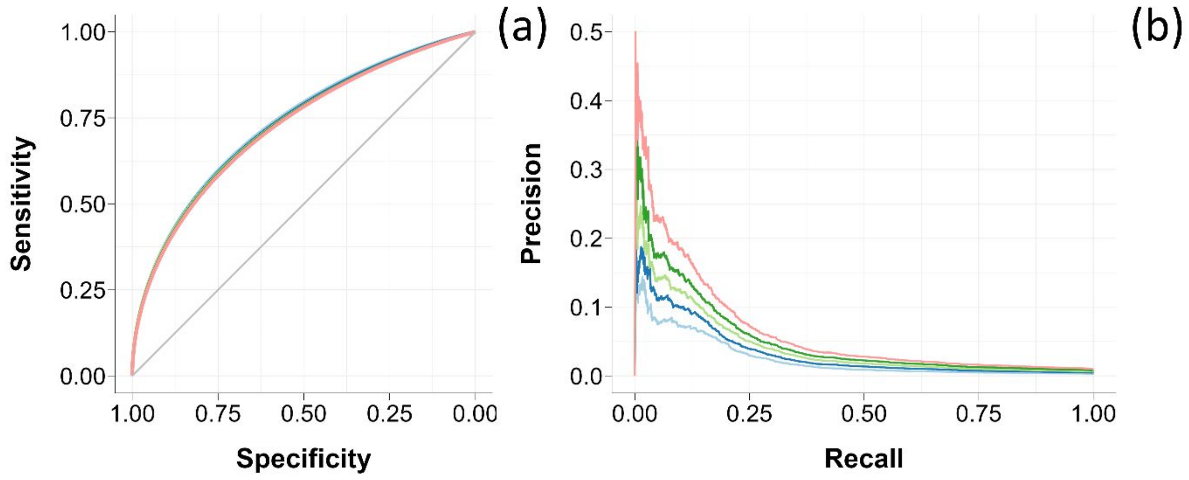


**Figure 3.** Time dependent (—— 1 year —— 2 years —— 3 years —— 4 years —— 5 years) Receiver operating curve (ROC) (a) and Precision-Recall curves (b) of pooled log-normal equation.

For peer review only

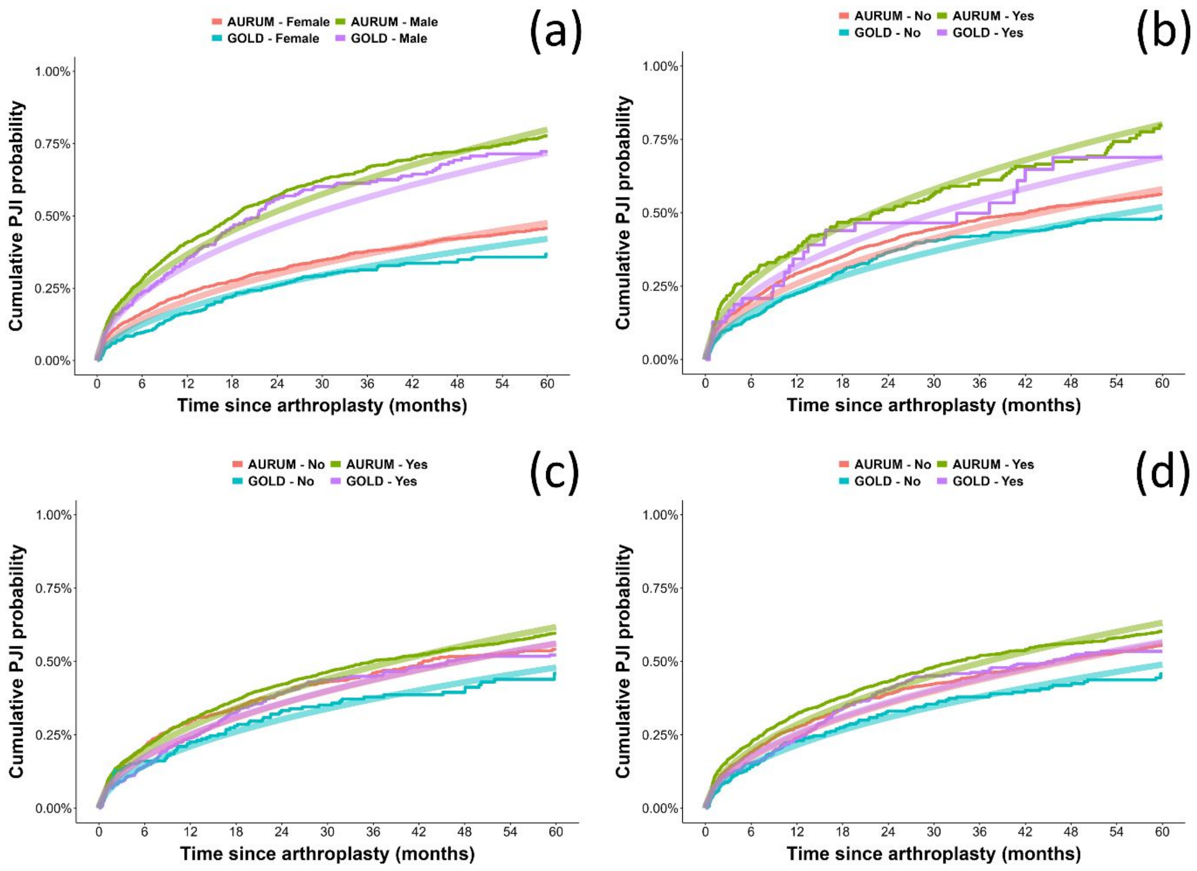


**Figure 4.** Examples of Kaplan-Meier curves of risk of PJI and prediction using pooled log-normal parametric model after stepwise selection for both AURUM and GOLD CPRD database (a) gender and history of (b) Atrial Fibrillation, (c) Osteo-arthritis or (d) hypertension at baseline.

eview only



For peer review only - http://bmjopen.bmj.com/site/about/guidelines.xhtml

**Table 1.** Coefficients and SE of pooled log-normal parametric model of time to PJI after stepwise regression.

| Variable | Coefficient | SE |
|---|---|---|
| **Scale** | 1.709 | 0.022 |
| **Intercept** | 21.593 | 0.587 |
| **Male** | -0.886 | 0.102 |
| **Age** | | |
| ≤ 45 | Reference | |
| 46 – 55 | 1.026 | 0.345 |
| 56 – 65 | 1.321 | 0.319 |
| 66 – 75 | 1.700 | 0.317 |
| 76 – 85 | 1.854 | 0.325 |
| > 85 | 3.283 | 0.409 |
| **BMI** | | |
| ≤ 20 | Reference | |
| 20 – 25 | -0.185 | 0.402 |
| 26 – 30 | -0.253 | 0.392 |
| 31 – 35 | -0.633 | 0.396 |
| 36 – 50 | -0.976 | 0.403 |
| > 50 | -1.296 | 0.846 |
| Unknown | -0.948 | 0.389 |
| **Operation on Right Side** | 0.201 | 0.097 |
| **Primary arthroplasty** | | |
| Yes | Reference | |
| No | -2.706 | 0.144 |
| Unknown | -2.700 | 0.616 |
| **Admission type** | | |
| Elective | Reference | |
| A&E | -1.009 | 0.162 |
| Unknown | -0.666 | 0.757 |
| **Patella resurfacing** | | |
| No | Reference | |
| Yes | 0.688 | 0.236 |
| N/A | 0.582 | 0.108 |
| **AF** | -0.565 | 0.176 |
| **PE** | -0.445 | 0.285 |
| **Diabetes** | -0.225 | 0.132 |
| **RA** | -0.215 | 0.113 |
| **MI** | -0.296 | 0.168 |
| **Hypertension** | -0.312 | 0.105 |
| **Osteoporosis** | 0.365 | 0.214 |
| **PJI before in same joint** | -2.585 | 0.302 |
| **PJI before in another joint** | -1.818 | 0.257 |

For peer review only



For peer review only - http://bmjopen.bmj.com/site/about/guidelines.xhtml

| | | |
|---|---|---|
| **Steroids injection** | | |
| No Use | Reference | |
| Use < 3 months | -0.486 | 0.593 |
| Use 3 - 6 months | -0.485 | 0.445 |
| Use > 6 months | -0.407 | 0.167 |
| **NSAID** | | |
| No Use | Reference | |
| Use < 3 months | -0.900 | 0.130 |
| Use 3 - 6 months | -0.395 | 0.210 |
| Use > 6 months | 0.301 | 0.124 |
| **Antifungal** | | |
| No Use | Reference | |
| Use < 3 months | -0.725 | 0.287 |
| Use 3 - 6 months | -0.309 | 0.401 |
| Use > 6 months | -0.416 | 0.131 |
| **DOAC** | | |
| No Use | Reference | |
| Use < 3 months | -0.994 | 0.3975 |
| Use 3 - 6 months | 25.301 | 5079.8 |
| Use > 6 months | -0.108 | 0.705 |

464

For peer review only

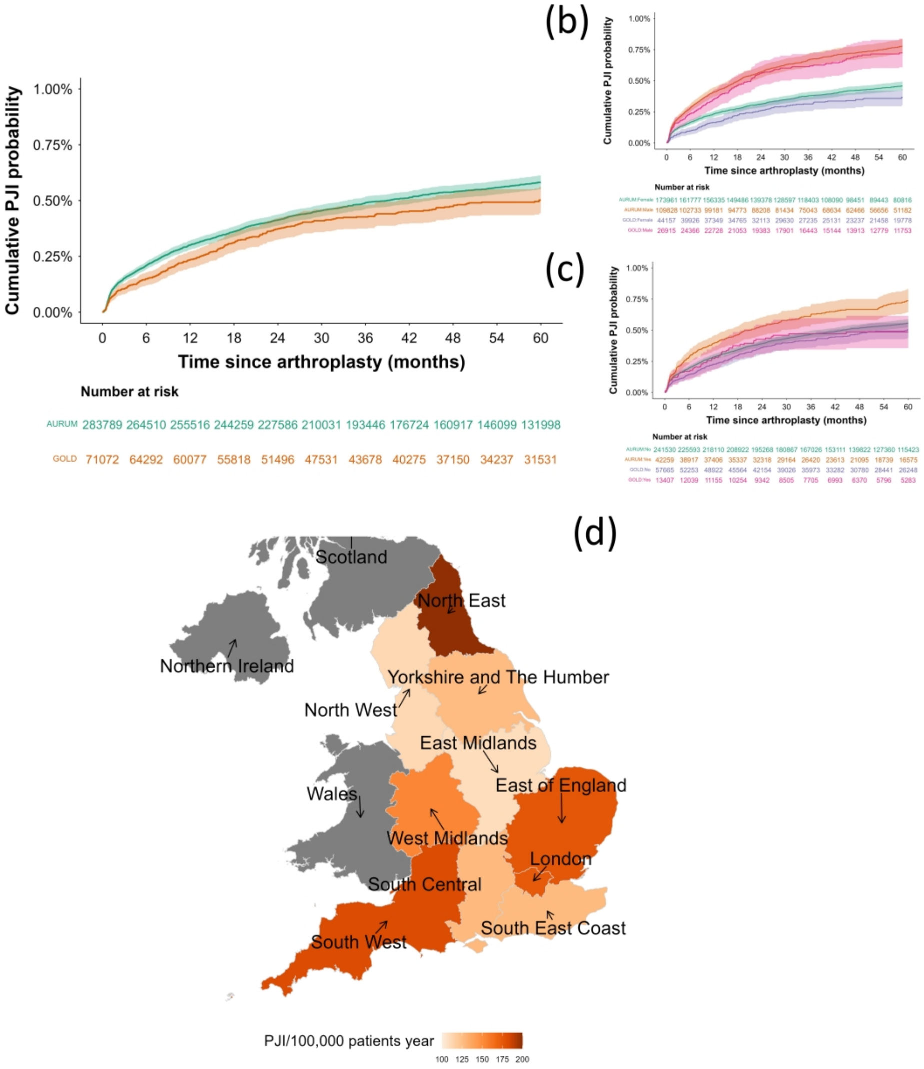


Figure 1

1871x2374mm (57 x 57 DPI)

Unable to Convert Image

The dimensions of this image (in pixels) are too large to be converted. For this image to convert, the total number of pixels (height x width) must be less than 40,000,000 (40 megapixels).

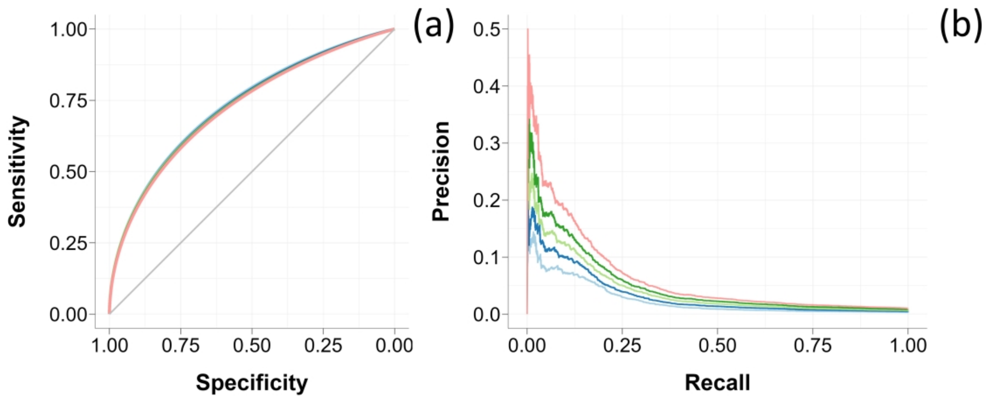


889x376mm (118 x 118 DPI)

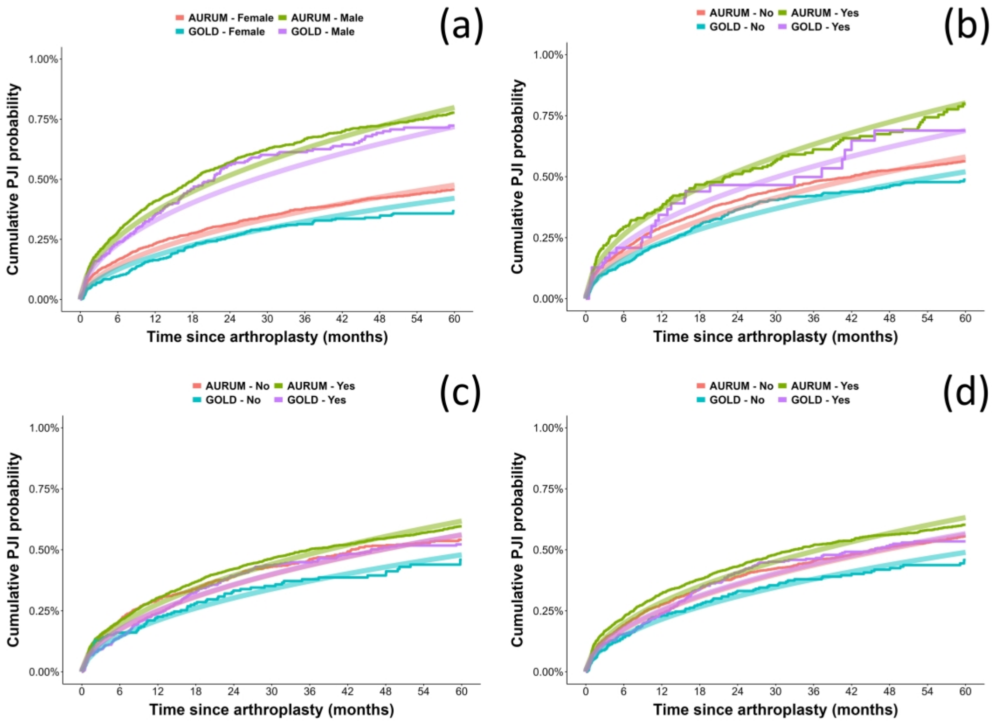


904x678mm (118 x 118 DPI)

# Risk equations for prosthetic joint infections (PJI) using the Clinical Practice Research Datalink (CPRD) AURUM and GOLD database

*Stefano Perni[1] and Polina Prokopovich[1,]**

School of Pharmacy and Pharmaceutical Sciences, Cardiff University, Cardiff, UK

# Supplementary Material

For peer review only

**Table S 1.** Flow of patients through initial data extraction.

| | AURUM | | GOLD | |
|---|---|---|---|---|
| | Number of joints | Number of patients | Number of joints | Number of patients |
| Knee or hip replacement in database | 330,173 | 235,249 | 88,458 | 62,672 |
| Over 31 years old at index date | 329,720 | 235,071 | 88,268 | 62,584 |
| Arthroplasty surgery after 2007 | 288,124 | 223,450 | 71,467 | 54,888 |
| With minimum 6 months lookback | 284,048 | 222,060 | 71,467 | 54,888 |
| After removing implausible data | 283,789 | 221,826 | 71,072 | 54,833 |

For peer review only

**Table S 2.** Number of joints not compleating the observation study period (5 years) and reason for loss during follow-up.

| Reason for not completing study period | AURUM | | GOLD | |
|---|---|---|---|---|
| | n | Frequency | n | Frequency |
| Developing PJI | 1,402 | 0.49% | 288 | 0.40 % |
| Death | 32,981 | 11.62% | 7,475 | 10.52% |
| Last collection date before end of observation period | 76,800 | 27.06% | 25,195 | 35.45% |
| Device replaced for any reason | 5,371 | 1.89% | 1,362 | 1.92% |
| Transferred out of CPRD | 35,831 | 12.63% | 5,337 | 7.51% |

For peer review only

**Table S 3.** Characteristics of patients at the time of hip or knee implant surgery.

| Variable | All | AURUM | GOLD |
|---|---|---|---|
| **Gender** | | | |
| Female | 218,118 (61.47%) | 173,961 (61.30 %) | 44,157 (62.13 %) |
| Male | 136,743 (38.53%) | 109,828 (38.70 %) | 26,915 (37.87 %) |
| **Age** | | | |
| ≤ 45 | 4,945 (1.39%) | 4,074 (1.44 %) | 871 (1.23 %) |
| 46 – 55 | 21,680 (6.11%) | 17,512 (6.17 %) | 4,168 (5.86 %) |
| 56 – 65 | 68,237 (19.23%) | 54,632 (19.25 %) | 13,605 (19.14 %) |
| 66 – 75 | 121,185 (34.15%) | 96,747 (34.09 %) | 24,438 (34.38 %) |
| 76 – 85 | 102,610 (28.92%) | 81,892 (28.86 %) | 20,718 (29.15 %) |
| > 85 | 36,204 (10.20%) | 28,932 (10.19 %) | 7,272 (10.23 %) |
| Mean (SD) | 71.92 | 71.88 (10.98) | 72.04 (10.81) |
| Median | 72 | 72 | 73 |
| IQR | 65.00 - 80.00 | 65.00 - 80.00 | 65.00 - 80.00 |
| Min, max | 31.00, 109.00 | 31.00, 109.00 | 31.00, 107.00 |
| **BMI** | | | |
| ≤ 20 | 10,773 (3.04%) | 7,694 (2.71 %) | 3,079 (4.33 %) |
| 20 – 25 | 54,649 (15.40%) | 39,004 (13.74 %) | 15,645 (22.01 %) |
| 26 – 30 | 94,902 (26.74%) | 70,293 (24.77 %) | 24,609 (34.63 %) |
| 31 – 35 | 65,688 (18.51%) | 50,075 (17.65 %) | 15,613 (21.97 %) |
| 36 – 50 | 39,618 (11.16%) | 30,963 (10.91 %) | 8,655 (12.18 %) |
| > 50 | 819 (0.23%) | 698 (0.25 %) | 121 (0.17 %) |
| Unknown | 88,412 (24.91%) | 85,062 (29.97 %) | 3,350 (4.71 %) |
| Mean (SD) | 29.18 | 29.37 (6.02) | 28.61 (5.74) |
| Median | 28.6 | 28.74 | 28 |
| IQR | 25.10 - 32.60 | 25.30 - 32.86 | 24.70 - 32.00 |
| Min, max | 10.46, 73.00 | 10.46, 73.00 | 14.50, 67.50 |
| Smoking status | | | |
| Non-smoker | 193,403 (54.50%) | 153,344 (54.03 %) | 40,059 (56.36 %) |
| Cigar | 223 (0.06%) | 223 (0.08 %) | 0 (0.00 %) |
| Current | 18,120 (5.11%) | 12,112 (4.27 %) | 6,008 (8.45 %) |
| Ex-smoker | 112,673 (31.75%) | 88,563 (31.21 %) | 24,110 (33.92 %) |
| Heavy | 405 (0.11%) | 307 (0.11 %) | 98 (0.14 %) |
| Light | 1,067 (0.30%) | 782 (0.28 %) | 285 (0.40 %) |
| Moderate | 704 (0.20%) | 704 (0.25 %) | 0 (0.00 %) |
| Quitting | 886 (0.25%) | 886 (0.31 %) | 0 (0.00 %) |
| Unknown | 15 (0.00%) | 0 (0.00 %) | 15 (0.02 %) |
| **Alcohol consumption** | | | |
| Non-drinker | 12,420 (3.50%) | 4,826 (1.70 %) | 7,594 (10.68 %) |
| Ex drinker | 593 (0.17%) | 418 (0.15 %) | 175 (0.25 %) |
| Heavy drinker | 1,687 (0.48%) | 1,580 (0.56 %) | 107 (0.15 %) |
| Light drinker | 64,437 (18.16%) | 52,731 (18.58 %) | 11,706 (16.47 %) |

For peer review only

| | | | |
|---|---|---|---|
| Moderate drinker | 12,128 (3.42%) | 9,282 (3.27 %) | 2,846 (4.00 %) |
| Social drinker | 6,594 (1.86%) | 4,781 (1.68 %) | 1,813 (2.55 %) |
| Very heavy | 561 (0.16%) | 561 (0.20 %) | 0 (0.00 %) |
| Other | 525 (0.15%) | 0 (0.00 %) | 525 (0.74 %) |
| Unknown | 2,400 (0.68%) | 1,732 (0.61 %) | 668 (0.94 %) |

For peer review only

**Table S 4.** Characteristics of hip or knee implant surgery.

| Variable | All | AURUM | GOLD |
|---|---|---|---|
| **Joint replaced** | | | |
| Femur | 47,533 (13.39%) | 38,159 (13.45 %) | 9,374 (13.19 %) |
| Hip | 154,941 (43.66%) | 124,265 (43.79 %) | 30,676 (43.16 %) |
| Knee | 152,387 (42.94%) | 121,365 (42.77 %) | 31,022 (43.65 %) |
| **Laterality of operation** | | | |
| Left | 166,860 (47.02%) | 133,269 (46.96 %) | 33,591 (47.26 %) |
| Right | 188,001 (52.98%) | 150,520 (53.04 %) | 37,481 (52.74 %) |
| **Fixation method** | | | |
| Cemented | 233,475 (65.79%) | 185,253 (65.28 %) | 48,222 (67.85 %) |
| Hybrid | 27,819 (7.84%) | 22,349 (7.88 %) | 5,470 (7.70 %) |
| Non cemented | 81,064 (22.84%) | 66,507 (23.44 %) | 14,557 (20.48 %) |
| Unknown | 12,503 (3.52%) | 9,680 (3.41 %) | 2,823 (3.97 %) |
| **Primary arthroplasty** | | | |
| Yes | 327,606 (92.32%) | 262,247 (92.41 %) | 65,359 (91.96 %) |
| No | 26,362 (7.43%) | 20,817 (7.34 %) | 5,545 (7.80 %) |
| Unknown | 893 (0.25%) | 725 (0.26 %) | 168 (0.24 %) |
| **Admission type** | | | |
| Elective | 294,138 (82.89%) | 234,961 (82.79 %) | 59,177 (83.26 %) |
| A&E | 59,815 (16.86%) | 48,156 (16.97 %) | 11,659 (16.40 %) |
| Unknown | 908 (0.26%) | 672 (0.24 %) | 236 (0.33 %) |
| **Patella resurfacing (* only for knee replacement surgeries)** | | | |
| No | 130,735 (36.84%) | 103,244 (36.38 %) | 27,491 (38.68 %) |
| Yes | 21,652 (6.10%) | 18,121 (6.39 %) | 3,531 (4.97 %) |
| **Graft** | | | |
| No | 349,743 (98.56%) | 279,681 (98.55 %) | 70,062 (98.58 %) |
| Autograft | 2,285 (0.64%) | 1,857 (0.65 %) | 428 (0.60 %) |
| Autograft + other | 88 (0.02%) | 76 (0.03 %) | 12 (0.02 %) |
| Other graft | 2,745 (0.77%) | 2,175 (0.77 %) | 570 (0.80 %) |

For peer review only

**Table S 5.** Medical history of patients at the time of hip or knee implant surgery.

| Variable | All | AURUM | GOLD |
|---|---|---|---|
| **Atrial Fibrillation** | | | |
| No | 323,602 (91.19%) | 258,233 (90.99 %) | 65,369 (91.98 %) |
| Yes | 31,259 (8.81%) | 25,556 (9.01 %) | 5,703 (8.02 %) |
| **Liver failure** | | | |
| No | 353,920 (99.73%) | 283,035 (99.73 %) | 70,885 (99.74 %) |
| Yes | 941 (0.27%) | 754 (0.27 %) | 187 (0.26 %) |
| **CKD** | | | |
| No | 307,379 (86.62%) | 237,873 (83.82 %) | 69,506 (97.80 %) |
| Yes | 47,482 (13.38%) | 45,916 (16.18 %) | 1,566 (2.20 %) |
| **Pulmonary Embolism** | | | |
| No | 346,544 (97.66%) | 277,125 (97.65 %) | 69,419 (97.67 %) |
| Yes | 8,317 (2.34%) | 6,664 (2.35 %) | 1,653 (2.33 %) |
| **Deep Vein Thrombosis** | | | |
| No | 338,582 (95.41%) | 272,228 (95.93 %) | 66,354 (93.36 %) |
| Yes | 16,279 (4.59%) | 11,561 (4.07 %) | 4,718 (6.64 %) |
| **Diabetes** | | | |
| No | 299,195 (84.31%) | 241,530 (85.11 %) | 57,665 (81.14 %) |
| Yes | 55,666 (15.69%) | 42,259 (14.89 %) | 13,407 (18.86 %) |
| **Osteoarthritis** | | | |
| No | 108,501 (30.58%) | 84,238 (29.68 %) | 24,263 (34.14 %) |
| Yes | 246,360 (69.42%) | 199,551 (70.32 %) | 46,809 (65.86 %) |
| **Rheumatoid Arthritis** | | | |
| No | 278,088 (78.37%) | 211,699 (74.60 %) | 66,389 (93.41 %) |
| Yes | 76,773 (21.63%) | 72,090 (25.40 %) | 4,683 (6.59 %) |
| **Active Cancer** | | | |
| No | 197,515 (55.66%) | 148,212 (52.23 %) | 49,303 (69.37 %) |
| Yes | 157,346 (44.34%) | 135,577 (47.77 %) | 21,769 (30.63 %) |
| **Heart Failure** | | | |
| No | 346,046 (97.52%) | 276,416 (97.40 %) | 69,630 (97.97 %) |
| Yes | 8,815 (2.48%) | 7,373 (2.60 %) | 1,442 (2.03 %) |
| **Myocardial Infarction** | | | |
| No | 324,086 (91.33%) | 256,242 (90.29 %) | 67,844 (95.46 %) |
| Yes | 30,775 (8.67%) | 27,547 (9.71 %) | 3,228 (4.54 %) |
| **Ischaemic Heart Disease** | | | |
| No | 317,497 (89.47%) | 252,597 (89.01 %) | 64,900 (91.32 %) |
| Yes | 37,364 (10.53%) | 31,192 (10.99 %) | 6,172 (8.68 %) |
| **Hypertension** | | | |
| No | 156,943 (44.23%) | 126,703 (44.65 %) | 30,240 (42.55 %) |
| Yes | 197,918 (55.77%) | 157,086 (55.35 %) | 40,832 (57.45 %) |
| **COPD** | | | |
| No | 328,762 (92.65%) | 262,628 (92.54 %) | 66,134 (93.05 %) |

For peer review only

| | | | |
|---|---|---|---|
| Yes | 26,099 (7.35%) | 21,161 (7.46 %) | 4,938 (6.95 %) |
| **Hemorrhagic stroke** | | | |
| No | 348,525 (98.21%) | 277,864 (97.91 %) | 70,661 (99.42 %) |
| Yes | 6,336 (1.79%) | 5,925 (2.09 %) | 411 (0.58 %) |
| **Ischemic stroke** | | | |
| No | 343,389 (96.77%) | 276,570 (97.46 %) | 66,819 (94.02 %) |
| Yes | 11,472 (3.23%) | 7,219 (2.54 %) | 4,253 (5.98 %) |
| **Dementia** | | | |
| No | 341,598 (96.26%) | 273,956 (96.54 %) | 67,642 (95.17 %) |
| Yes | 13,263 (3.74%) | 9,833 (3.46 %) | 3,430 (4.83 %) |
| **Thyroidism** | | | |
| No | 343,187 (96.71%) | 281,401 (99.16 %) | 61,786 (86.93 %) |
| Yes | 11,674 (3.29%) | 2,388 (0.84 %) | 9,286 (13.07 %) |
| **Anemia** | | | |
| No | 313,850 (88.44%) | 243,727 (85.88 %) | 70,123 (98.66 %) |
| Yes | 41,011 (11.56%) | 40,062 (14.12 %) | 949 (1.34 %) |
| **Osteoporosis** | | | |
| No | 324,753 (91.52%) | 259,856 (91.57 %) | 64,897 (91.31 %) |
| Yes | 30,108 (8.48%) | 23,933 (8.43 %) | 6,175 (8.69 %) |
| **PJI before in same joint** | | | |
| No | 350,707 (98.83%) | 280,664 (98.90 %) | 70,043 (98.55 %) |
| Yes | 4,154 (1.17%) | 3,125 (1.10 %) | 1,029 (1.45 %) |
| **PJI before in another joint (* not only hip or knee)** | | | |
| No | 280,664 (98.90%) | 279,402 (98.94 %) | 1,262 (90.01 %) |
| Yes | 3,125 (1.10%) | 2,985 (1.06 %) | 140 (9.99 %) |
| **Total number of comorbidities** | | | |
| 0-1 | 72,069 (20.31%) | 53,909 (19.00 %) | 18,160 (25.55 %) |
| 2-3 | 163,665 (46.12%) | 127,623 (44.97 %) | 36,042 (50.71 %) |
| 4-5 | 88,620 (24.97%) | 74,689 (26.32 %) | 13,931 (19.60 %) |
| 6-7 | 24,618 (6.94%) | 22,085 (7.78 %) | 2,533 (3.56 %) |
| ≥ 8 | 4,989 (1.41%) | 4,623 (1.63 %) | 366 (0.51 %) |

For peer review only

**Table S 6.** Treatment history of patients at the time of hip or knee implant surgery.

| Variable | All | AURUM | GOLD |
|---|---|---|---|
| **Steroids injection** | | | |
| No use | 318,988 (89.89%) | 259,053 (91.28 %) | 59,935 (84.33 %) |
| Use < 3 months | 1,956 (0.55%) | 1,368 (0.48 %) | 588 (0.83 %) |
| Use 3 - 6 months | 3,629 (1.02%) | 2,568 (0.90 %) | 1,061 (1.49 %) |
| Use > 6 months | 30,288 (8.54%) | 20,800 (7.33 %) | 9,488 (13.35 %) |
| **Chondroitin glucosamine** | | | |
| No use | 349,095 (98.38%) | 280,998 (99.02 %) | 68,097 (95.81 %) |
| Use < 3 months | 942 (0.27%) | 493 (0.17 %) | 449 (0.63 %) |
| Use 3 - 6 months | 261 (0.07%) | 135 (0.05 %) | 126 (0.18 %) |
| Use > 6 months | 4,563 (1.29%) | 2,163 (0.76 %) | 2,400 (3.38 %) |
| **NSAID** | | | |
| No use | 135,320 (38.13%) | 124,717 (43.95 %) | 10,603 (14.92 %) |
| Use < 3 months | 68,755 (19.38%) | 55,388 (19.52 %) | 13,367 (18.81 %) |
| Use 3 - 6 months | 19,650 (5.54%) | 15,509 (5.46 %) | 4,141 (5.83 %) |
| Use > 6 months | 131,136 (36.95%) | 88,175 (31.07 %) | 42,961 (60.45 %) |
| **Methotrexate** | | | |
| No use | 346,094 (97.53%) | 277,084 (97.64 %) | 69,010 (97.10 %) |
| Use < 3 months | 5,191 (1.46%) | 4,304 (1.52 %) | 887 (1.25 %) |
| Use 3 - 6 months | 307 (0.09%) | 236 (0.08 %) | 71 (0.10 %) |
| Use > 6 months | 3,269 (0.92%) | 2,165 (0.76 %) | 1,104 (1.55 %) |
| **DMRAD** | | | |
| No use | 348,288 (98.15%) | 279,519 (98.50 %) | 68,769 (96.76 %) |
| Use < 3 months | 2,906 (0.82%) | 2,311 (0.81 %) | 595 (0.84 %) |
| Use 3 - 6 months | 289 (0.08%) | 231 (0.08 %) | 58 (0.08 %) |
| Use > 6 months | 3,378 (0.95%) | 1,728 (0.61 %) | 1,650 (2.32 %) |
| **Antibacterial** | | | |
| No use | 115,375 (32.51%) | 83,025 (29.26 %) | 32,350 (45.52 %) |
| Use < 3 months | 56,438 (15.90%) | 54,748 (19.29 %) | 1,690 (2.38 %) |
| Use 3 - 6 months | 26,117 (7.36%) | 24,826 (8.75 %) | 1,291 (1.82 %) |
| Use > 6 months | 156,931 (44.22%) | 121,190 (42.70 %) | 35,741 (50.29 %) |
| **Antifungal** | | | |
| No use | 289,298 (81.52%) | 224,414 (79.08 %) | 64,884 (91.29 %) |
| Use < 3 months | 7,721 (2.18%) | 7,530 (2.65 %) | 191 (0.27 %) |
| Use 3 - 6 months | 4,526 (1.28%) | 4,388 (1.55 %) | 138 (0.19 %) |
| Use > 6 months | 53,316 (15.02%) | 47,457 (16.72 %) | 5,859 (8.24 %) |
| **Direct Oral Anticoagulant** | | | |
| No use | 348,809 (98.29%) | 278,363 (98.09 %) | 70,446 (99.12 %) |
| Use < 3 months | 4,073 (1.15%) | 3,725 (1.31 %) | 348 (0.49 %) |
| Use 3 - 6 months | 346 (0.10%) | 303 (0.11 %) | 43 (0.06 %) |
| Use > 6 months | 1,633 (0.46%) | 1,398 (0.49 %) | 235 (0.33 %) |
| **Heparin** | | | |
| No use | 350,082 (98.65%) | 279,872 (98.62 %) | 70,210 (98.79 %) |

For peer review only

| | | | |
|---|---|---|---|
| Use < 3 months | 610 (0.17%) | 587 (0.21 %) | 23 (0.03 %) |
| Use 3 - 6 months | 286 (0.08%) | 261 (0.09 %) | 25 (0.04 %) |
| Use > 6 months | 3,883 (1.09%) | 3,069 (1.08 %) | 814 (1.15 %) |
| **Warfarin** | | | |
| No use | 330,072 (93.01%) | 265,204 (93.45 %) | 64,868 (91.27 %) |
| Use < 3 months | 14,565 (4.10%) | 12,094 (4.26 %) | 2,471 (3.48 %) |
| Use 3 - 6 months | 1,324 (0.37%) | 1,081 (0.38 %) | 243 (0.34 %) |
| Use > 6 months | 8,900 (2.51%) | 5,410 (1.91 %) | 3,490 (4.91 %) |
| **Intra-articular injections (n)** | | | |
| 0 | 340,313 (95.90%) | 271,827 (95.78 %) | 68,486 (96.36 %) |
| 1 | 10,231 (2.88%) | 8,389 (2.96 %) | 1,842 (2.59 %) |
| 2 | 2,661 (0.75%) | 2,197 (0.77 %) | 464 (0.65 %) |
| 3 | 867 (0.24%) | 721 (0.25 %) | 146 (0.21 %) |
| 4 | 326 (0.09%) | 264 (0.09 %) | 62 (0.09 %) |
| 5 | 212 (0.06%) | 178 (0.06 %) | 34 (0.05 %) |
| > 5 | 251 (0.07%) | 213 (0.08 %) | 38 (0.05 %) |
| **Last intra-articular injection** | | | |
| No use | 340,313 (95.90%) | 271,827 (95.78 %) | 68,486 (96.36 %) |
| Use < 3 months | 517 (0.15%) | 434 (0.15 %) | 83 (0.12 %) |
| Use 3 - 6 months | 2,210 (0.62%) | 1,805 (0.64 %) | 405 (0.57 %) |
| Use > 6 months | 11,821 (3.33%) | 9,723 (3.43 %) | 2,098 (2.95 %) |
| **Intra-articular injection in other joints (n)** | | | |
| 0 | 313,586 (88.37%) | 249,945 (88.07 %) | 63,641 (89.54 %) |
| 1 | 378 (0.11%) | 294 (0.10 %) | 84 (0.12 %) |
| 2 | 4,793 (1.35%) | 3,901 (1.37 %) | 892 (1.26 %) |
| 3 | 11,820 (3.33%) | 9,606 (3.38 %) | 2,214 (3.12 %) |
| 4 | 8,010 (2.26%) | 6,541 (2.30 %) | 1,469 (2.07 %) |
| 5 | 2,572 (0.72%) | 2,090 (0.74 %) | 482 (0.68 %) |
| > 5 | 13,702 (3.86%) | 11,412 (4.02 %) | 2,290 (3.22 %) |
| **Last intra-articular injection in other joints** | | | |
| No use | 313,586 (88.37%) | 249,945 (88.07 %) | 63,641 (89.54 %) |
| Use < 3 months | 1,542 (0.43%) | 1,266 (0.45 %) | 276 (0.39 %) |
| Use 3 - 6 months | 4,892 (1.38%) | 4,031 (1.42 %) | 861 (1.21 %) |
| Use > 6 months | 34,841 (9.82%) | 28,547 (10.06 %) | 6,294 (8.86 %) |

For peer review only

**Table S 7.** Summary of fitting performance measurement for fitting results from CPRD AURUM database with different parametric models.

| **Parametric model** | **AIC** | **BIC** | **Loglik** |
|---|---|---|---|
| Exponential | 36653.68 | 37709.28 | -18226.84 |
| Gaussian | 38659.97 | 39726.12 | -19228.98 |
| Logistic | 38760.25 | 39826.41 | -19279.13 |
| Log-logistic | 35828.08 | 36894.24 | -17813.04 |
| Lognormal | 35816.08 | 36882.23 | -17807.04 |
| Weibull | 35831.92 | 36898.07 | -17814.96 |

**Table S 8**. Summary of fitting performance measurement for fitting results from CPRD GOLD database with different parametric models.

| **Parametric model** | **AIC** | **BIC** | **Loglik** |
|---|---|---|---|
| Exponential | 7674.76 | 8573.56 | -3739.38 |
| Gaussian | 8097.86 | 9005.83 | -3949.93 |
| Logistic | 8116.71 | 9024.68 | -3959.36 |
| Log-logistic | 7543.99 | 8451.96 | -3672.99 |
| Lognormal | 7543.11 | 8451.08 | -3672.55 |
| Weibull | 7544.66 | 8452.64 | -3673.33 |

For peer review only

**Table S 9.** Regression coefficients and standard error (SE) for log-normal risk equation of time to PJI derived from CPRD AURUM and CPRD GOLD.

| Variable | AURUM | GOLD | SE AURUM | SE GOLD | *P* value |
|---|---|---|---|---|---|
| Scale | 5.555 | 5.167 | 0.821 | 1.459 | 0.817 |
| Intercept | 22.473 | 21.060 | 0.118 | 0.251 | < 0.001 |
| Male | -0.818 | -1.192 | 0.380 | 0.882 | 0.697 |
| Age | | | | | |
| ≤ 45 | Reference | | | | |
| 46 – 55 | 1.181 | 0.501 | 0.353 | 0.844 | 0.457 |
| 56 – 65 | 1.356 | 1.275 | 0.356 | 0.850 | 0.930 |
| 66 – 75 | 1.770 | 1.530 | 0.365 | 0.868 | 0.799 |
| 76 – 85 | 1.935 | 1.708 | 0.454 | 1.113 | 0.850 |
| > 85 | 3.276 | 3.457 | 0.460 | 0.835 | 0.849 |
| BMI | | | | | |
| ≤ 20 | Reference | | | | |
| 20 - 25 | -0.113 | -0.277 | 0.448 | 0.826 | 0.861 |
| 25 - 30 | -0.247 | -0.103 | 0.452 | 0.836 | 0.880 |
| 30 - 35 | -0.651 | -0.337 | 0.460 | 0.857 | 0.747 |
| 35 - 50 | -1.005 | -0.607 | 0.930 | 2.048 | 0.860 |
| >50 | -1.234 | -1.644 | 0.442 | 0.963 | 0.699 |
| Unknown | -0.978 | -0.461 | 2.044 | 0.403 | 0.804 |
| Smoking status | | | | | |
| Non-smoker | Reference | | | | |
| Cigar smoker | 0.575 | NA | 0.263 | NA | NA |
| Current smoker | -0.203 | -0.407 | 0.123 | 0.246 | 0.458 |
| Ex-smoker | -0.281 | -0.186 | 1.412 | 2.250 | 0.971 |
| Heavy smoker | -0.394 | -1.170 | 1.160 | 1.245 | 0.648 |
| Light smoker | 0.926 | -1.785 | 1.189 | 0 | 0.023 |
| Moderate smoker | 0.620 | NA | 0.931 | NA | NA |
| Quitting | 0.145 | NA | 0.189 | NA | NA |
| Unknown | -0.211 | -0.386 | 1.313 | 1.893 | 0.939 |
| Passive smoker | NA | 4082.997 | NA | 1.250 | NA |
| Alcohol consumption | | | | | |
| Non-drinker | Reference | | | | |
| Ex drinker | -0.951 | -0.739 | 0.784 | 0 | 0.787 |
| Heavy drinker | -0.727 | 3882 | 0.490 | 0.439 | < 0.001 |
| Light drinker | -0.684 | 0.002 | 0.549 | 0.731 | 0.453 |
| Moderate drinker | -0.620 | 1.137 | 0.612 | 0.864 | 0.097 |
| Other | -0.137 | 1.559 | 0.479 | 0.379 | 0.005 |
| Social drinker | -0.814 | 0.801 | 1.103 | 1.748 | 0.435 |
| Unknown | -0.507 | -0.023 | 0.111 | 0.228 | 0.056 |
| Very heavy drinker | -0.855 | 2.056 | 0.823 | 1.381 | 0.070 |
| Right side of operation | 0.165 | 0.498 | 0.233 | 0.486 | 0.537 |
| Type of fixation | | | | | |

For peer review only

| | | | | | |
|---|---|---|---|---|---|
| Cemented | Reference | | | | |
| Hybrid | 0.287 | -0.693 | 0.15 | 0.36 | 0.012 |
| Non cemented | 0.251 | 0.252 | 0.259 | 0.513 | 0.999 |
| Unknown | -0.06 | 0.007 | 0.164 | 0.343 | 0.860 |
| Primary arthroplasty | | | | | |
| Yes | Reference | | | | |
| No | -2.795 | -2.231 | 0.663 | 2.033 | 0.792 |
| unknown | -2.848 | -0.797 | 0.186 | 0.419 | < 0.001 |
| Admission type | | | | | |
| Elective | Reference | | | | |
| A&E | -0.991 | -1.177 | 0.831 | 1.905 | 0.929 |
| Unknown | -0.840 | 0.174 | 0.269 | 0.502 | 0.075 |
| Patella resurfacing | | | | | |
| No | NA | NA | NA | NA | NA |
| Yes | 0.737 | 0.284 | 0.138 | 0.312 | 0.184 |
| N/A | 0.250 | 1.480 | 0.542 | 0 | 0.023 |
| Graft | | | | | |
| No | Reference | | | | |
| Autograft | -0.528 | 133.113 | 2.322 | 2.707 | < 0.001 |
| Autograft + other | 0.182 | -6.800 | 0.453 | 1.313 | < 0.001 |
| Other graft | 0.285 | 1.136 | 0.237 | 0.511 | 0.131 |
| Atrial Fibrillation | -0.409 | -0.354 | 0.166 | 0.82 | 0.948 |
| CKD | 0.047 | -0.120 | 0.957 | 2.01 | 0.940 |
| Liver failure | -0.066 | -0.326 | 0.336 | 0.679 | 0.731 |
| Pulmonary Embolism | -0.186 | -0.728 | 0.254 | 0.478 | 0.317 |
| Deep Vein Thrombosis | -0.288 | 0.396 | 0.149 | 0.29 | 0.036 |
| Diabetes | -0.289 | 0.152 | 0.131 | 0.251 | 0.119 |
| Osteoarthritis | -0.210 | -0.052 | 0.129 | 0.454 | 0.738 |
| Rheumatoid Arthritis | -0.098 | -0.521 | 0.119 | 0.249 | 0.125 |
| Active Cancer | 0.154 | -0.313 | 0.370 | 1.065 | 0.679 |
| Heart Failure | 0.324 | 1.120 | 0.290 | 0.663 | 0.271 |
| Myocardial Infarction | -0.433 | 0.700 | 0.278 | 0.453 | 0.033 |
| Ischaemic Heart Disease | 0.085 | 0.080 | 0.118 | 0.241 | 0.985 |
| Hypertension | -0.281 | -0.439 | 0.213 | 0.480 | 0.764 |
| COPD | -0.025 | 0.295 | 0.495 | 1.680 | 0.855 |
| Haemorrhagic Stroke | -0.248 | 0.097 | 0.467 | 0.496 | 0.613 |
| Ischaemic Stroke | 0.273 | -0.131 | 0.443 | 0.667 | 0.614 |
| Dementia | 0.439 | -0.026 | 0.637 | 0.335 | 0.518 |
| Thyroidism | 0.032 | -0.462 | 0.161 | 1.276 | 0.701 |
| Anaemia | 0.025 | 1.274 | 0.236 | 0.521 | 0.029 |
| Osteoporosis | 0.366 | 0.605 | 0.329 | 0.794 | 0.781 |
| PJI before in same joint | -2.656 | -1.291 | 0.288 | 0.587 | 0.037 |
| PJI before in another joint | -1.874 | -1.502 | 0.696 | 1.088 | 0.773 |

For peer review only

| | | | | | |
|---|---|---|---|---|---|
| Steroids injection | | | | | |
| No use | Reference | | | | |
| Use ≤ 3 months | -0.479 | -0.263 | 0.506 | 0.925 | 0.838 |
| Use 3 - 6 months | -0.677 | 0.415 | 0.198 | 0.312 | 0.003 |
| Use > 6 months | -0.429 | -0.140 | 1.452 | 1.736 | 0.898 |
| Chondroitin Glucosamine | | | | | |
| No use | Reference | | | | |
| Use ≤ 3 months | 0.848 | 1.524 | 1.963 | 0 | 0.731 |
| Use 3 - 6 months | -0.981 | 2414.803 | 0.554 | 0.613 | < 0.001 |
| Use > 6 months | -0.446 | 0.357 | 0.144 | 0.449 | 0.089 |
| NSAID | | | | | |
| No use | Reference | | | | |
| Use ≤ 3 months | -0.807 | -1.673 | 0.239 | 0.549 | 0.148 |
| Use 3 - 6 months | -0.22 | -1.505 | 0.145 | 0.415 | 0.003 |
| Use > 6 months | 0.341 | -0.283 | 0.391 | 1.008 | 0.564 |
| Methotrexate | | | | | |
| No use | Reference | | | | |
| Use ≤ 3 months | -0.764 | 0.666 | 2.213 | 0 | 0.518 |
| Use 3 - 6 months | 1.392 | 276.007 | 0.640 | 0.771 | < 0.001 |
| Use > 6 months | 0.125 | -0.920 | 0.771 | 0.845 | 0.361 |
| DMRAD | | | | | |
| No use | Reference | | | | |
| Use ≤ 3 months | 1.598 | -1.814 | 1.295 | 0 | 0.008 |
| Use 3 - 6 months | -1.471 | 2815.254 | 0.618 | 0.801 | < 0.001 |
| Use > 6 months | -0.535 | 0.741 | 0.168 | 0.720 | 0.084 |
| Antibacterial | | | | | |
| No use | Reference | | | | |
| Use ≤ 3 months | -0.177 | -0.160 | 0.208 | 0.625 | 0.979 |
| Use 3 - 6 months | -0.252 | -1.647 | 0.142 | 0.236 | < 0.001 |
| Use > 6 months | -0.042 | -0.199 | 0.296 | 1.853 | 0.933 |
| Antifungal | | | | | |
| No use | Reference | | | | |
| Use ≤ 3 months | -0.677 | -0.700 | 0.409 | 0 | 0.955 |
| Use 3 - 6 months | -0.289 | 76.029 | 0.147 | 0.335 | < 0.001 |
| Use > 6 months | -0.300 | -0.999 | 0.436 | 1.735 | 0.696 |
| Direct Oral Anticoagulant | | | | | |
| No use | Reference | | | | |
| Use ≤ 3 months | -1.185 | -0.069 | 20231.550 | 0 | 1 |
| Use 3 - 6 months | 28.326 | 1840.160 | 0.728 | 0 | < 0.001 |
| Use > 6 months | -0.465 | 1267.094 | 1 | 0 | < 0.001 |
| Heparin | | | | | |
| No use | Reference | | | | |
| Use ≤ 3 months | -0.237 | 61.390 | 1.220 | 0 | < 0.001 |
| Use 3 - 6 months | -1.326 | 53.522 | 0.535 | 0.926 | < 0.001 |
| Use > 6 months | 0.284 | -0.501 | 0.308 | 0.645 | 0.272 |
| Warfarin | | | | | |

For peer review only

| | | | | | |
|---|---|---|---|---|---|
| No use | Reference | | | | |
| Use ≤ 3 months | -0.228 | -1.410 | 0.804 | 1.514 | 0.490 |
| Use 3 - 6 months | 0.046 | -1.029 | 0.383 | 0.579 | 0.121 |
| Use > 6 months | -0.187 | -0.179 | 1.629 | 0 | 0.996 |
| Intra-articular injection | | | | | |
| No use | Reference | | | | |
| Use ≤ 3 months | 1.57 | 33.164 | 0.912 | 0 | < 0.001 |
| Use 3 - 6 months | -1.57 | 175.201 | 0.336 | 0.780 | < 0.001 |
| Use > 6 months | -0.099 | -0.758 | 0.771 | 2.005 | 0.759 |
| Intra-articular injections in other joints | | | | | |
| No use | Reference | | | | |
| Use ≤ 3 months | -0.875 | 0.246 | 0.685 | 1.268 | 0.437 |
| Use 3 - 6 months | 0.753 | -0.158 | 0.202 | 0.477 | 0.079 |
| Use > 6 months | -0.203 | 0.449 | 0.024 | 0.052 | < 0.001 |

For peer review only

**Table S 10.** C- statistic values for pooled risk equation at different follow-up.

| Follow-up | Mean | 95% CI |
|---|---|---|
| 1 year | 0.735 | 0.719 - 0.751 |
| 2 years | 0.730 | 0.716 - 0.744 |
| 3 years | 0.728 | 0.711 - 0.742 |
| 4 years | 0.726 | 0.713 - 0.740 |
| 5 years | 0.724 | 0.711 - 0.737 |

For peer review only

TRIPOD

# TRIPOD Checklist: Prediction Model Development and Validation

| Section/Topic | Item | | Checklist Item | Page |
|---|---|---|---|---|
| **Title and abstract** | | | | |
| Title | 1 | D;V | Identify the study as developing and/or validating a multivariable prediction model, the target population, and the outcome to be predicted. | 1 |
| Abstract | 2 | D;V | Provide a summary of objectives, study design, setting, participants, sample size, predictors, outcome, statistical analysis, results, and conclusions. | 2 |
| **Introduction** | | | | |
| Background and objectives | 3a | D;V | Explain the medical context (including whether diagnostic or prognostic) and rationale for developing or validating the multivariable prediction model, including references to existing models. | 4 |
| | 3b | D;V | Specify the objectives, including whether the study describes the development or validation of the model or both. | 4-5 |
| **Methods** | | | | |
| Source of data | 4a | D;V | Describe the study design or source of data (e.g., randomized trial, cohort, or registry data), separately for the development and validation data sets, if applicable. | 5 |
| | 4b | D;V | Specify the key study dates, including start of accrual; end of accrual; and, if applicable, end of follow-up. | 6 |
| Participants | 5a | D;V | Specify key elements of the study setting (e.g., primary care, secondary care, general population) including number and location of centres. | 5-6 |
| | 5b | D;V | Describe eligibility criteria for participants. | 6 |
| | 5c | D;V | Give details of treatments received, if relevant. | 6 |
| Outcome | 6a | D;V | Clearly define the outcome that is predicted by the prediction model, including how and when assessed. | 7 |
| | 6b | D;V | Report any actions to blind assessment of the outcome to be predicted. | NA |
| Predictors | 7a | D;V | Clearly define all predictors used in developing or validating the multivariable prediction model, including how and when they were measured. | 6-7 |
| | 7b | D;V | Report any actions to blind assessment of predictors for the outcome and other predictors. | NA |
| Sample size | 8 | D;V | Explain how the study size was arrived at. | 9 |
| Missing data | 9 | D;V | Describe how missing data were handled (e.g., complete-case analysis, single imputation, multiple imputation) with details of any imputation method. | 7 |
| Statistical analysis methods | 10a | D | Describe how predictors were handled in the analyses. | 8 |
| | 10b | D | Specify type of model, all model-building procedures (including any predictor selection), and method for internal validation. | 7-8 |
| | 10c | V | For validation, describe how the predictions were calculated. | 7-8 |
| | 10d | D;V | Specify all measures used to assess model performance and, if relevant, to compare multiple models. | 8 |
| | 10e | V | Describe any model updating (e.g., recalibration) arising from the validation, if done. | NA |
| Risk groups | 11 | D;V | Provide details on how risk groups were created, if done. | NA |
| Development vs. validation | 12 | V | For validation, identify any differences from the development data in setting, eligibility criteria, outcome, and predictors. | 8 |
| **Results** | | | | |
| Participants | 13a | D;V | Describe the flow of participants through the study, including the number of participants with and without the outcome and, if applicable, a summary of the follow-up time. A diagram may be helpful. | 9 |
| | 13b | D;V | Describe the characteristics of the participants (basic demographics, clinical features, available predictors), including the number of participants with missing data for predictors and outcome. | 9-10 |
| | 13c | V | For validation, show a comparison with the development data of the distribution of important variables (demographics, predictors and outcome). | NA |
| Model development | 14a | D | Specify the number of participants and outcome events in each analysis. | 10 |
| | 14b | D | If done, report the unadjusted association between each candidate predictor and outcome. | NA |
| Model specification | 15a | D | Present the full prediction model to allow predictions for individuals (i.e., all regression coefficients, and model intercept or baseline survival at a given time point). | Table 1 |
| | 15b | D | Explain how to the use the prediction model. | 9-10 |
| Model performance | 16 | D;V | Report performance measures (with CIs) for the prediction model. | 10-11 |
| Model-updating | 17 | V | If done, report the results from any model updating (i.e., model specification, model performance). | NA |
| **Discussion** | | | | |
| Limitations | 18 | D;V | Discuss any limitations of the study (such as nonrepresentative sample, few events per predictor, missing data). | 13 |
| Interpretation | 19a | V | For validation, discuss the results with reference to performance in the development data, and any other validation data. | 12 |
| | 19b | D;V | Give an overall interpretation of the results, considering objectives, limitations, results from similar studies, and other relevant evidence. | 11-13 |
| Implications | 20 | D;V | Discuss the potential clinical use of the model and implications for future research. | 11-14 |
| **Other information** | | | | |
| Supplementary information | 21 | D;V | Provide information about the availability of supplementary resources, such as study protocol, Web calculator, and data sets. | 14 |
| Funding | 22 | D;V | Give the source of funding and the role of the funders for the present study. | 14 |

*Items relevant only to the development of a prediction model are denoted by D, items relating solely to a validation of a prediction model are denoted by V, and items relating to both are denoted D;V. We recommend using the TRIPOD Checklist in conjunction with the TRIPOD Explanation and Elaboration document.

For peer review only - http://bmjopen.bmj.com/site/about/guidelines.xhtml

BMJ Open

# Retrospective study in UK using the Clinical Practice Research Datalink (CPRD) AURUM and GOLD database for the determination of risk equations for prosthetic joint infections (PJI)



| | |
|---|---|
| Journal: | *BMJ Open* |
| Manuscript ID | bmjopen-2023-082501.R1 |
| Article Type: | Original research |
| Date Submitted by the Author: | 21-Mar-2024 |
| Complete List of Authors: | Perni, Stefano; Cardiff University<br>Prokopovich, Polina; Cardiff University |
| <b>Primary Subject Heading</b>: | Infectious diseases |
| Secondary Subject Heading: | Qualitative research, Surgery, Epidemiology, Medical management |
| Keywords: | Epidemiology < INFECTIOUS DISEASES, Hip < ORTHOPAEDIC & TRAUMA SURGERY, Electronic Health Records, Knee < ORTHOPAEDIC & TRAUMA SURGERY |
| | |



SCHOLARONE™
Manuscripts

*I, the Submitting Author has the right to grant and does grant on behalf of all authors of the Work (as defined in the below author licence), an exclusive licence and/or a non-exclusive licence for contributions from authors who are: i) UK Crown employees; ii) where BMJ has agreed a CC-BY licence shall apply, and/or iii) in accordance with the terms applicable for US Federal Government officers or employees acting as part of their official duties; on a worldwide, perpetual, irrevocable, royalty-free basis to BMJ Publishing Group Ltd ("BMJ") its licensees and where the relevant Journal is co-owned by BMJ to the co-owners of the Journal, to publish the Work in this journal and any other BMJ products and to exploit all rights, as set out in our licence.*

*The Submitting Author accepts and understands that any supply made under these terms is made by BMJ to the Submitting Author unless you are acting as an employee on behalf of your employer or a postgraduate student of an affiliated institution which is paying any applicable article publishing charge ("APC") for Open Access articles. Where the Submitting Author wishes to make the Work available on an Open Access basis (and intends to pay the relevant APC), the terms of reuse of such Open Access shall be governed by a Creative Commons licence – details of these licences and which Creative Commons licence will apply to this Work are set out in our licence referred to above.*

*Other than as permitted in any relevant BMJ Author's Self Archiving Policies, I confirm this Work has not been accepted for publication elsewhere, is not being considered for publication elsewhere and does not duplicate material already published. I confirm all authors consent to publication of this Work and authorise the granting of this licence.*

For peer review only

# Retrospective study in UK using the Clinical Practice Research Datalink (CPRD) AURUM and GOLD database for the determination of risk equations for prosthetic joint infections (PJI)

For peer review only

*Stefano Perni[1] and Polina Prokopovich[1,*]*

[1] School of Pharmacy and Pharmaceutical Sciences, Cardiff University, Cardiff, UK

WORD COUNT: 3948

**Corresponding author: Dr Polina Prokopovich**

School of Pharmacy and Pharmaceutical Sciences

Cardiff University

Redwood Building, King Edward VII Avenue

Cardiff, UK

CF10 3NB

E-mail address: prokopovichp@cf.ac.uk



For peer review only - http://bmjopen.bmj.com/site/about/guidelines.xhtml

# 1 Abstract

**Background:** Prosthetic joint infections (PJI) are a serious negative outcome of arthroplasty with incidence of about 1%. Risk of PJI could depend on local treatment policies and guidelines; no UK specific risk scoring is currently available.

**Objective:** to use electronic health records to determine a risk quantification model for the development of PJI

**Design:** Records in Clinical Practice Research Datalink (CPRD) GOLD and AURUM of patients undergoing hip or knee arthroplasty between Jan 2007 and Dec 2015, with linkage to Hospital episode statistic (HES) and office of national statistic (ONS), were obtained. Cohorts' characteristics and risk equations through parametric models were developed and compared between the two databases. Pooled cohort risk equations were determined for the UK population and simplified through stepwise selection.

**Results:** After applying the inclusion/exclusion criteria, 174,905 joint (1,021 developed PJI) were identified in CPRD AURUM and 48,419 joint (228 developed PJI) in CPRD GOLD. Patients undergoing hip or knee arthroplasty in both databases exhibited different sociodemographic characteristics and medical/drug history. However, the quantification of the impact of such covariates (coefficients of parametric models fitted to the survival curves) in the risk of PJI were not statistically different between the two cohorts. The log-normal model fitted to the pooled cohorts after stepwise selection had a C-statistic > 0.7.

**Conclusions:** the risk prediction tool developed here could help preventing PJI through identifying modifiable risk factors pre-surgery and identifying the patients most likely to benefit from close monitoring/preventive actions. As derived from the UK population, such tool will help the NHS reducing the impact of PJI on its resources and patient lives.

**Keywords:** PJI, CPRD, pooled cohort equations, risk prediction



For peer review only - http://bmjopen.bmj.com/site/about/guidelines.xhtml

# 2 List of abbreviations

| | |
|---|---|
| AC | Active cancer |
| A&E | Accident and Emergency |
| AF | Atrial Fibrillation |
| AIC | Akaike Information Criterion |
| BIC | Bayesian Information Criterion |
| BMI | Body mass index |
| CKD | Chronic kidney disease |
| COPD | Chronic obstructive pulmonary disease |
| CPRD | Clinical Practice Research Datalink |
| DMRAD | Disease modifying rheumatoid arthritis drug |
| DOAC | Direct oral anticoagulant |
| DVT | Deep vein thrombosis |
| HES | Hospital episode statistic |
| HF | Heart failure |
| HTA | Health technology assessment |
| IHD | Ischaemic heart disease |
| MI | Myocardial infarction |
| MTX | Methotrexate |
| NHS | National health service |
| NICE | National Institute for Health and Care Excellence |
| NSAID | Non-steroidal anti-inflammatory drug |
| PE | Pulmonary embolism |
| PJI | Prostatic joint infection |
| OA | Osteoarthritis |
| ONS | Office national statistic |
| RA | Rheumatoid arthritis |

For peer review only



For peer review only - http://bmjopen.bmj.com/site/about/guidelines.xhtml

# 3 Strengths and Limitations

- We sampled a large cohort of patients undergoing hip or knee replacement and considered a wide-range of covariates and of drugs to establish the medical and medication history. The specific geographical location (UK) may limit the availability of data regarding the impact of ethnicity and not have the same predictive power when applied to other countries.
- It is plausible some confounding variables are unavailable, either because they are not routinely recorded (for example, nutritional status and genetic factors) or unavailable (for example, hospital prescribing and use of over-the-counter medications).
- The use of registry-based data incurs in the possibility of error in the recording of data or codes may be a source of information bias.
- As the CPRD is a retrospective observational database, clinical measurements are collected through routine primary care rather than being mandated through a study protocol, as in a prospective study, leading to missing data. Study population is restricted to patients with complete data at baseline for certain variables. This may invite bias, as the patient profile of those with complete data may be different to those without. However, a workable dataset is required to fulfil the study objectives and, therefore, this practical approach will be adopted to identify a suitable study population. Comorbidities were considered as binary (present or absent), without accounting for illness severity and only at baseline  without accounting for time-varying factors.
- Our study was observational thus only able to identify correlations between PJI and the included covariates. Additional studies employed a casual inference would be required to establish causal factors.

For peer review only



For peer review only - http://bmjopen.bmj.com/site/about/guidelines.xhtml

# 4 Introduction

Infections associated to arthroplasty, known as prosthetic joint infections (PJIs), have an incidence of about 1% after primary arthroplasty, this is about 5 times higher after secondary arthroplasty not resulting from a previous PJI (aseptic replacement) while about 10 time after a revision caused by a PJI.[1, 2] The consequences of developing a PJI can be extremely debilitating long-term for patients [3] and, with an estimate mean cost to the NHS of ~£30,000 per PJI in knee joints [4] and ~£24,000 for PJI in hips, also health care providers incur high costs.

Numerous risk factors for PJI have been identified and risk quantification tools developed [5-9] as the possibility of identifying patients at risks for PJI would allow a more proactive and targeted approach to prevention; however, the quantification of an individual probability to develop PJI is often impacted by the size of the cohort used in deriving such risk equations and the covariates available in the datasets employed for such analysis.[10, 11] Such limitations reduce the ability to translate conclusions to other settings/countries and limit the accuracy of the estimated risk for patients.

Moreover, UK specific risk and contemporary equations have not been developed yet and such PJI risk estimates are calculated based on results from different countries (such as Sweden [5]) where treatment algorithms and local medical guidelines may differ from UK.

Clinical Practice Research Datalink (CPRD) is a UK government not-for-profit research service; it collects health records from participating GP practices that have agreed to contribute data to this database related to diagnoses, symptoms, prescriptions, referrals and tests for patients. It has provided anonymized primary care data to conduct public health research.[12] CPRD GOLD and AURUM database have been shown to be a data source with quality and completeness [13]. CPRD also provide linkage to Office of National statistics (ONS), providing accurate date and cause of death, along with Hospital episode statistics



For peer review only - http://bmjopen.bmj.com/site/about/guidelines.xhtml

94 (HES) contains records of secondary care activities including dates, specialty, clinical diagnoses and procedures.[12] Due to some differences in the structure and clinical coding of IT systems employed by the GP practices,[14] CPRD data are provided as separate databases: CPRD GOLD contains data contributed by practices using Vision® software, [15] while CPRD AURUM contains data from practices using EMIS® Web.[12] In September 2018, CPRD AURUM included records of 19 million patients registered in 738 practices (~ 10% of all English practices), of whom 7 million were alive and actively contributing (13% of the population of England). In 2013, CPRD GOLD contained records from 11 million patients registered in 674 practices; including 4.4 million patients active (alive and currently registered), approximately 6.9% of the UK population.[15] Therefore, they both offer a representative population of UK patients; however when linkage to HES and ONS is employed, only patients registered in GP practices located in England are represented in the final dataset.

Because of the lack of contemporary and UK specific rick equations for the development of PJI, the objectives of this study were multiples. Firstly, we wanted to compare the characteristics of patients developing PJI in both CPRD databases, then to estimate risk equations from each database and compare the contribution of each individual covariate in both databases to assess the possibility of pooling participants. Finally, relevant risk factors were identified from a stepwise regression of the pooled cohorts risk equation and the predictive ability of the newly developed risk equation assessed.



For peer review only - http://bmjopen.bmj.com/site/about/guidelines.xhtml

# 5 Methods

## 5.1 Data sources

Data were obtained from both databases available in the CPRD; Furthermore, CPRD data were linked to HES secondary care data and ONS mortality data.

This study protocol (19_009) was reviewed by the Independent Scientific Advisory Committee (ISAC) and approved in January 2020; a minor amendment was subsequently approved on 1st December 2021. Data were extracted in December 2020. The research team had access to only de-identified data therefore the requirement for patient consent was waived.

## 5.2 Study design and population

This was a retrospective study of patients undergoing hip or knee arthroplasty; the index date for inclusion was the day of arthroplasty. Patients had to be aged ≥ 30 years at index date and have arthroplasty between 01/01/2007 and 31/12/2014. Patients were excluded from the study if they were registered on the CPRD for < 6 months prior to index date with the most recent CPRD up-to-standard (UTS) date > 6 months prior to index date.

This research is largely descriptive rather than inferential in nature in that it aims to characterize patterns of PJI and verify the generalizability of existing risk equations. A sample size and power calculation have, therefore, not been undertaken with respect to identifying differences in outcomes between groups.

Eligible patients were identified through OPCS Classification of Interventions and Procedures (OPCS-4) codes [2] for hip or knee arthroplasty in HES records and they were followed-up for 5 years or the first occurrence of: device replaced, death or loss to follow-up. Loss to follow-up was be defined as the date a patient was transferred out of the practice, the date that the practice left the database, or the last recorded event date.



For peer review only - http://bmjopen.bmj.com/site/about/guidelines.xhtml

## 5.3 Study measures

Covariate related to patient characteristics were extracted from the CPRD database; surgery properties were derived from the HES database according to OPCS codes (for type of fixation, laterality fixation, grafts ...) and admission codes. Medical history was determined by the presence before index date of disease specific ICD-10 codes in HES or either MedCodeID codes in the CPRD AURUM database or READ codes in CPRD GOLD database. Prescription history was assessed from relevant BNF codes reported in the CPRD database.[2] Data cleaning was performed to remove implausible entries such as day of death before index date, and body mass index (BMI) < 10 or BMI > 80.

PJI occurrence was assess by the presence of the ICD-10 code T84.5 in the HES database; the date of PJI occurrence was set as the day of hospitalization. To ascertain that PJI diagnosed were affecting the joint of interest as patients could have multiple devices, only a diagnosis of PJI and a record to OPCS any procedure in the joint of interest during the hospitalization were considered.

## 5.4 Missing data handling

Medical history was based on the presence of a specific diagnostic code, therefore the absence of such codes in a patient records was assumed to represent absence of such event, therefore no missing data were possible in a patient medical history. On the contrary, when a record was expected, such in case of BMI, age, primary or secondary arthroplasty along with alcohol and smoking status, the absence of any entry resulted in this patient having the covariate under consideration categorized as missing. Missing data were handled through categorical binning and no imputation methods applied.



For peer review only - http://bmjopen.bmj.com/site/about/guidelines.xhtml

## 5.5 Survival analysis

Survival curves for each database were fitted with parametric models (Exponential, Weibull, normal, log-normal, logistic, log-logistic) and ranked according to Akaike information criteria (AIC) and Bayesian information criteria (BIC).

Stepwise regression was employed to reduce the number of covariates in the risk equation using AIC as criteria to identify which variable to add/remove or to stop the selection process.

## 5.6 Statistical analysis

Descriptive analyses were generated, characterizing patient demographics, clinical and treatment characteristics. Summary statistics (e.g. mean, standard deviation, standard error, median, inter-quartile range, minimum, and maximum) were calculated for continuous variables (age and BMI) while number and proportion/percentage were calculated for categorical variables. For multivariate regression, age was categorised as ≤ 45, 46 – 55, 56 – 65, 66 – 75, 76 – 85 and > 85 while BMI was categorised as ≤ 20, 20 – 25, 26 – 30, 31 – 35, 36 – 50 and > 50. The number and proportion of patients with missing data are also determined for each of the variables of interest.

Kaplan-Meier curves were generated, and the log-rank test was used to compare survival curves between the CPRD AURUM and CPRD GOLD databases.

PJI rates were calculated dividing the number of PJI cases observed by the total duration of the follow-up.

Z-test for each factor was performed to compare of the regression coefficients between risk equations developed using CPRD AURUM and CPRD GOLD.[16]

184 $$Z_i = \frac{\beta_{i,AURUM} - \beta_{i,GOLD}}{\sqrt{SE^2_{i,AURUM} + SE^2_{i,GOLD}}}$$

Where:



For peer review only - http://bmjopen.bmj.com/site/about/guidelines.xhtml

$\beta_i$ = regression coefficient for factor *i*

$SE_i$ = Standard error of regression coefficient for factor *i*

The predictive ability of the final risk equation was assess calculating the area under the precision-recall and receiver-operator curves at time-varying follow-ups. C-statistic was evaluated after different follow-up; 95% confidence intervals were estimated through bootstrapping with 500 replicates for each timepoint.

All data collection, analysis and visualization were performed using R (ver 4.2.2) and relevant packages.[17, 18]

## 5.7 *Patient and public involvement*

Patients and/or the public were not involved in the design, or conduct, or reporting, or dissemination plans of this research.

For peer review only



For peer review only - http://bmjopen.bmj.com/site/about/guidelines.xhtml

# 6 Results

## 6.1 Patient counts

After linkage with both HES and ONS, records for 330,173 hip of knee arthroplasty, corresponding to 235,249 patients were identified in CPRD AURUM; similarly, CPRD GOLD contained information for 88,458 hip or knee joint subjected to arthroplasty related to 62,672 patients. After applying the inclusion/exclusion criteria the records of 174,905 joint (144,048 patients) were identified in CPRD AURUM and 48,419 joint (39,764 patients) in CPRD GOLD (Table S1).

In patients indexed in either CPRD AURUM or GOLD, the most common reason for not completing the study observation period (5 years) was death, 14.67% and 12.44 % in CPRD AURUM and GOLD, respectively. The second most common reason in CPRD AURUM was transfer out of the CPRD database (in 12.91% of cases) while in CPRD GOLD it was data collection terminated before end-of study (in 34.29 % of cases); replacement of the implanted device in the first 5 years after surgery occurred in about 2.3 % of cases in both databases. A diagnosis of PJI in the first years after implant occurred in 1,021 joint (0.58 %) in CPRD AURUM and 228 (0.47 %) joints in CPRD GOLD (Table S2).

Patients not completing the study observation period were follow-up for a median of 726 and 768 days in CPRD AURUM and GOLD, respectively (Table S3).

## 6.2 Baseline characteristics

The demographic and basic characteristics of patients in both CPRD databases were generally similar for all covariates tested (e.g. gender, age, BMI, alcohol and smoking status) (Table S4); the level on unknown BMI was greater in AURUM than GOLD. The characteristics of the arthroplasty exhibited similar distribution in both databases (Table S5).



For peer review only - http://bmjopen.bmj.com/site/about/guidelines.xhtml

Patients undergoing hip or knee arthroplasty had similar prevalence of most of the diseases included in this study, however chronic kidney disease (CKD), rheumatoid arthritis (RA), myocardial infarction (MI), anaemia and cancer had different distributions in AURUM and GOLD (Table S6). Moreover, the also history of prescriptions in patients with hip and knee arthroplasty in CPRD AURUM and GOLD were comparable (Table S7).

## 6.3 PJI occurrence

Unadjusted Kaplan-Meier curve for time to PJI diagnosis showed a decreasing hazard rate with time; 0.21% and 0.32% of joints in CPRD AURUM developed PJI in the first 6 and 12 months, respectively. After 2 and 4 years the proportion of joints with PJI was 0.45% and 0.61% respectively. The probability of developing PJI in the joints from CPRD GOLD was 0.15%, 0.25%, 0.42% and 0.56% after 6 months, 1 year, 2 years and 4 years respectively. The log-rank test between the two curves had $P < 0.001$ (Figure 1a).

Likewise, the Kaplan-Meier curves for time to PJI in the two CPRD databases exhibited comparable patterns with $P$ values generally < 0.001 (Figure 1b and c).

The distribution of the PJI rate different across the different England regions defined in CPRD ranging from ~100 PJI cases/100,000 patients year in the East Midlands to ~180 PJI cases/100,000 patients year in the North East (Figure 1d).

## 6.4 Risk equations

Survival curves of time to PJI diagnosis in CPRD GOLD and CPRD AURUM were fitted independently with parametric models; for both databases the log-normal distribution returned the best fitting (e.g. lowest AIC, lowest BIC and highest loglik) (Table S8 and Table S9). The coefficients of the log-normal parametric models for both databases were compared with Z-test and generally $P > 0.05$ (Figure 2 and Table S10).



For peer review only - http://bmjopen.bmj.com/site/about/guidelines.xhtml

The risk equations based on the log-normal survival model developed using the pooled cohorts had performance in categorizing a patients as at risk of PJI or not over different follow-up times increasing with longer periods as revealed by the area under the precision-recall curve; the area under the receiver operator curve (AUC) and C-statistic did not exhibit the same pattern of increasing performance with longer follow-up instead (Figure 3 and Table S11).

The stepwise variable selection was employed to reduce the number of covariates in the risk equation to those most significant. The final list of coefficients revealed that gender, age, BMI were patient characteristics that represent risk factors for PJI. The type of surgery (primary or secondary), admission through accident and emergency (A&E) or elective and the resurfacing of the patella were the risk factors related to the surgical procedure. A history of atrial fibrillation (AF), deep vein thrombosis (DVT), diabetes, osteoarthritis (OA), MI and hypertension were all increasing the risk of PJI. Previous PJIs, even in an unrelated joint, were also further medical risk factors for a further PJI. The use of non-steroidal anti-inflammatory drugs (NSAID) and antifungal drugs before arthroplasty also were risk factors (Table 1). Examples of the fitting of the Kaplan-Meier curves with the log-normal stepwise regression model showed a general good agreement (Figure 4).

# 7 Discussion

PJI is a relative rare but highly impactful outcome, both in terms of patients' quality of life and resources consumption,[3, 4, 19-22] of arthroplasty. Prevention or early identification of PJI through stringent medical follow-up is not a viable option because of the large population undergoing hip and knee arthroplasty and the potential long follow-up time, as shown here and in multiple studies PJI can be diagnosed even years after surgery.[2, 23, 24] Because of



For peer review only - http://bmjopen.bmj.com/site/about/guidelines.xhtml

the long follow-up required to capture the full extent of PJI, we chose a study period of 5 years and to allow patients to achieve such observation we restricted the last date of index surgery to 5 years before the last data collection in CPRD extraction analysed. In light of the administrative nature of the CPRD database, medical history was based on the presence of a specific diagnostic code, therefore the absence of such code in a patient records was assumed to represent absence of such event. On the contrary when a record was expected, such in case of BMI, primary or secondary arthroplasty along with alcohol and smoking status, the absence of any entry resulted in this patient having the covariate under consideration categorized as "unknown".

Patients identified in CPRD AURUM were statistically different than those identified in CPRD GOLD (Table S4-S7) likely as a consequence of the different geographical coverage of England by the two databases associated to the population characteristics variation around the country in term of age and diseases.[25, 26] However, as expected the impact of almost all covariates on the risk of developing PJI was the same ($P > 0.05$) when estimated with the same parametric model. The hazard of PJI (Figure 1) exhibited a monotonically decreasing profile typical of other studies investigating such occurrence [20, 27-30] and, in both databases, the log-normal model returned the best fitting of the data as this model is suitable for this type of risk profile.[31] Similarly, the log-logistic, another model suitable for decreasing hazard,[32] also returned good fitting (Table S8 and Table S9).

The observed PJI incidence in this study (~0.7% after 5 years) is lower than that sometimes reported. One possible explanation for this, was the use of a specific ICD-10 code for the identification of PJI (T84.5) while in our studies reported higher PJI rates (about 2.5% over 5 years) additional ICD-10 codes were employed to identify PJI, for example T81.4 (Infection following a procedure) T84.7 (Infection and inflammatory reaction due to other internal orthopaedic prosthetic devices, implants and grafts)[33]; however such codes appear less



For peer review only - http://bmjopen.bmj.com/site/about/guidelines.xhtml

specific and, for example, T81.4 may lead to consider a superficial infection of the surgical wound as a PJI. while M00 corresponds to a diagnosis of pyogenic arthritis [5]. When using only the presence of ICD-10 code T84.5 the PJI incidence at 5 years observed in this study is similar the 0.89% previously reported. [34]

As regression coefficients in the risk equations obtained from both databases were not statistically different for all covariates included in this study, both cohorts were pooled to increase the population size and, consequently, reduce the error associated with the coefficients estimation.[35, 36] The regression model built using the pooled cohort performance was evaluated using the area under the receiver operator curve that represents the balance between sensitivity (correctly identified cases) and specificity (correctly identified non-cases). The model performance did not significantly vary when applied to the likelihood of developing PJI over different follow-up time, probability due to the unbalance nature of the cohort in terms of patients developing PJI and patients not developing PJI. The area under the precision-recall curve (Figure 3b) instead is not impacted by unbalanced samples and the model performed better with increasing follow-up time.[37]

Stepwise selection is a classic variable reduction technique and was employed here to simplify the model minimizing the loss of performance. Negative coefficients indicate an increased risk of PJI compared to the reference; analogously, positive coefficients indicate a reduced risk of PJI compared to the reference. The greater the absolute value of the coefficient the greater the impact of the risk of PJI. Most of the risk factors identified through this approach are in line with those commonly associated to PJI (Table 1) as male gender, increased BMI and a previous PJI in the same joint or in another joint were retained in the list of covariates after stepwise selection.[2, 9, 29, 38] Age is sometimes not reported as a risk factor for PJI [9, 39], however the protective role of aging on PJI observed in this study was also seen previously. [34] A possible explanation for such correlation is "survival bias" as



For peer review only - http://bmjopen.bmj.com/site/about/guidelines.xhtml

older patients dying at higher rates would be censored at the time of death before having the opportunity of developing PJI. Smoking is sometimes reported as a risk factor PJI [9, 40] because it causes delay in wound healing but sometimes the opposite is reported [41],as here, where patient's smoking status was not included in the final model. Moreover, the active role of numerous medications provides a range of modifiable risk factors that could also inform surgery scheduling when arthroplasty is an elective procedure as the risk of PJI after NSAID and antifungal use decrease with increasing interval between the last use and the arthroplasty. The plausibility of the role of NSAID in PJI could be linked the increased cardiovascular risk resulting from their use [42] that in turn increases the risk of PJI, it is also widely accepted the immunosuppression is a general risk for the development of PJI and such NSAID role in PJI can also be linked to the immunological activity [43]. The models did not identify the use of steroidal injection in the joint as a risk associated to PJI in line with the advice of American Academy of Orthopaedic Surgeons [44]. The use of antifungal drug prior to arthroplasty has not been generally investigated as risk factor for PJI; it is possible that a prescription of antifungal drugs before the joint replacement surgery is a risk factor in our model as this would indicate a patient more susceptible to infections.

Compared to other available models, beside the UK specific population, our model includes patients both with high BMI (generally considered more at risk of PJI) or unknown BMI, a previous risk scoring model excluded patients with either missing BMI information or BMI > 50 [5]. The model developed here considered a wider range of covariates compared to the Mayo Prosthetic Joint Infection Risk Score [45] that included only diabetes or a limited number [46]. Moreover, the model presented here is not specific for specific pathogen in contrast with a *Staphylococcus aureus* specific model [47]; this particular relevant in light of the multitude of pathogens isolated in PJI such Gram+, Gram- and fungi [2]. Additionally,

For peer review only

the risk equation presented here has also consideration of a patient drug history that was very limited, if at all, in the other published models.[5, 45-47].

The model performance after stepwise selection was still high as shown by the C-statistic (Table S11) and the visual fit of the curves (Figure 4) demonstrating how the retained covariates are those pivotal in determining a patients risk to develop PJI after arthroplasty. The model developed here does not simply provide a list of risk factors for PJI but enables an objective quantification of the risk to develop PJI after hip or knee arthroplasty. As most of the identified risks are not modifiable the application of this risk scoring algorithm would not primarily allow a personalized pre-surgery drug prescription consideration but will guide follow-up care pivoting the resources (visits, test etc..) to the subgroup of patients at higher risks of PJI. Missing data have been handled through categorical binning and no mechanisms related to missingness have been hypothesised (i.e. missing at random or missing not at random); in order to avoid selection bias, the analysis has not been restricted to complete cases. Sensitivity analysis and multiple imputations could be further applied to refine the risk scoring algorithm presented.

# 8 Conclusions

Patients undergoing hip or knee arthroplasty present in CPRD AURUM and CPRD GOLD exhibit different sociodemographic characteristics and medical/drug history. However, the coefficients of parametric models, fitted to the survival curves were not statistically different allowing for pooling cohorts.

The model developed here demonstrated a good ability to identify patients at risk (based on C-statistic) and is based on the UK specific population. Such capability would allow the NHS to review the current guidelines, potentially devising a targeted program of medical treatment



For peer review only - http://bmjopen.bmj.com/site/about/guidelines.xhtml

pre-surgery, when appropriate, and of close monitoring to a small fraction of the patients after undergoing hip or knee arthroplasty allowing to reduce the incidence and impact of PJI. The newly developed risk equations will also provide contemporary inputs for the economic evaluation of new technologies for the prevention of PJI supporting health technology assessment (HTA) from The National Institute for Health and Care Excellence (NICE).

## 9 Data Availability

This study is based in part on data from the Clinical Practice Research Datalink obtained under license from the UK Medicines and Healthcare products Regulatory Agency. The data is provided by patients and collected by the NHS as part of their care and support. The interpretation and conclusions contained in this study are those of the author/s alone. The authors did not have any special access privileges that others would not have. Other researchers can apply for access to the raw data at the following URL: https://cprd.com/data-access/.

## 10 Competing interests statement

The authors have not conflict of interest to declare in the relation to this work.

## 11 Authors contribution

SP and PP conceived the idea for the study. SP carried out the statistical analysis and wrote the first draft of the manuscript. PP supervised the study and analysis. All authors reviewed and revised the manuscript. PP acts as guarantor.



For peer review only - http://bmjopen.bmj.com/site/about/guidelines.xhtml

# 12 Funding

This work has also been supported by Wellcome Trust (WT109455MA). The funder had no role in the study design, data collection and analysis, decision to publish, or preparation of the manuscript.

# 13 Research Ethics Approval

This study involves human participants and was approved by an Ethics Committee(s) or Institutional Board(s). ISAC committee from CPRD study protocol (19_009).

For peer review only

# 14 References

1. Osmon, D.R., et al., *Diagnosis and management of prosthetic joint infection: clinical practice guidelines by the Infectious Diseases Society of America.* Clin Infect Dis, 2013. **56**(1): p. e1-e25.
2. Perni, S., B. Bojan, and P. Prokopovich, *A retrospective study of risk factors, causative micro-organisms and healthcare resources consumption associated with prosthetic joint infections (PJI) using the Clinical Practice Research Datalink (CPRD) Aurum database.* PLOS ONE, 2023. **18**(3): p. e0282709.
3. Moore, A.J., et al., *Deep prosthetic joint infection: a qualitative study of the impact on patients and their experiences of revision surgery.* BMJ Open, 2015. **5**(12): p. e009495.
4. Kallala R. F., et al., *Financial analysis of revision knee surgery based on NHS tariffs and hospital costs.* The Bone & Joint Journal, 2015. **97-B**(2): p. 197-201.
5. Bülow, E., et al., *Prediction of Early Periprosthetic Joint Infection After Total Hip Arthroplasty.* Clin Epidemiol, 2022. **14**: p. 239-253.
6. Kuo, F.-C., W.-H. Hu, and Y.-J. Hu, *Periprosthetic Joint Infection Prediction via Machine Learning: Comprehensible Personalized Decision Support for Diagnosis.* The Journal of Arthroplasty, 2022. **37**(1): p. 132-141.
7. Berbari, E., et al., *The Mayo Prosthetic Joint Infection Risk Score: Implication for Surgical Site Infection Reporting and Risk Stratification.* Infection control and hospital epidemiology : the official journal of the Society of Hospital Epidemiologists of America, 2012. **33**: p. 774-81.
8. Klemt, C., et al., *The Use of Artificial Intelligence for the Prediction of Periprosthetic Joint Infection Following Aseptic Revision Total Knee Arthroplasty.* J Knee Surg, 2023(EFirst).
9. Kunutsor, S.K., et al., *Systematic review of risk prediction scores for surgical site infection or periprosthetic joint infection following joint arthroplasty.* Epidemiology & Infection, 2017. **145**(9): p. 1738-1749.
10. Taur, S.R., *Observational designs for real-world evidence studies.* Perspect Clin Res, 2022. **13**(1): p. 12-16.
11. Rassen, J.A., et al., *High-dimensional propensity scores for empirical covariate selection in secondary database studies: Planning, implementation, and reporting.* Pharmacoepidemiology and Drug Safety, 2023. **32**(2): p. 93-106.
12. Wolf, A., et al., *Data resource profile: Clinical Practice Research Datalink (CPRD) Aurum.* International Journal of Epidemiology, 2019. **48**(6): p. 1740-1740g.
13. Jick, S., et al., *Use of the CPRD Aurum Database: Insights Gained from New Data Quality Assessments.* Clin Epidemiol, 2023. **15**: p. 1219-1222.
14. CPRD. *Primary care data for public health research.* 15 March 2023]; Available from: https://cprd.com/primary-care-data-public-health-research.
15. Herrett, E., et al., *Data Resource Profile: Clinical Practice Research Datalink (CPRD).* International Journal of Epidemiology, 2015. **44**(3): p. 827-836.
16. Barzi, F., et al., *Cardiovascular risk prediction tools for populations in Asia.* J Epidemiol Community Health, 2007. **61**(2): p. 115-21.
17. R Core Team, *R: A language and environment for statistical computing.*, in *R Foundation for Statistical Computing.* 2019: Vienna, Austria.
18. Therneau T, *A Package for Survival Analysis in S. version 2.38.* 2015.
19. Bozic, K.J., et al., *Comparative Epidemiology of Revision Arthroplasty: Failed THA Poses Greater Clinical and Economic Burdens Than Failed TKA.* Clin Orthop Relat Res, 2015. **473**(6): p. 2131-8.
20. Kurtz, S.M., et al., *Prosthetic joint infection risk after TKA in the Medicare population.* Clin Orthop Relat Res, 2010. **468**(1): p. 52-6.
21. Li, T., et al., *Risk factors associated with surgical site infections following joint replacement surgery: a narrative review.* Arthroplasty, 2022. **4**(1): p. 11.
22. Garfield, K., et al., *What are the inpatient and day case costs following primary total hip replacement of patients treated for prosthetic joint infection: a matched cohort study using*

For peer review only

*linked data from the National Joint Registry and Hospital Episode Statistics.* BMC medicine, 2020. **18**(1): p. 335-335.

23. Tande, A.J. and R. Patel, *Prosthetic joint infection.* Clin Microbiol Rev, 2014. **27**(2): p. 302-45.
24. Huotari, K., M. Peltola, and E. Jämsen, *The incidence of late prosthetic joint infections: a registry-based study of 112,708 primary hip and knee replacements.* Acta Orthop, 2015. **86**(3): p. 321-5.
25. NHS Digital. *Patients Registered at GP practices in England as at 1 March 2023*. 2023; Available from: https://digital.nhs.uk/data-and-information/publications/statistical/patients-registered-at-a-gp-practice/march-2023.
26. NHS Digital. *CCG Outcomes Indicator Set - October 2022*. 2022; Available from: https://digital.nhs.uk/data-and-information/publications/statistical/ccg-outcomes-indicator-set/october-2022.
27. Iannotti, F., et al., *Prevention of Periprosthetic Joint Infection (PJI): A Clinical Practice Protocol in High-Risk Patients.* Trop Med Infect Dis, 2020. **5**(4).
28. Izakovicova, P., O. Borens, and A. Trampuz, *Periprosthetic joint infection: current concepts and outlook.* EFORT Open Rev, 2019. **4**(7): p. 482-494.
29. Lenguerrand, E., et al., *Description of the rates, trends and surgical burden associated with revision for prosthetic joint infection following primary and revision knee replacements in England and Wales: an analysis of the National Joint Registry for England, Wales, Northern Ireland and the Isle of Man.* BMJ Open, 2017. **7**(7): p. e014056.
30. Pulido, L., et al., *Periprosthetic joint infection: the incidence, timing, and predisposing factors.* Clinical orthopaedics and related research, 2008. **466**(7): p. 1710-1715.
31. Gupta, R.C., N. Kannan, and A. Raychaudhuri, *Analysis of lognormal survival data.* Mathematical Biosciences, 1997. **139**(2): p. 103-115.
32. Bennett, S., *Log-Logistic Regression Models for Survival Data.* Journal of the Royal Statistical Society Series C: Applied Statistics, 2018. **32**(2): p. 165-171.
33. Weinstein, E.J., et al., *Incidence, Microbiological Studies, and Factors Associated With Prosthetic Joint Infection After Total Knee Arthroplasty.* JAMA Network Open, 2023. **6**(10): p. e2340457-e2340457.
34. The McMaster Arthroplasty Collaborative, *Risk Factors for Periprosthetic Joint Infection Following Primary Total Hip Arthroplasty: A 15-Year, Population-Based Cohort Study.* JBJS, 2020. **102**(6): p. 503-509.
35. Khera, R., et al., *Performance of the Pooled Cohort Equations to Estimate Atherosclerotic Cardiovascular Disease Risk by Body Mass Index.* JAMA Network Open, 2020. **3**(10): p. e2023242-e2023242.
36. Vasan, R.S. and E. van den Heuvel, *Differences in estimates for 10-year risk of cardiovascular disease in Black versus White individuals with identical risk factor profiles using pooled cohort equations: an in silico cohort study.* The Lancet Digital Health, 2022. **4**(1): p. e55-e63.
37. Max Kuhn and K. Johnson, *Applied Predictive Modeling*. 2013: Springer New York, NY.
38. Lenguerrand, E., et al., *Risk factors associated with revision for prosthetic joint infection after hip replacement: a prospective observational cohort study.* The Lancet Infectious Diseases, 2018. **18**(9): p. 1004-1014.
39. Inoue, D., et al., *Age alone is not a risk factor for periprosthetic joint infection.* Journal of Hospital Infection, 2019. **103**(1): p. 64-68.
40. Bojan, B., S. Perni, and P. Prokopovich, *Systematic Review and Meta-Analysis of Tobacco Use as a Risk Factor for Prosthetic Joint Infection After Total Hip Replacement.* Arthroplast Today, 2020. **6**(4): p. 959-971.
41. Ren, X., et al., *Patients' risk factors for periprosthetic joint infection in primary total hip arthroplasty: a meta-analysis of 40 studies.* BMC Musculoskeletal Disorders, 2021. **22**(1): p. 776.
42. Varga, Z., S.R.A. Sabzwari, and V. Vargova, *Cardiovascular Risk of Nonsteroidal Anti-Inflammatory Drugs: An Under-Recognized Public Health Issue.* Cureus, 2017. **9**(4): p. e1144.

For peer review only

43. Cho, J.Y., *Immunomodulatory effect of nonsteroidal anti-inflammatory drugs (NSAIDs) at the clinically available doses.* Arch Pharm Res, 2007. **30**(1): p. 64-74.
44. American Academy of Orthopaedic Surgeons, *Diagnosis and Prevention of Periprosthetic Joint Infections - Clinical Practice Guideline.* 2019.
45. Berbari, E.F., et al., *The Mayo prosthetic joint infection risk score: implication for surgical site infection reporting and risk stratification.* Infect Control Hosp Epidemiol, 2012. **33**(8): p. 774-81.
46. Tan, T.L., et al., *Development and Evaluation of a Preoperative Risk Calculator for Periprosthetic Joint Infection Following Total Joint Arthroplasty.* JBJS, 2018. **100**(9): p. 777-785.
47. Espindola, R., et al., *Preoperative and perioperative risk factors, and risk score development for prosthetic joint infection due to Staphylococcus aureus: a multinational matched case-control study.* Clin Microbiol Infect, 2022. **28**(10): p. 1359-1366.

For peer review only



For peer review only - http://bmjopen.bmj.com/site/about/guidelines.xhtml

# 15 Figure legends

**Figure 1.** Examples of unadjusted Kaplan-Meier curves of cumulative risk of PJI (and 95% confidence intervals) for the entire cohorts (a) and stratified based on gender (b) and history of diabetes at baseline (c). Geographical distribution of PJI rates in the CPRD regions of England (d)

**Figure 2.** Coefficients for time to PJI multivariate regression with log-normal model for data from CPRD GOLD (—) and AURUM database (—).AC Active cancer; AF Atrial Fibrillation; BMI Body mass index; CKD Chronic kidney disease; COPD Chronic obstructive pulmonary disease; DMRAD Disease modifying rheumatoid arthritis drug; DOAC Direct oral anticoagulant; DVT Deep vein thrombosis; HF Heart failure; IHD Ischaemic heart disease; MI Myocardial infarction; MTX Methotrexate; NSAID Non-steroidal anti-inflammatory drug; PE Pulmonary embolism; OA Osteoarthritis; RA Rheumatoid arthritis

**Figure 3.** Time dependent (— 1 year — 2 years — 3 years — 4 years — 5 years) Receiver operating curve (ROC) (a) and Precision-Recall curves (b) of pooled log-normal equation.

**Figure 4.** Examples of Kaplan-Meier curves of risk of PJI and prediction using pooled log-normal parametric model after stepwise selection for both AURUM and GOLD CPRD database (a) gender and history of (b) Atrial Fibrillation, (c) Osteo-arthritis or (d) hypertension at baseline.

For peer review only

546 **Table 1.** Coefficients and SE of pooled lognormal parametric model of time to PJI after
547 stepwise regression.

| Variable | Coefficient | SE |
|---|---|---|
| **Log(Scale)** | 1.644 | 0.025 |
| **Intercept** | 20.724 | 0.657 |
| **Male** | -0.827 | 0.111 |
| **Age** | | |
| ≤ 45 | Reference | |
| 46 – 55 | 1.036 | 0.37 |
| 56 – 65 | 1.469 | 0.343 |
| 66 – 75 | 1.823 | 0.341 |
| 76 – 85 | 1.917 | 0.349 |
| > 85 | 3.388 | 0.452 |
| **BMI** | | |
| ≤ 20 | Reference | |
| 20 – 25 | -0.462 | 0.477 |
| 26 – 30 | -0.424 | 0.467 |
| 31 – 35 | -0.812 | 0.471 |
| 36 – 50 | -1.257 | 0.478 |
| > 50 | -1.803 | 0.886 |
| Unknown | -1.15 | 0.461 |
| **Primary arthroplasty** | | |
| Yes | Reference | |
| No | -2.558 | 0.158 |
| Unknown | -2.467 | 0.606 |
| **Admission type** | | |
| Elective | Reference | |
| A&E | -1.064 | 0.185 |
| Unknown | 0.295 | 0.976 |
| **Patella resurfacing** | | |
| No | Reference | |
| Yes | 0.393 | 0.409 |
| N/A | 0.714 | 0.118 |
| **AF** | -0.484 | 0.193 |
| **DVT** | -0.392 | 0.23 |
| **Diabetes** | -0.357 | 0.148 |
| **OA** | -0.237 | 0.125 |
| **MI** | -0.329 | 0.182 |
| **Hypertension** | -0.312 | 0.105 |
| **PJI before in same joint** | -2.585 | 0.302 |
| **PJI before in another joint** | -1.818 | 0.257 |
| **NSAID** | | |
| No Use | Reference | |

For peer review only

| | | |
|---|---|---|
| Use < 3 months | -0.768 | 0.138 |
| Use 3 - 6 months | -0.589 | 0.22 |
| Use > 6 months | 0.112 | 0.14 |
| **Antifungal** | | |
| No Use | Reference | |
| Use < 3 months | -0.725 | 0.315 |
| Use 3 - 6 months | -0.096 | 0.457 |
| Use > 6 months | -0.585 | 0.161 |

For peer review only

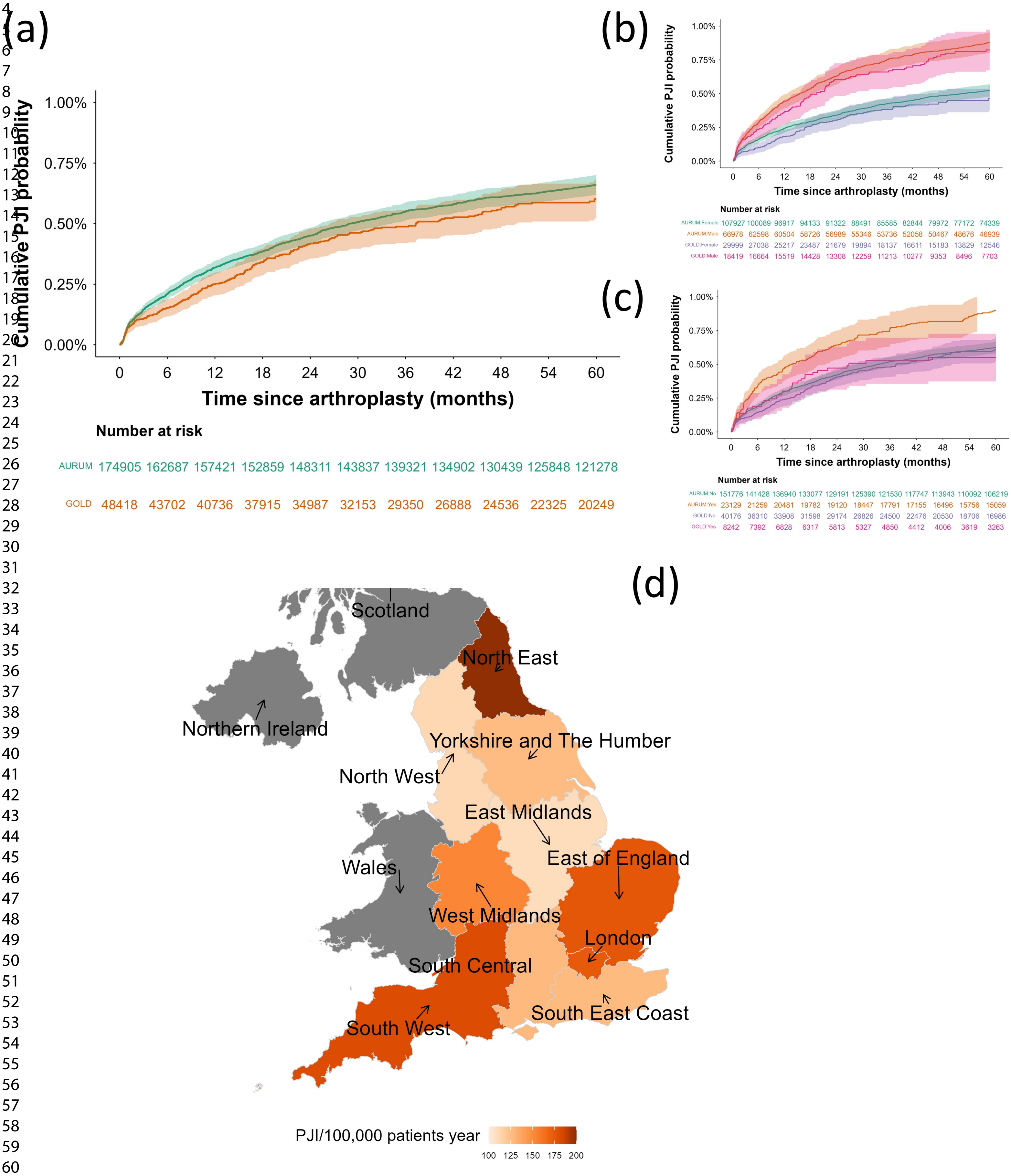
(a)
Cumulative PJI probability
1.00%
0.75%
0.50%
0.25%
0.00%
0 6 12 18 24 30 36 42 48 54 60
Time since arthroplasty (months)
Number at risk
AURUM 174905 162687 157421 152859 148311 143837 139321 134902 130439 125848 121278
GOLD 48418 43702 40736 37915 34987 32153 29350 26888 24536 22325 20249
(b)
Cumulative PJI probability
1.00%
0.75%
0.50%
0.25%
0.00%
0 6 12 18 24 30 36 42 48 54 60
Time since arthroplasty (months)
Number at risk
AURUM:Female 107927 100089 96917 94133 91322 88491 85585 82844 79972 77172 74339
AURUM:Male 66978 62598 60504 58726 56989 55346 53736 52058 50467 48676 46939
GOLD:Female 29999 27038 25217 23487 21679 19894 18137 16611 15183 13829 12546
GOLD:Male 18419 16664 15519 14428 13308 12259 11213 10277 9353 8496 7703
(c)
Cumulative PJI probability
1.00%
0.75%
0.50%
0.25%
0.00%
0 6 12 18 24 30 36 42 48 54 60
Time since arthroplasty (months)
Number at risk
AURUM:No 151776 141428 136940 133077 129191 125390 121530 117747 113943 110092 106219
AURUM:Yes 23129 21259 20481 19782 19120 18447 17791 17155 16496 15756 15059
GOLD:No 40176 36310 33908 31598 29174 26826 24500 22476 20530 18706 16986
GOLD:Yes 8242 7392 6828 6317 5813 5327 4850 4412 4006 3619 3263
(d)
Scotland
North East
Northern Ireland
Yorkshire and The Humber
North West
East Midlands
East of England
Wales
West Midlands
London
South Central
South East Coast
South West
PJI/100,000 patients year
100 125 150 175 200

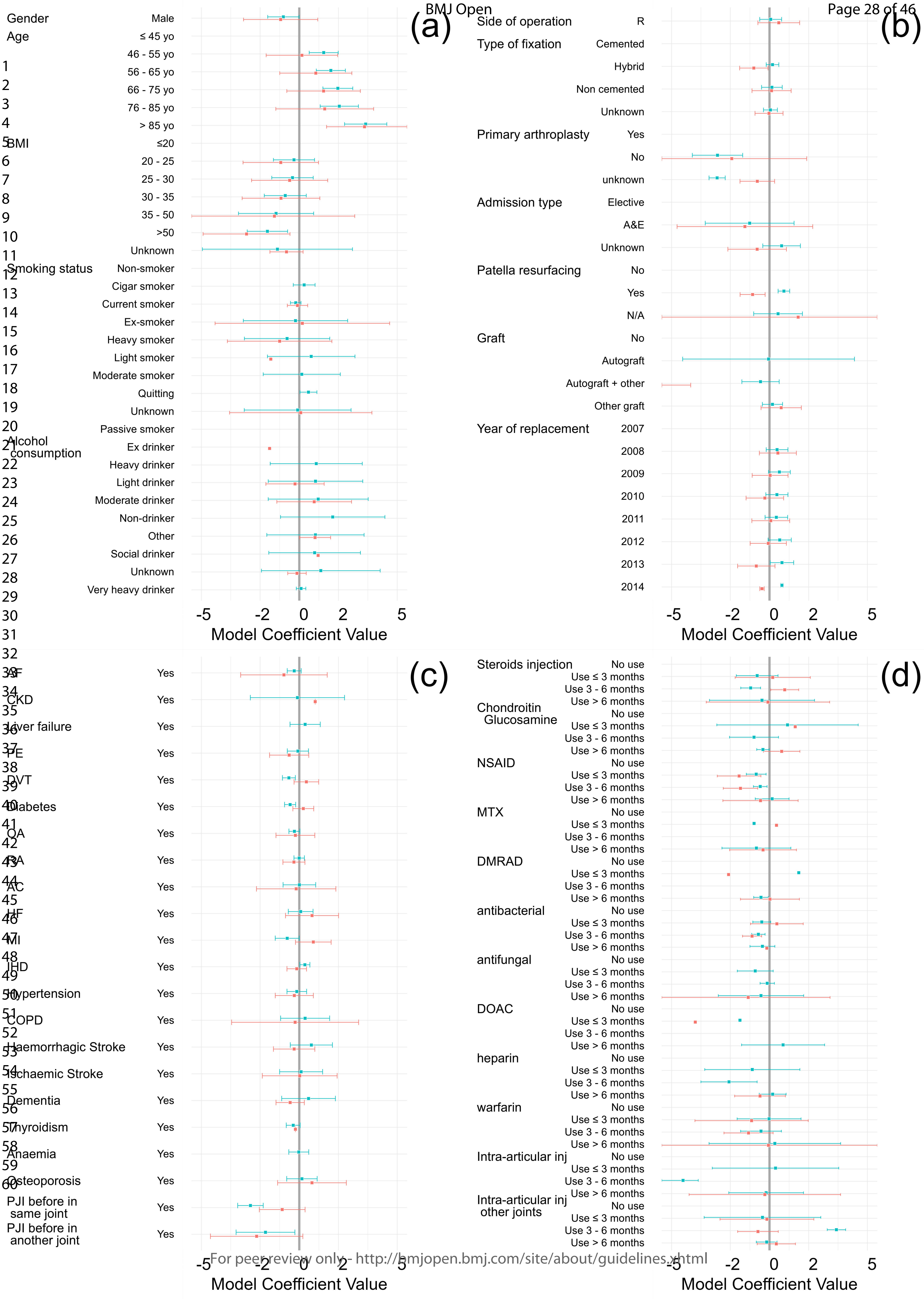
(a)
Gender
Male
Age
≤ 45 yo
46 - 55 yo
56 - 65 yo
66 - 75 yo
76 - 85 yo
> 85 yo
BMI
≤20
20 - 25
25 - 30
30 - 35
35 - 50
>50
Unknown
Smoking status
Non-smoker
Cigar smoker
Current smoker
Ex-smoker
Heavy smoker
Light smoker
Moderate smoker
Quitting
Unknown
Passive smoker
Alcohol consumption
Ex drinker
Heavy drinker
Light drinker
Moderate drinker
Non-drinker
Other
Social drinker
Unknown
Very heavy drinker
-5 -2 0 2 5
Model Coefficient Value
(b)
Side of operation
R
Type of fixation
Cemented
Hybrid
Non cemented
Unknown
Primary arthroplasty
Yes
No
unknown
Admission type
Elective
A&E
Unknown
Patella resurfacing
No
Yes
N/A
Graft
No
Autograft
Autograft + other
Other graft
Year of replacement
2007
2008
2009
2010
2011
2012
2013
2014
-5 -2 0 2 5
Model Coefficient Value
(c)
AF Yes
CKD Yes
Liver failure Yes
PE Yes
DVT Yes
Diabetes Yes
OA Yes
RA Yes
AC Yes
HF Yes
MI Yes
IHD Yes
Hypertension Yes
COPD Yes
Haemorrhagic Stroke Yes
Ischaemic Stroke Yes
Dementia Yes
Thyroidism Yes
Anaemia Yes
Osteoporosis Yes
PJI before in same joint Yes
PJI before in another joint Yes
-5 -2 0 2 5
Model Coefficient Value
(d)
Steroids injection
Chondroitin Glucosamine
NSAID
MTX
DMRAD
antibacterial
antifungal
DOAC
heparin
warfarin
Intra-articular inj
Intra-articular inj other joints
No use
Use ≤ 3 months
Use 3 - 6 months
Use > 6 months
-5 -2 0 2 5
Model Coefficient Value

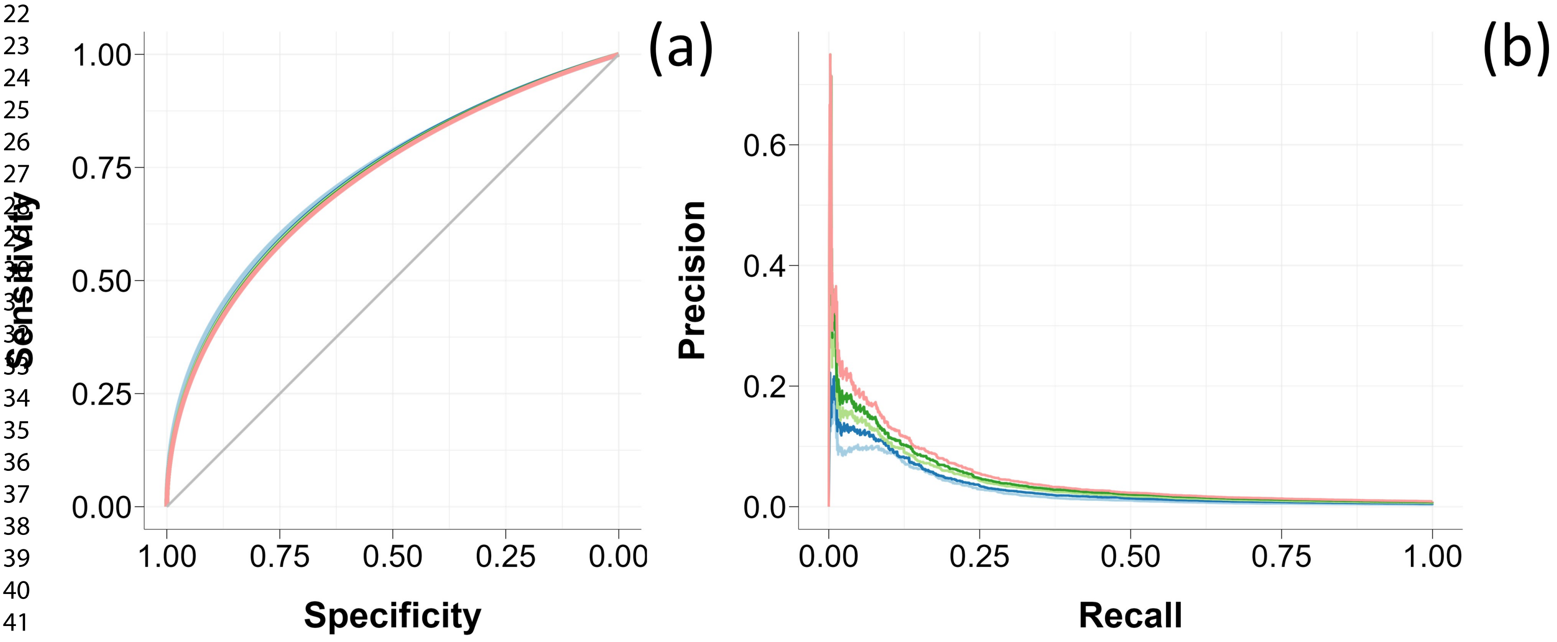
(a)
Sensitivity
1.00
0.75
0.50
0.25
0.00
Specificity
1.00
0.75
0.50
0.25
0.00
(b)
Precision
0.6
0.4
0.2
0.0
Recall
0.00
0.25
0.50
0.75
1.00

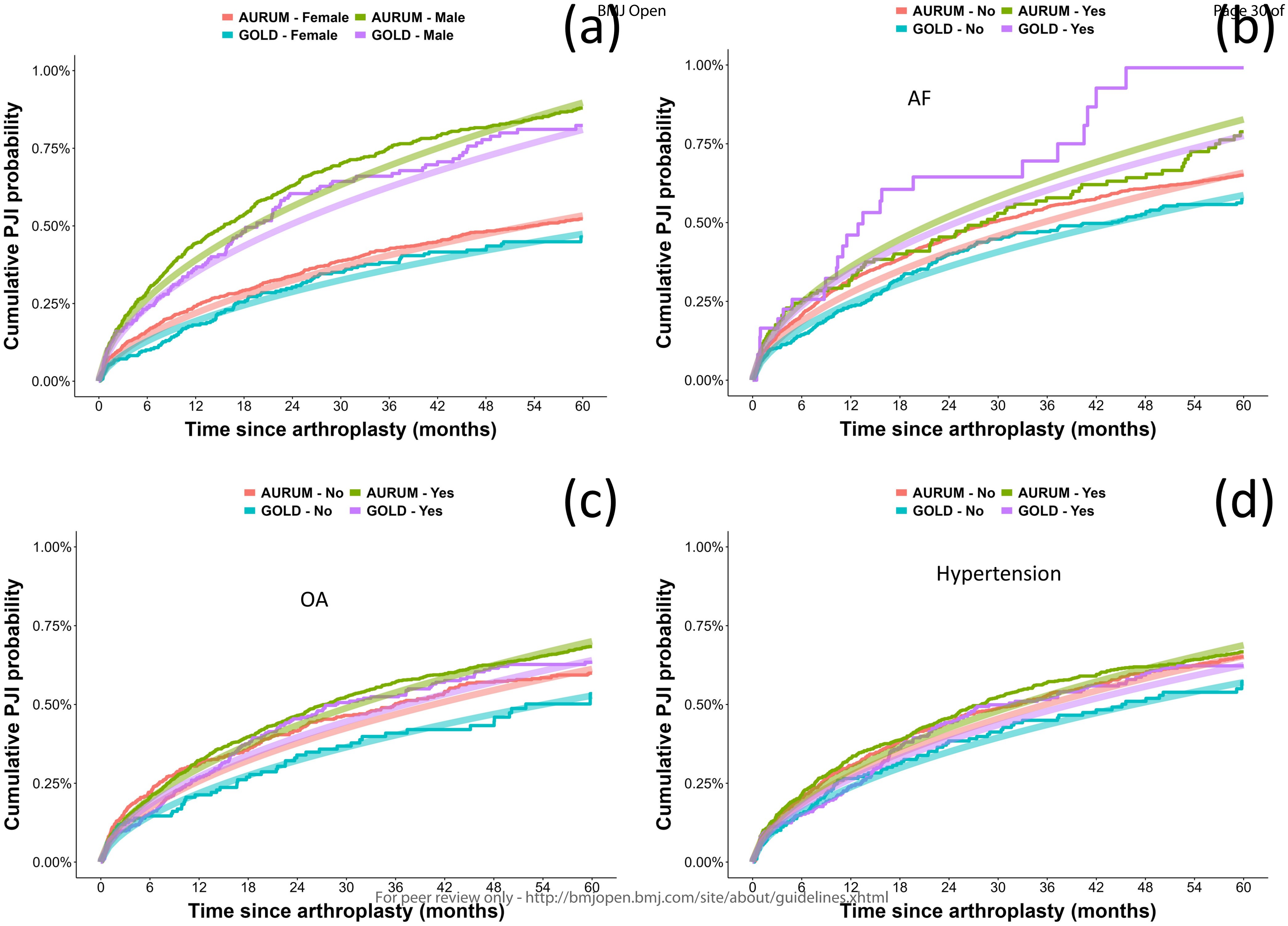
(a)
AURUM - Female
AURUM - Male
GOLD - Female
GOLD - Male
(b)
AURUM - No
AURUM - Yes
GOLD - No
GOLD - Yes
AF
(c)
OA
(d)
Hypertension
Cumulative PJI probability
1.00%
0.75%
0.50%
0.25%
0.00%
Time since arthroplasty (months)
0 6 12 18 24 30 36 42 48 54 60

# Retrospective study in UK using the Clinical Practice Research Datalink (CPRD) AURUM and GOLD database for the determination of risk equations for prosthetic joint infections (PJI)

*Stefano Perni*[1] *and Polina Prokopovich*[1,*]

School of Pharmacy and Pharmaceutical Sciences, Cardiff University, Cardiff, UK

# Supplementary Material

For peer review only

**Table S 1.** Flow of patients through initial data extraction.

| | AURUM | | GOLD | |
|---|---|---|---|---|
| | Number of joints | Number of patients | Number of joints | Number of patients |
| Knee or hip replacement in database | 330,173 | 235,249 | 88,458 | 62,672 |
| Over 31 years old at index date | 329,720 | 235,071 | 88,268 | 62,584 |
| Arthroplasty surgery after 2007 | 288,124 | 223,450 | 71,467 | 54,888 |
| With minimum 6 months lookback | 284,048 | 222,060 | 71,467 | 54,888 |
| After removing implausible data | 283,789 | 221,826 | 71,072 | 54,833 |
| Surgery before 2015 | 174,905 | 144,048 | 48,419 | 39,764 |

For peer review only

**Table S 2.** Number of joints not compleating the observation study period (5 years) and reason for loss during follow-up.

| Reason for not completing study period | AURUM | | GOLD | |
|---|---|---|---|---|
| | n | Frequency | n | Frequency |
| Developing PJI | 1,021 | 0.58% | 228 | 0.47% |
| Death | 25,657 | 14.67% | 6,021 | 12.44% |
| Last collection date before end of observation period | 749 | 0.43% | 16,604 | 34.29% |
| Device replaced for any reason | 4,108 | 2.35% | 1,090 | 2.25% |
| Transferred out of CPRD | 22,586 | 12.91% | 4,323 | 8.93% |

**Table S 3.** Follow-up (days) duration in patients not completed study period (5 years).

| | AURUM | GOLD |
|---|---|---|
| Mean (SD) | 769.61 (578.90) | 798.96 (538.44) |
| Median | 726 | 768 |
| IQR | 197 – 1,281 | 316 – 1,246 |

For peer review only

**Table S 4.** Characteristics of patients at the time of hip or knee implant surgery.

| Variable | All | AURUM | GOLD |
|---|---|---|---|
| **Gender** | | | |
| Female | 137,926 (61.76%) | 107,927 (61.71 %) | 29,999 (61.96 %) |
| Male | 85,398 (38.24%) | 66,979 (38.29 %) | 18,419 (38.04 %) |
| **Age** | | | |
| ≤ 45 | 3,221 (1.44%) | 2,593 (1.48 %) | 628 (1.30 %) |
| 46 – 55 | 13,170 (5.90%) | 10,332 (5.91 %) | 2,838 (5.86 %) |
| 56 – 65 | 43,379 (19.42%) | 34,012 (19.45 %) | 9,367 (19.35 %) |
| 66 – 75 | 75,181 (33.66%) | 58,764 (33.60 %) | 16,417 (33.91 %) |
| 76 – 85 | 65,288 (29.23%) | 51,157 (29.25 %) | 14,131 (29.19 %) |
| > 85 | 23,085 (10.34%) | 18,048 (10.32 %) | 5,037 (10.40 %) |
| Mean (SD) | 71.98 | 71.97 (10.97) | 72.03 (10.88) |
| Median | 73 | 73 | 73 |
| IQR | 65.00 - 80.00 | 65.00 - 80.00 | 65.00 - 80.00 |
| Min, max | 31.00, 109.00 | 31.00, 109.00 | 31.00, 107.00 |
| **BMI** | | | |
| ≤ 20 | 6,364 (2.85%) | 4,111 (2.35 %) | 2,253 (4.65 %) |
| 20 – 25 | 31,896 (14.28%) | 21,173 (12.11 %) | 10,723 (22.15 %) |
| 26 – 30 | 55,729 (24.95%) | 39,077 (22.34 %) | 16,652 (34.39 %) |
| 31 – 35 | 38,395 (17.19%) | 27,950 (15.98 %) | 10,445 (21.57 %) |
| 36 – 50 | 23,629 (10.58%) | 17,849 (10.20 %) | 5,780 (11.94 %) |
| > 50 | 540 (0.24%) | 472 (0.27 %) | 68 (0.14 %) |
| Unknown | 66,771 (29.90%) | 64,274 (36.75 %) | 2,497 (5.16 %) |
| Mean (SD) | 29.22 | 29.52 (6.12) | 28.52 (5.74) |
| Median | 28.6 | 28.9 | 28 |
| IQR | 25.10 - 32.70 | 25.40 - 33.00 | 24.60 - 31.90 |
| Min, max | 10.46, 73.00 | 10.46, 73.00 | 14.50, 67.50 |
| **Smoking status** | | | |
| Non-smoker | 122,373 (54.80%) | 95,096 (54.37 %) | 27,277 (56.34 %) |
| Cigar | 165 (0.07%) | 165 (0.09 %) | 0 (0.00 %) |
| Current | 12,227 (5.48%) | 8,080 (4.62 %) | 4,147 (8.56 %) |
| Ex-smoker | 70,667 (31.64%) | 54,288 (31.04 %) | 16,379 (33.83 %) |
| Heavy | 298 (0.13%) | 230 (0.13 %) | 68 (0.14 %) |
| Light | 772 (0.35%) | 573 (0.33 %) | 199 (0.41 %) |
| Moderate | 496 (0.22%) | 496 (0.28 %) | 0 (0.00 %) |
| Quitting | 674 (0.30%) | 674 (0.39 %) | 0 (0.00 %) |
| Passive smoker | 15 (0.01%) | 0 (0.00 %) | 15 (0.03 %) |
| Unknown | 15,637 (7.00%) | 15,304 (8.75 %) | 333 (0.69 %) |
| **Alcohol consumption** | | | |
| Non-drinker | 8,628 (3.86%) | 3,266 (1.87 %) | 5,362 (11.07 %) |
| Ex drinker | 384 (0.17%) | 270 (0.15 %) | 114 (0.24 %) |
| Heavy drinker | 959 (0.43%) | 899 (0.51 %) | 60 (0.12 %) |

For peer review only

| | | | |
|---|---|---|---|
| Light drinker | 40,588 (18.17%) | 32,762 (18.73 %) | 7,826 (16.16 %) |
| Moderate drinker | 7,284 (3.26%) | 5,416 (3.10 %) | 1,868 (3.86 %) |
| Social drinker | 4,080 (1.83%) | 2,891 (1.65 %) | 1,189 (2.46 %) |
| Very heavy | 690 (0.31%) | 338 (0.19 %) | 352 (0.73 %) |
| Other | 1,546 (0.69%) | 1,080 (0.62 %) | 466 (0.96 %) |
| Unknown | 159,165 (71.27%) | 127,984 (73.17 %) | 31,181 (64.40 %) |

For peer review only

**Table S 5.** Characteristics of hip or knee implant surgery.

| Variable | All | AURUM | GOLD |
|---|---|---|---|
| **Joint replaced** | | | |
| Femur | 31,766 (14.22%) | 24,963 (14.27 %) | 6,803 (14.05 %) |
| Hip | 96,267 (43.11%) | 75,439 (43.13 %) | 20,828 (43.02 %) |
| Knee | 95,291 (42.67%) | 74,504 (42.60 %) | 20,787 (42.93 %) |
| **Laterality of operation** | | | |
| Left | 104,858 (46.95%) | 82,039 (46.90 %) | 22,819 (47.13 %) |
| Right | 118,466 (53.05%) | 92,867 (53.10 %) | 25,599 (52.87 %) |
| **Fixation method** | | | |
| Cemented | 145,726 (65.25%) | 113,248 (64.75 %) | 32,478 (67.08 %) |
| Hybrid | 13,399 (6.00%) | 10,447 (5.97 %) | 2,952 (6.10 %) |
| Non cemented | 53,836 (24.11%) | 43,256 (24.73 %) | 10,580 (21.85 %) |
| Unknown | 10,363 (4.64%) | 7,955 (4.55 %) | 2,408 (4.97 %) |
| **Primary arthroplasty** | | | |
| Yes | 205,321 (91.94%) | 160,883 (91.98 %) | 44,438 (91.78 %) |
| No | 17,226 (7.71%) | 13,404 (7.66 %) | 3,822 (7.89 %) |
| Unknown | 777 (0.35%) | 619 (0.35 %) | 158 (0.33 %) |
| **Admission type** | | | |
| Elective | 184,138 (82.45%) | 144,145 (82.41 %) | 39,993 (82.60 %) |
| A&E | 38,494 (17.24%) | 30,270 (17.31 %) | 8,224 (16.99 %) |
| Unknown | 692 (0.31%) | 491 (0.28 %) | 201 (0.42 %) |
| **Patella resurfacing (* only for knee replacement surgeries)** | | | |
| No | 91,375 (40.92%) | 71,166 (40.69 %) | 20,209 (41.74 %) |
| Yes | 3,916 (1.75%) | 3,338 (1.91 %) | 578 (1.19 %) |
| **Graft** | | | |
| No | 219,645 (98.35%) | 171,999 (98.34 %) | 47,646 (98.41 %) |
| Autograft | 1,673 (0.75%) | 1,331 (0.76 %) | 342 (0.71 %) |
| Autograft + other | 67 (0.03%) | 58 (0.03 %) | 9 (0.02 %) |
| Other graft | 1,939 (0.87%) | 1,518 (0.87 %) | 421 (0.87 %) |
| **Replacement year** | | | |
| 2007 | 24,539 (10.99%) | 18,787 (10.74 %) | 5,752 (11.88 %) |
| 2008 | 26,289 (11.77%) | 20,182 (11.54 %) | 6,107 (12.61 %) |
| 2009 | 26,426 (11.83%) | 20,507 (11.72 %) | 5,919 (12.22 %) |
| 2010 | 28,061 (12.57%) | 21,742 (12.43 %) | 6,319 (13.05 %) |
| 2011 | 28,433 (12.73%) | 22,180 (12.68 %) | 6,253 (12.91 %) |
| 2012 | 29,069 (13.02%) | 22,961 (13.13 %) | 6,108 (12.62 %) |
| 2013 | 29,718 (13.31%) | 23,753 (13.58 %) | 5,965 (12.32 %) |
| 2014 | 30,788 (13.79%) | 24,793 (14.18 %) | 5,995 (12.38 %) |

For peer review only

**Table S 6.** Medical history of patients at the time of hip or knee implant surgery.

| Variable | All | AURUM | GOLD |
|---|---|---|---|
| **Atrial Fibrillation** | | | |
| No | 204,588 (91.61%) | 159,979 (91.47 %) | 44,609 (92.13 %) |
| Yes | 18,736 (8.39%) | 14,927 (8.53 %) | 3,809 (7.87 %) |
| **Liver failure** | | | |
| No | 222,841 (99.78%) | 174,529 (99.78 %) | 48,312 (99.78 %) |
| Yes | 483 (0.22%) | 377 (0.22 %) | 106 (0.22 %) |
| **CKD** | | | |
| No | 193,781 (86.77%) | 146,389 (83.70 %) | 47,392 (97.88 %) |
| Yes | 29,543 (13.23%) | 28,517 (16.30 %) | 1,026 (2.12 %) |
| **Pulmonary Embolism** | | | |
| No | 218,350 (97.77%) | 171,043 (97.79 %) | 47,307 (97.71 %) |
| Yes | 4,974 (2.23%) | 3,863 (2.21 %) | 1,111 (2.29 %) |
| **Deep Vein Thrombosis** | | | |
| No | 213,096 (95.42%) | 167,939 (96.02 %) | 45,157 (93.26 %) |
| Yes | 10,228 (4.58%) | 6,967 (3.98 %) | 3,261 (6.74 %) |
| **Diabetes** | | | |
| No | 191,953 (85.95%) | 151,777 (86.78 %) | 40,176 (82.98 %) |
| Yes | 31,371 (14.05%) | 23,129 (13.22 %) | 8,242 (17.02 %) |
| **Osteoarthritis** | | | |
| No | 72,266 (32.36%) | 55,705 (31.85 %) | 16,561 (34.20 %) |
| Yes | 151,058 (67.64%) | 119,201 (68.15 %) | 31,857 (65.80 %) |
| **Rheumatoid Arthritis** | | | |
| No | 175,330 (78.51%) | 130,011 (74.33 %) | 45,319 (93.60 %) |
| Yes | 47,994 (21.49%) | 44,895 (25.67 %) | 3,099 (6.40 %) |
| **Active Cancer** | | | |
| No | 150,190 (67.25%) | 113,646 (64.98 %) | 36,544 (75.48 %) |
| Yes | 73,134 (32.75%) | 61,260 (35.02 %) | 11,874 (24.52 %) |
| **Heart Failure** | | | |
| No | 218,251 (97.73%) | 170,804 (97.65 %) | 47,447 (97.99 %) |
| Yes | 5,073 (2.27%) | 4,102 (2.35 %) | 971 (2.01 %) |
| **Myocardial Infarction** | | | |
| No | 202,834 (90.82%) | 156,757 (89.62 %) | 46,077 (95.17 %) |
| Yes | 20,490 (9.18%) | 18,149 (10.38 %) | 2,341 (4.83 %) |
| **Ischaemic Heart Disease** | | | |
| No | 198,515 (88.89%) | 154,504 (88.34 %) | 44,011 (90.90 %) |
| Yes | 24,809 (11.11%) | 20,402 (11.66 %) | 4,407 (9.10 %) |
| **Hypertension** | | | |
| No | 99,688 (44.64%) | 79,303 (45.34 %) | 20,385 (42.10 %) |
| Yes | 123,636 (55.36%) | 95,603 (54.66 %) | 28,033 (57.90 %) |
| **COPD** | | | |
| No | 207,899 (93.09%) | 162,676 (93.01 %) | 45,223 (93.40 %) |

For peer review only

| | | | |
|---|---|---|---|
| Yes | 15,425 (6.91%) | 12,230 (6.99 %) | 3,195 (6.60 %) |
| **Haemorrhagic stroke** | | | |
| No | 219,436 (98.26%) | 171,293 (97.93 %) | 48,143 (99.43 %) |
| Yes | 3,888 (1.74%) | 3,613 (2.07 %) | 275 (0.57 %) |
| **Ischemic stroke** | | | |
| No | 215,807 (96.63%) | 170,417 (97.43 %) | 45,390 (93.75 %) |
| Yes | 7,517 (3.37%) | 4,489 (2.57 %) | 3,028 (6.25 %) |
| **Dementia** | | | |
| No | 216,525 (96.96%) | 169,637 (96.99 %) | 46,888 (96.84 %) |
| Yes | 6,799 (3.04%) | 5,269 (3.01 %) | 1,530 (3.16 %) |
| **Thyroidism** | | | |
| No | 215,894 (96.67%) | 173,581 (99.24 %) | 42,313 (87.39 %) |
| Yes | 7,430 (3.33%) | 1,325 (0.76 %) | 6,105 (12.61 %) |
| **Anaemia** | | | |
| No | 199,438 (89.30%) | 151,549 (86.65 %) | 47,889 (98.91 %) |
| Yes | 23,886 (10.70%) | 23,357 (13.35 %) | 529 (1.09 %) |
| **Osteoporosis** | | | |
| No | 205,345 (91.95%) | 161,061 (92.08 %) | 44,284 (91.46 %) |
| Yes | 17,979 (8.05%) | 13,845 (7.92 %) | 4,134 (8.54 %) |
| **PJI before in same joint** | | | |
| No | 222,353 (99.57%) | 174,100 (99.54 %) | 48,253 (99.66 %) |
| Yes | 971 (0.43%) | 806 (0.46 %) | 165 (0.34 %) |
| **PJI before in another joint (* not only hip or knee)** | | | |
| No | 220,838 (98.89%) | 173,074 (98.95 %) | 47,764 (98.65 %) |
| Yes | 2,486 (1.11%) | 1,832 (1.05 %) | 654 (1.35 %) |

For peer review only

**Table S 7.** Treatment history of patients at the time of hip or knee implant surgery.

| Variable | All | AURUM | GOLD |
|---|---|---|---|
| **Steroids injection** | | | |
| No use | 206,245 (92.35%) | 164,047 (93.79 %) | 42,198 (87.15 %) |
| Use < 3 months | 1,256 (0.56%) | 810 (0.46 %) | 446 (0.92 %) |
| Use 3 - 6 months | 2,276 (1.02%) | 1,456 (0.83 %) | 820 (1.69 %) |
| Use > 6 months | 13,547 (6.07%) | 8,593 (4.91 %) | 4,954 (10.23 %) |
| **Chondroitin glucosamine** | | | |
| No use | 219,306 (98.20%) | 173,018 (98.92 %) | 46,288 (95.60 %) |
| Use < 3 months | 930 (0.42%) | 482 (0.28 %) | 448 (0.93 %) |
| Use 3 - 6 months | 259 (0.12%) | 134 (0.08 %) | 125 (0.26 %) |
| Use > 6 months | 2,829 (1.27%) | 1,272 (0.73 %) | 1,557 (3.22 %) |
| **NSAID** | | | |
| No use | 97,243 (43.54%) | 89,492 (51.17 %) | 7,751 (16.01 %) |
| Use < 3 months | 48,354 (21.65%) | 37,077 (21.20 %) | 11,277 (23.29 %) |
| Use 3 - 6 months | 13,169 (5.90%) | 9,851 (5.63 %) | 3,318 (6.85 %) |
| Use > 6 months | 64,558 (28.91%) | 38,486 (22.00 %) | 26,072 (53.85 %) |
| **Methotrexate** | | | |
| No use | 218,503 (97.84%) | 171,414 (98.00 %) | 47,089 (97.26 %) |
| Use < 3 months | 3,255 (1.46%) | 2,559 (1.46 %) | 696 (1.44 %) |
| Use 3 - 6 months | 174 (0.08%) | 124 (0.07 %) | 50 (0.10 %) |
| Use > 6 months | 1,392 (0.62%) | 809 (0.46 %) | 583 (1.20 %) |
| **DMRAD** | | | |
| No use | 219,948 (98.49%) | 172,997 (98.91 %) | 46,951 (96.97 %) |
| Use < 3 months | 1,626 (0.73%) | 1,188 (0.68 %) | 438 (0.90 %) |
| Use 3 - 6 months | 155 (0.07%) | 112 (0.06 %) | 43 (0.09 %) |
| Use > 6 months | 1,595 (0.71%) | 609 (0.35 %) | 986 (2.04 %) |
| **Antibacterial** | | | |
| No use | 91,794 (41.10%) | 68,716 (39.29 %) | 23,078 (47.66 %) |
| Use < 3 months | 35,730 (16.00%) | 34,370 (19.65 %) | 1,360 (2.81 %) |
| Use 3 - 6 months | 15,961 (7.15%) | 14,903 (8.52 %) | 1,058 (2.19 %) |
| Use > 6 months | 79,839 (35.75%) | 56,917 (32.54 %) | 22,922 (47.34 %) |
| **Antifungal** | | | |
| No use | 192,800 (86.33%) | 148,117 (84.68 %) | 44,683 (92.29 %) |
| Use < 3 months | 4,900 (2.19%) | 4,747 (2.71 %) | 153 (0.32 %) |
| Use 3 - 6 months | 2,842 (1.27%) | 2,721 (1.56 %) | 121 (0.25 %) |
| Use > 6 months | 22,782 (10.20%) | 19,321 (11.05 %) | 3,461 (7.15 %) |
| **Direct Oral Anticoagulant** | | | |
| No use | 222,761 (99.75%) | 174,417 (99.72 %) | 48,344 (99.85 %) |
| Use < 3 months | 324 (0.15%) | 288 (0.16 %) | 36 (0.07 %) |
| Use 3 - 6 months | 54 (0.02%) | 51 (0.03 %) | 3 (0.01 %) |
| Use > 6 months | 185 (0.08%) | 150 (0.09 %) | 35 (0.07 %) |
| **Heparin** | | | |
| No use | 221,208 (99.05%) | 173,345 (99.11 %) | 47,863 (98.85 %) |

For peer review only

| | | | |
|---|---|---|---|
| Use < 3 months | 334 (0.15%) | 317 (0.18 %) | 17 (0.04 %) |
| Use 3 - 6 months | 163 (0.07%) | 142 (0.08 %) | 21 (0.04 %) |
| Use > 6 months | 1,619 (0.72%) | 1,102 (0.63 %) | 517 (1.07 %) |
| **Warfarin** | | | |
| No use | 208,608 (93.41%) | 164,436 (94.01 %) | 44,172 (91.23 %) |
| Use < 3 months | 9,494 (4.25%) | 7,499 (4.29 %) | 1,995 (4.12 %) |
| Use 3 - 6 months | 879 (0.39%) | 695 (0.40 %) | 184 (0.38 %) |
| Use > 6 months | 4,343 (1.94%) | 2,276 (1.30 %) | 2,067 (4.27 %) |
| **Intra-articular injections (n)** | | | |
| 0 | 215,281 (96.40%) | 168,424 (96.29 %) | 46,857 (96.78 %) |
| 1 | 5,856 (2.62%) | 4,709 (2.69 %) | 1,147 (2.37 %) |
| 2 | 1,320 (0.59%) | 1,063 (0.61 %) | 257 (0.53 %) |
| 3 | 444 (0.20%) | 367 (0.21 %) | 77 (0.16 %) |
| 4 | 164 (0.07%) | 130 (0.07 %) | 34 (0.07 %) |
| 5 | 117 (0.05%) | 96 (0.05 %) | 21 (0.04 %) |
| > 5 | 142 (0.06%) | 117 (0.07 %) | 25 (0.05 %) |
| **Last intra-articular injection** | | | |
| No use | 215,281 (96.40%) | 168,424 (96.29 %) | 46,857 (96.78 %) |
| Use < 3 months | 341 (0.15%) | 279 (0.16 %) | 62 (0.13 %) |
| Use 3 - 6 months | 1,309 (0.59%) | 1,036 (0.59 %) | 273 (0.56 %) |
| Use > 6 months | 6,393 (2.86%) | 5,167 (2.95 %) | 1,226 (2.53 %) |
| **Intra-articular injection in other joints (n)** | | | |
| 0 | 200,901 (89.96%) | 156,820 (89.66 %) | 44,081 (91.04 %) |
| 1 | 258 (0.12%) | 200 (0.11 %) | 58 (0.12 %) |
| 2 | 3,014 (1.35%) | 2,401 (1.37 %) | 613 (1.27 %) |
| 3 | 6,579 (2.95%) | 5,270 (3.01 %) | 1,309 (2.70 %) |
| 4 | 4,315 (1.93%) | 3,438 (1.97 %) | 877 (1.81 %) |
| 5 | 1,407 (0.63%) | 1,130 (0.65 %) | 277 (0.57 %) |
| > 5 | 6,850 (3.07%) | 5,647 (3.23 %) | 1,203 (2.48 %) |
| **Last intra-articular injection in other joints** | | | |
| No use | 200,901 (89.96%) | 156,820 (89.66 %) | 44,081 (91.04 %) |
| Use < 3 months | 974 (0.44%) | 777 (0.44 %) | 197 (0.41 %) |
| Use 3 - 6 months | 2,885 (1.29%) | 2,305 (1.32 %) | 580 (1.20 %) |
| Use > 6 months | 18,564 (8.31%) | 15,004 (8.58 %) | 3,560 (7.35 %) |

For peer review only

**Table S 8.** Summary of fitting performance measuremement for fitting results from CPRD AURUM database with different parametric models.

| Parametric model | AIC | BIC | Loglik |
|---|---|---|---|
| Exponential | 26671.5 | 27749.2 | -13730.0 |
| Gaussian | 28030.7 | 29118.5 | -15416.5 |
| Logistic | 28097.6 | 29185.4 | -14439.8 |
| Log-logistic | 26190.8 | 27278.6 | -13479.9 |
| Lognormal | 26187.4 | 27275.2 | -13459.8 |
| Weibull | 26192.6 | 27280.4 | -13480.2 |

**Table S 9**. Summary of fitting performance measuremement for fitting results from CPRD GOLD database with different parametric models.

| Parametric model | AIC | BIC | Loglik |
|---|---|---|---|
| Exponential | 7674.76 | 8573.56 | -3927.22 |
| Gaussian | 8097.86 | 9005.83 | -4134.94 |
| Logistic | 8116.71 | 9024.69 | -4146.93 |
| Log-logistic | 7543.99 | 8451.96 | -3857.65 |
| Lognormal | 7463.78 | 7793.95 | -3852.10 |
| Weibull | 7544.66 | 8452.64 | -3857.71 |

For peer review only

**Table S 10.** Regression coefficients and standard error (SE) for log-normal risk equation of time to PJI derived from CPRD AURUM and CPRD GOLD.

| Variable | AURUM | GOLD | SE AURUM | SE GOLD | *P* value |
|---|---|---|---|---|---|
| **Scale** | 5.194 | 4.759 | 1.404 | 1.650 | 0.841 |
| **Intercept** | 19.470 | 20.618 | 0.132 | 0.264 | < 0.001 |
| **Male** | -0.813 | -0.952 | 0.408 | 0.967 | 0.895 |
| **Age** | | | | | |
| ≤ 45 | Reference | | | | |
| 46 – 55 | 1.247 | 0.134 | 0.378 | 0.931 | 0.268 |
| 56 – 65 | 1.611 | 0.843 | 0.379 | 0.939 | 0.448 |
| 66 – 75 | 1.968 | 1.241 | 0.389 | 0.958 | 0.482 |
| 76 – 85 | 2.044 | 1.299 | 0.498 | 1.274 | 0.586 |
| > 85 | 3.390 | 3.329 | 0.550 | 0.988 | 0.957 |
| **BMI** | | | | | |
| ≤ 20 | Reference | | | | |
| 20 - 25 | -0.271 | -0.946 | 0.535 | 0.983 | 0.546 |
| 25 - 30 | -0.347 | -0.493 | 0.54 | 0.992 | 0.897 |
| 30 - 35 | -0.715 | -0.934 | 0.547 | 1.012 | 0.849 |
| 35 - 50 | -1.186 | -1.271 | 0.979 | 2.093 | 0.971 |
| >50 | -1.628 | -2.693 | 0.526 | 1.132 | 0.394 |
| Unknown | -1.116 | -0.653 | 1.955 | 0.432 | 0.817 |
| **Smoking status** | | | | | |
| Non-smoker | Reference | | | | |
| Cigar smoker | 0.249 | NA | 0.284 | NA | NA |
| Current smoker | -0.181 | -0.091 | 0.137 | 0.265 | 0.763 |
| Ex-smoker | -0.194 | 0.157 | 1.359 | 2.274 | 0.895 |
| Heavy smoker | -0.623 | -1.003 | 1.112 | 1.361 | 0.829 |
| Light smoker | 0.609 | -1.455 | 1.140 | 0.001 | 0.070 |
| Moderate smoker | 0.127 | NA | 1.004 | NA | NA |
| Quitting | 0.469 | NA | 0.22 | NA | NA |
| Unknown | -0.083 | 0.074 | 1.391 | 1.854 | 0.946 |
| Passive smoker | NA | NA | NA | 1.483 | NA |
| **Alcohol consumption** | | | | | |
| Non-drinker | Reference | | | | |
| Ex drinker | 0.866 | NA | 1.199 | 0.465 | NA |
| Heavy drinker | 0.828 | -0.218 | 1.231 | 0.759 | 0.47 |
| Light drinker | 0.966 | 0.761 | 1.302 | 0.973 | 0.901 |
| Moderate drinker | 1.706 | NA | 1.36 | NA | NA |
| Other | 0.825 | 0.805 | 1.267 | 0.405 | 0.988 |
| Social drinker | 0.783 | 0.962 | 1.194 | 0.002 | 0.881 |
| Unknown | 1.098 | -0.118 | 1.547 | 0.241 | 0.437 |
| Very heavy drinker | 0.091 | NA | 0.124 | 1.334 | NA |
| **Right side of operation** | 0.060 | 0.474 | 0.285 | 0.544 | 0.504 |
| **Type of fixation** | | | | | |

For peer review only

| | | | | | |
|---|---|---|---|---|---|
| Cemented | Reference | | | | |
| Hybrid | 0.155 | -0.801 | 0.164 | 0.369 | 0.018 |
| Non cemented | 0.123 | 0.106 | 0.269 | 0.51 | 0.976 |
| Unknown | 0.048 | -0.024 | 0.181 | 0.365 | 0.860 |
| **Primary arthroplasty** | | | | | |
| Yes | Reference | | | | |
| No | -2.655 | -1.927 | 0.656 | 1.953 | 0.724 |
| Unknown | -2.674 | -0.622 | 0.211 | 0.451 | < 0.001 |
| **Admission type** | | | | | |
| Elective | Reference | | | | |
| A&E | -1.013 | -1.256 | 1.157 | 1.767 | 0.908 |
| Unknown | 0.618 | -0.630 | 0.490 | 0.763 | 0.169 |
| **Patella resurfacing** | | | | | |
| No | NA | NA | NA | NA | NA |
| Yes | 0.736 | -0.862 | 0.151 | 0.326 | < 0.001 |
| N/A | 0.437 | 1.463 | 0.635 | 81880.48 | 1 |
| **Graft** | | | | | |
| No | Reference | | | | |
| Autograft | -0.048 | 26.046 | 2.238 | 2.630 | < 0.001 |
| Autograft + other | -0.458 | -6.485 | 0.486 | 1.256 | < 0.001 |
| Other graft | 0.152 | 0.597 | 0.263 | 0.525 | 0.449 |
| **Replacement year** | | | | | |
| 2007 | Reference | | | | |
| 2008 | 0.387 | 0.428 | 0.283 | 0.482 | 0.942 |
| 2009 | 0.506 | 0.031 | 0.284 | 0.467 | 0.385 |
| 2010 | 0.378 | -0.238 | 0.288 | 0.489 | 0.278 |
| 2011 | 0.35 | 0.065 | 0.294 | 0.493 | 0.62 |
| 2012 | 0.521 | -0.062 | 0.302 | 0.472 | 0.298 |
| 2013 | 0.637 | -0.674 | 0.305 | 0.487 | 0.023 |
| 2014 | 0.635 | -0.383 | 0.028 | 0.058 | < 0.001 |
| **Atrial Fibrillation** | -0.259 | -0.784 | 0.182 | 1.128 | 0.646 |
| **CKD** | -0.094 | 0.813 | 1.227 | 0 | 0.46 |
| **Liver failure** | 0.299 | NA | 0.394 | 0.751 | NA |
| **Pulmonary Embolism** | -0.071 | -0.517 | 0.276 | 0.511 | 0.443 |
| **Deep Vein Thrombosis** | -0.531 | 0.362 | 0.168 | 0.321 | 0.014 |
| **Diabetes** | -0.468 | 0.205 | 0.145 | 0.272 | 0.029 |
| **Osteoarthritis** | -0.249 | -0.199 | 0.143 | 0.506 | 0.924 |
| **Rheumatoid Arthritis** | -0.005 | -0.274 | 0.136 | 0.287 | 0.397 |
| **Active Cancer** | 0.005 | -0.159 | 0.425 | 1.034 | 0.883 |
| **Heart Failure** | 0.071 | 0.651 | 0.322 | 0.691 | 0.447 |
| **Myocardial Infarction** | -0.616 | 0.717 | 0.312 | 0.464 | 0.017 |
| **Ischaemic Heart Disease** | 0.286 | -0.130 | 0.130 | 0.255 | 0.146 |

For peer review only

| | | | | | |
|---|---|---|---|---|---|
| **Hypertension** | -0.127 | -0.254 | 0.256 | 0.496 | 0.820 |
| **COPD** | 0.298 | -0.213 | 0.639 | 1.655 | 0.773 |
| **Haemorrhagic Stroke** | 0.616 | -0.263 | 0.549 | 0.539 | 0.253 |
| **Ischaemic Stroke** | 0.094 | 0.022 | 0.563 | 0.975 | 0.949 |
| **Dementia** | 0.473 | -0.466 | 0.701 | 0.369 | 0.235 |
| **Thyroidism** | -0.309 | -0.197 | 0.182 | 0 | 0.538 |
| **Anaemia** | -0.026 | NA | 0.260 | 0.557 | NA |
| **Osteoporosis** | 0.135 | 0.648 | 0.397 | 0.895 | 0.600 |
| **PJI before in same joint** | -2.493 | -0.872 | 0.333 | 0.596 | 0.018 |
| **PJI before in another joint** | -1.726 | -2.178 | 0.766 | 1.204 | 0.751 |
| **Steroids injection** | | | | | |
| No use | Reference | | | | |
| Use ≤ 3 months | -0.629 | 0.165 | 0.543 | 0.979 | 0.478 |
| Use 3 - 6 months | -0.962 | 0.777 | 0.258 | 0.364 | < 0.001 |
| Use > 6 months | -0.383 | -0.079 | 1.368 | 1.607 | 0.885 |
| **Chondroitin Glucosamine** | | | | | |
| No use | Reference | | | | |
| Use ≤ 3 months | 0.920 | 1.314 | 1.841 | 0 | 0.831 |
| Use 3 - 6 months | -0.791 | NA | 0.638 | 0.692 | NA |
| Use > 6 months | -0.334 | 0.622 | 0.162 | 0.474 | 0.056 |
| **NSAID** | | | | | |
| No use | Reference | | | | |
| Use ≤ 3 months | -0.678 | -1.552 | 0.257 | 0.568 | 0.161 |
| Use 3 - 6 months | -0.472 | -1.482 | 0.177 | 0.448 | 0.036 |
| Use > 6 months | 0.136 | -0.459 | 0.440 | 0.980 | 0.580 |
| **Methotrexate** | | | | | |
| No use | Reference | | | | |
| Use ≤ 3 months | -0.787 | 0.358 | 0 | 0 | < 0.001 |
| Use 3 - 6 months | NA | NA | 0.771 | 0.936 | NA |
| Use > 6 months | -0.668 | -0.329 | 0.897 | 0.87 | 0.786 |
| **DMRAD** | | | | | |
| No use | Reference | | | | |
| Use ≤ 3 months | 1.494 | -2.089 | 0 | 0 | < 0.001 |
| Use 3 - 6 months | NA | NA | 0.875 | 0.826 | NA |
| Use > 6 months | -0.434 | 0.023 | 0.19 | 0.767 | 0.563 |
| **Antibacterial** | | | | | |
| No use | Reference | | | | |
| Use ≤ 3 months | -0.394 | 0.376 | 0.233 | 0.686 | 0.288 |
| Use 3 - 6 months | -0.568 | -0.889 | 0.174 | 0.249 | 0.291 |
| Use > 6 months | -0.362 | -0.136 | 0.324 | 0 | 0.485 |
| **Antifungal** | | | | | |
| No use | Reference | | | | |
| Use ≤ 3 months | -0.728 | NA | 0.467 | 0 | NA |

For peer review only

| | | | | | |
|---|---|---|---|---|---|
| Use 3 - 6 months | -0.115 | NA | 0.186 | 0.364 | NA |
| Use > 6 months | -0.434 | -1.086 | 1.112 | 2.132 | 0.786 |
| **Direct Oral Anticoagulant** | | | | | |
| No use | Reference | | | | |
| Use ≤ 3 months | -1.508 | -3.789 | 0 | 0 | < 0.001 |
| Use 3 - 6 months | NA | NA | 2.132 | 0 | NA |
| Use > 6 months | 0.694 | NA | 1.081 | 0 | NA |
| **Heparin** | | | | | |
| No use | Reference | | | | |
| Use ≤ 3 months | -0.878 | NA | 1.239 | 0 | NA |
| Use 3 - 6 months | -2.067 | NA | 0.732 | 1.011 | NA |
| Use > 6 months | 0.162 | -0.484 | 0.350 | 0.661 | 0.388 |
| **Warfarin** | | | | | |
| No use | Reference | | | | |
| Use ≤ 3 months | -0.021 | -0.914 | 0.832 | 1.476 | 0.598 |
| Use 3 - 6 months | -0.433 | -1.07 | 0.531 | 0.640 | 0.444 |
| Use > 6 months | 0.273 | -0.053 | 1.714 | 3527.096 | 1 |
| **Intra-articular injection** | | | | | |
| No use | Reference | | | | |
| Use ≤ 3 months | 0.308 | 19.912 | 1.647 | 0 | < 0.001 |
| Use 3 - 6 months | -4.414 | NA | 0.396 | 0.915 | NA |
| Use > 6 months | -0.171 | -0.238 | 0.973 | 1.973 | 0.976 |
| **Intra-articular injections in other joints** | | | | | |
| No use | Reference | | | | |
| Use ≤ 3 months | -0.358 | -0.131 | 1.519 | 1.222 | 0.907 |
| Use 3 - 6 months | 3.417 | -0.589 | 0.239 | 0.529 | < 0.001 |
| Use > 6 months | -0.144 | 0.349 | 0.270 | 0.498 | 0.384 |

For peer review only

**Table S 11.** C- statistic values for pooled risk equation at different follow-up.

| Follow-up | Mean | 95% CI |
|---|---|---|
| 1 year | 0.733 | 0.712 - 0.756 |
| 2 years | 0.727 | 0.708 - 0.744 |
| 3 years | 0.725 | 0.708 - 0.743 |
| 4 years | 0.723 | 0.708 - 0.738 |
| 5 years | 0.721 | 0.706 - 0.736 |

For peer review only

# TRIPOD Checklist: Prediction Model Development and Validation

| Section/Topic | Item | | Checklist Item | Page |
|---|---|---|---|---|
| **Title and abstract** | | | | |
| Title | 1 | D;V | Identify the study as developing and/or validating a multivariable prediction model, the target population, and the outcome to be predicted. | 1 |
| Abstract | 2 | D;V | Provide a summary of objectives, study design, setting, participants, sample size, predictors, outcome, statistical analysis, results, and conclusions. | 2 |
| **Introduction** | | | | |
| Background and objectives | 3a | D;V | Explain the medical context (including whether diagnostic or prognostic) and rationale for developing or validating the multivariable prediction model, including references to existing models. | 4 |
| | 3b | D;V | Specify the objectives, including whether the study describes the development or validation of the model or both. | 4-5 |
| **Methods** | | | | |
| Source of data | 4a | D;V | Describe the study design or source of data (e.g., randomized trial, cohort, or registry data), separately for the development and validation data sets, if applicable. | 5 |
| | 4b | D;V | Specify the key study dates, including start of accrual; end of accrual; and, if applicable, end of follow-up. | 6 |
| Participants | 5a | D;V | Specify key elements of the study setting (e.g., primary care, secondary care, general population) including number and location of centres. | 5-6 |
| | 5b | D;V | Describe eligibility criteria for participants. | 6 |
| | 5c | D;V | Give details of treatments received, if relevant. | 6 |
| Outcome | 6a | D;V | Clearly define the outcome that is predicted by the prediction model, including how and when assessed. | 7 |
| | 6b | D;V | Report any actions to blind assessment of the outcome to be predicted. | NA |
| Predictors | 7a | D;V | Clearly define all predictors used in developing or validating the multivariable prediction model, including how and when they were measured. | 6-7 |
| | 7b | D;V | Report any actions to blind assessment of predictors for the outcome and other predictors. | NA |
| Sample size | 8 | D;V | Explain how the study size was arrived at. | 9 |
| Missing data | 9 | D;V | Describe how missing data were handled (e.g., complete-case analysis, single imputation, multiple imputation) with details of any imputation method. | 7 |
| Statistical analysis methods | 10a | D | Describe how predictors were handled in the analyses. | 8 |
| | 10b | D | Specify type of model, all model-building procedures (including any predictor selection), and method for internal validation. | 7-8 |
| | 10c | V | For validation, describe how the predictions were calculated. | 7-8 |
| | 10d | D;V | Specify all measures used to assess model performance and, if relevant, to compare multiple models. | 8 |
| | 10e | V | Describe any model updating (e.g., recalibration) arising from the validation, if done. | NA |
| Risk groups | 11 | D;V | Provide details on how risk groups were created, if done. | NA |
| Development vs. validation | 12 | V | For validation, identify any differences from the development data in setting, eligibility criteria, outcome, and predictors. | 8 |
| **Results** | | | | |
| Participants | 13a | D;V | Describe the flow of participants through the study, including the number of participants with and without the outcome and, if applicable, a summary of the follow-up time. A diagram may be helpful. | 9 |
| | 13b | D;V | Describe the characteristics of the participants (basic demographics, clinical features, available predictors), including the number of participants with missing data for predictors and outcome. | 9-10 |
| | 13c | V | For validation, show a comparison with the development data of the distribution of important variables (demographics, predictors and outcome). | NA |
| Model development | 14a | D | Specify the number of participants and outcome events in each analysis. | 10 |
| | 14b | D | If done, report the unadjusted association between each candidate predictor and outcome. | NA |
| Model specification | 15a | D | Present the full prediction model to allow predictions for individuals (i.e., all regression coefficients, and model intercept or baseline survival at a given time point). | Table 1 |
| | 15b | D | Explain how to the use the prediction model. | 9-10 |
| Model performance | 16 | D;V | Report performance measures (with CIs) for the prediction model. | 10-11 |
| Model-updating | 17 | V | If done, report the results from any model updating (i.e., model specification, model performance). | NA |
| **Discussion** | | | | |
| Limitations | 18 | D;V | Discuss any limitations of the study (such as nonrepresentative sample, few events per predictor, missing data). | 13 |
| Interpretation | 19a | V | For validation, discuss the results with reference to performance in the development data, and any other validation data. | 12 |
| | 19b | D;V | Give an overall interpretation of the results, considering objectives, limitations, results from similar studies, and other relevant evidence. | 11-13 |
| Implications | 20 | D;V | Discuss the potential clinical use of the model and implications for future research. | 11-14 |
| **Other information** | | | | |
| Supplementary information | 21 | D;V | Provide information about the availability of supplementary resources, such as study protocol, Web calculator, and data sets. | 14 |
| Funding | 22 | D;V | Give the source of funding and the role of the funders for the present study. | 14 |

*Items relevant only to the development of a prediction model are denoted by D, items relating solely to a validation of a prediction model are denoted by V, and items relating to both are denoted D;V. We recommend using the TRIPOD Checklist in conjunction with the TRIPOD Explanation and Elaboration document.

For peer review only - http://bmjopen.bmj.com/site/about/guidelines.xhtml

BMJ Open

# Retrospective study in UK using the Clinical Practice Research Datalink (CPRD) AURUM and GOLD database for the determination of risk equations for prosthetic joint infections (PJI)



| | |
|---|---|
| Journal: | *BMJ Open* |
| Manuscript ID | bmjopen-2023-082501.R2 |
| Article Type: | Original research |
| Date Submitted by the Author: | 25-Apr-2024 |
| Complete List of Authors: | Perni, Stefano; Cardiff University<br>Prokopovich, Polina; Cardiff University |
| <b>Primary Subject Heading</b>: | Infectious diseases |
| Secondary Subject Heading: | Qualitative research, Surgery, Epidemiology, Medical management |
| Keywords: | Epidemiology < INFECTIOUS DISEASES, Hip < ORTHOPAEDIC & TRAUMA SURGERY, Electronic Health Records, Knee < ORTHOPAEDIC & TRAUMA SURGERY |
| | |



SCHOLARONE™
Manuscripts

*I, the Submitting Author has the right to grant and does grant on behalf of all authors of the Work (as defined in the below author licence), an exclusive licence and/or a non-exclusive licence for contributions from authors who are: i) UK Crown employees; ii) where BMJ has agreed a CC-BY licence shall apply, and/or iii) in accordance with the terms applicable for US Federal Government officers or employees acting as part of their official duties; on a worldwide, perpetual, irrevocable, royalty-free basis to BMJ Publishing Group Ltd ("BMJ") its licensees and where the relevant Journal is co-owned by BMJ to the co-owners of the Journal, to publish the Work in this journal and any other BMJ products and to exploit all rights, as set out in our licence.*

*The Submitting Author accepts and understands that any supply made under these terms is made by BMJ to the Submitting Author unless you are acting as an employee on behalf of your employer or a postgraduate student of an affiliated institution which is paying any applicable article publishing charge ("APC") for Open Access articles. Where the Submitting Author wishes to make the Work available on an Open Access basis (and intends to pay the relevant APC), the terms of reuse of such Open Access shall be governed by a Creative Commons licence – details of these licences and which Creative Commons licence will apply to this Work are set out in our licence referred to above.*

*Other than as permitted in any relevant BMJ Author's Self Archiving Policies, I confirm this Work has not been accepted for publication elsewhere, is not being considered for publication elsewhere and does not duplicate material already published. I confirm all authors consent to publication of this Work and authorise the granting of this licence.*

For peer review only

# Retrospective study in UK using the Clinical Practice Research Datalink (CPRD) AURUM and GOLD database for the determination of risk equations for prosthetic joint infections (PJI)

*Stefano Perni[1] and Polina Prokopovich[1,*]*

[1] School of Pharmacy and Pharmaceutical Sciences, Cardiff University, Cardiff, UK

WORD COUNT: 3948

**Corresponding author: Dr Polina Prokopovich**

School of Pharmacy and Pharmaceutical Sciences

Cardiff University

Redwood Building, King Edward VII Avenue

Cardiff, UK

CF10 3NB

E-mail address: prokopovichp@cf.ac.uk



For peer review only - http://bmjopen.bmj.com/site/about/guidelines.xhtml

# 1 Abstract

**Background:** Prosthetic joint infections (PJI) are a serious negative outcome of arthroplasty with incidence of about 1%. Risk of PJI could depend on local treatment policies and guidelines; no UK specific risk scoring is currently available.

**Objective:** to use electronic health records to determine a risk quantification model for the development of PJI

**Design:** Records in Clinical Practice Research Datalink (CPRD) GOLD and AURUM of patients undergoing hip or knee arthroplasty between Jan 2007 and Dec 2015, with linkage to Hospital episode statistic (HES) and office of national statistic (ONS), were obtained. Cohorts' characteristics and risk equations through parametric models were developed and compared between the two databases. Pooled cohort risk equations were determined for the UK population and simplified through stepwise selection.

**Results:** After applying the inclusion/exclusion criteria, 174,905 joint (1,021 developed PJI) were identified in CPRD AURUM and 48,419 joint (228 developed PJI) in CPRD GOLD. Patients undergoing hip or knee arthroplasty in both databases exhibited different sociodemographic characteristics and medical/drug history. However, the quantification of the impact of such covariates (coefficients of parametric models fitted to the survival curves) in the risk of PJI were not statistically different between the two cohorts. The log-normal model fitted to the pooled cohorts after stepwise selection had a C-statistic > 0.7.

**Conclusions:** the risk prediction tool developed here could help preventing PJI through identifying modifiable risk factors pre-surgery and identifying the patients most likely to benefit from close monitoring/preventive actions. As derived from the UK population, such tool will help the NHS reducing the impact of PJI on its resources and patient lives.

**Keywords:** PJI, CPRD, pooled cohort equations, risk prediction



For peer review only - http://bmjopen.bmj.com/site/about/guidelines.xhtml

# 2 List of abbreviations

| | |
|---|---|
| AC | Active cancer |
| A&E | Accident and Emergency |
| AF | Atrial Fibrillation |
| AIC | Akaike Information Criterion |
| BIC | Bayesian Information Criterion |
| BMI | Body mass index |
| CKD | Chronic kidney disease |
| COPD | Chronic obstructive pulmonary disease |
| CPRD | Clinical Practice Research Datalink |
| DMRAD | Disease modifying rheumatoid arthritis drug |
| DOAC | Direct oral anticoagulant |
| DVT | Deep vein thrombosis |
| HES | Hospital episode statistic |
| HF | Heart failure |
| HTA | Health technology assessment |
| IHD | Ischaemic heart disease |
| MI | Myocardial infarction |
| MTX | Methotrexate |
| NHS | National health service |
| NICE | National Institute for Health and Care Excellence |
| NSAID | Non-steroidal anti-inflammatory drug |
| PE | Pulmonary embolism |
| PJI | Prostatic joint infection |
| OA | Osteoarthritis |
| ONS | Office national statistic |
| RA | Rheumatoid arthritis |

For peer review only



For peer review only - http://bmjopen.bmj.com/site/about/guidelines.xhtml

# 3 Strengths and Limitations

- We sampled a large cohort of patients and considered a wide-range of covariates and of drugs to establish the medical and medication history.
- The specific geographical location (UK) of the patients analysed may limit the availability of data regarding the impact of ethnicity and not have the same predictive power when applied to other countries.
- It is plausible some confounding variables are unavailable, either because they are not routinely recorded (for example, nutritional status and genetic factors) or unavailable (for example, hospital prescribing and use of over-the-counter medications).
- The use of registry-based data incurs in the possibility of error in the recording of data or codes may be a source of information bias.
- Our study was observational thus only able to identify correlations between PJI and the included covariates not causality.



For peer review only

# 4 Introduction

Infections associated to arthroplasty, known as prosthetic joint infections (PJIs), have an incidence of about 1% after primary arthroplasty, this is about 5 times higher after secondary arthroplasty not resulting from a previous PJI (aseptic replacement) while about 10 time after a revision caused by a PJI.[1, 2] The consequences of developing a PJI can be extremely debilitating long-term for patients [3] and, with an estimate mean cost to the NHS of ~£30,000 per PJI in knee joints [4] and ~£24,000 for PJI in hips, also health care providers incur high costs.

Numerous risk factors for PJI have been identified and risk quantification tools developed [5-9] as the possibility of identifying patients at risks for PJI would allow a more proactive and targeted approach to prevention; however, the quantification of an individual probability to develop PJI is often impacted by the size of the cohort used in deriving such risk equations and the covariates available in the datasets employed for such analysis.[10, 11] Such limitations reduce the ability to translate conclusions to other settings/countries and limit the accuracy of the estimated risk for patients.

Moreover, UK specific risk and contemporary equations have not been developed yet and such PJI risk estimates are calculated based on results from different countries (such as Sweden [5]) where treatment algorithms and local medical guidelines may differ from UK. Clinical Practice Research Datalink (CPRD) is a UK government not-for-profit research service; it collects health records from participating GP practices that have agreed to contribute data to this database related to diagnoses, symptoms, prescriptions, referrals and tests for patients. It has provided anonymized primary care data to conduct public health research.[12] CPRD GOLD and AURUM database have been shown to be a data source with quality and completeness [13]. CPRD also provide linkage to Office of National statistics (ONS), providing accurate date and cause of death, along with Hospital episode statistics



For peer review only - http://bmjopen.bmj.com/site/about/guidelines.xhtml

(HES) contains records of secondary care activities including dates, specialty, clinical diagnoses and procedures.[12] Due to some differences in the structure and clinical coding of IT systems employed by the GP practices,[14] CPRD data are provided as separate databases: CPRD GOLD contains data contributed by practices using Vision® software, [15] while CPRD AURUM contains data from practices using EMIS® Web.[12] In September 2018, CPRD AURUM included records of 19 million patients registered in 738 practices (~ 10% of all English practices), of whom 7 million were alive and actively contributing (13% of the population of England). In 2013, CPRD GOLD contained records from 11 million patients registered in 674 practices; including 4.4 million patients active (alive and currently registered), approximately 6.9% of the UK population.[15] Therefore, they both offer a representative population of UK patients; however when linkage to HES and ONS is employed, only patients registered in GP practices located in England are represented in the final dataset.

Because of the lack of contemporary and UK specific rick equations for the development of PJI, the objectives of this study were multiples. Firstly, we wanted to compare the characteristics of patients developing PJI in both CPRD databases, then to estimate risk equations from each database and compare the contribution of each individual covariate in both databases to assess the possibility of pooling participants. Finally, relevant risk factors were identified from a stepwise regression of the pooled cohorts risk equation and the predictive ability of the newly developed risk equation assessed.



For peer review only - http://bmjopen.bmj.com/site/about/guidelines.xhtml

# 5 Methods

## 5.1 Data sources

Data were obtained from both databases available in the CPRD; Furthermore, CPRD data were linked to HES secondary care data and ONS mortality data.

This study protocol (19_009) was reviewed by the Independent Scientific Advisory Committee (ISAC) and approved in January 2020; a minor amendment was subsequently approved on 1st December 2021. Data were extracted in December 2020. The research team had access to only de-identified data therefore the requirement for patient consent was waived.

## 5.2 Study design and population

This was a retrospective study of patients undergoing hip or knee arthroplasty; the index date for inclusion was the day of arthroplasty. Patients had to be aged ≥ 30 years at index date and have arthroplasty between 01/01/2007 and 31/12/2014. Patients were excluded from the study if they were registered on the CPRD for < 6 months prior to index date with the most recent CPRD up-to-standard (UTS) date > 6 months prior to index date.

This research is largely descriptive rather than inferential in nature in that it aims to characterize patterns of PJI and verify the generalizability of existing risk equations. A sample size and power calculation have, therefore, not been undertaken with respect to identifying differences in outcomes between groups.

Eligible patients were identified through OPCS Classification of Interventions and Procedures (OPCS-4) codes [2] for hip or knee arthroplasty in HES records and they were followed-up for 5 years or the first occurrence of: device replaced, death or loss to follow-up. Loss to follow-up was be defined as the date a patient was transferred out of the practice, the date that the practice left the database, or the last recorded event date.



For peer review only - http://bmjopen.bmj.com/site/about/guidelines.xhtml

## 5.3 Study measures

Covariate related to patient characteristics were extracted from the CPRD database; surgery properties were derived from the HES database according to OPCS codes (for type of fixation, laterality fixation, grafts ...) and admission codes. Medical history was determined by the presence before index date of disease specific ICD-10 codes in HES or either MedCodeID codes in the CPRD AURUM database or READ codes in CPRD GOLD database. Prescription history was assessed from relevant BNF codes reported in the CPRD database.[2] Data cleaning was performed to remove implausible entries such as day of death before index date, and body mass index (BMI) < 10 or BMI > 80.

PJI occurrence was assess by the presence of the ICD-10 code T84.5 in the HES database; the date of PJI occurrence was set as the day of hospitalization. To ascertain that PJI diagnosed were affecting the joint of interest as patients could have multiple devices, only a diagnosis of PJI and a record to OPCS any procedure in the joint of interest during the hospitalization were considered.

## 5.4 Missing data handling

Medical history was based on the presence of a specific diagnostic code, therefore the absence of such codes in a patient records was assumed to represent absence of such event, therefore no missing data were possible in a patient medical history. On the contrary, when a record was expected, such in case of BMI, age, primary or secondary arthroplasty along with alcohol and smoking status, the absence of any entry resulted in this patient having the covariate under consideration categorized as missing. Missing data were handled through categorical binning and no imputation methods applied.



For peer review only - http://bmjopen.bmj.com/site/about/guidelines.xhtml

## 5.5 Survival analysis

Survival curves for each database were fitted with parametric models (Exponential, Weibull, normal, log-normal, logistic, log-logistic) and ranked according to Akaike information criteria (AIC) and Bayesian information criteria (BIC).

Stepwise regression was employed to reduce the number of covariates in the risk equation using AIC as criteria to identify which variable to add/remove or to stop the selection process.

## 5.6 Statistical analysis

Descriptive analyses were generated, characterizing patient demographics, clinical and treatment characteristics. Summary statistics (e.g. mean, standard deviation, standard error, median, inter-quartile range, minimum, and maximum) were calculated for continuous variables (age and BMI) while number and proportion/percentage were calculated for categorical variables. For multivariate regression, age was categorised as ≤ 45, 46 – 55, 56 – 65, 66 – 75, 76 – 85 and > 85 while BMI was categorised as ≤ 20, 20 – 25, 26 – 30, 31 – 35, 36 – 50 and > 50. The number and proportion of patients with missing data are also determined for each of the variables of interest.

Kaplan-Meier curves were generated, and the log-rank test was used to compare survival curves between the CPRD AURUM and CPRD GOLD databases.

PJI rates were calculated dividing the number of PJI cases observed by the total duration of the follow-up.

Z-test for each factor was performed to compare of the regression coefficients between risk equations developed using CPRD AURUM and CPRD GOLD.[16]

$$Z_i = \frac{\beta_{i,AURUM} - \beta_{i,GOLD}}{\sqrt{SE_{i,AURUM}^2 + SE_{i,GOLD}^2}}$$

Where:

For peer review only

175 $\beta_i$ = regression coefficient for factor *i*

176 $SE_i$ = Standard error of regression coefficient for factor *i*

177

178 The predictive ability of the final risk equation was assess calculating the area under the precision-recall and receiver-operator curves at time-varying follow-ups. C-statistic was evaluated after different follow-up; 95% confidence intervals were estimated through bootstrapping with 500 replicates for each timepoint.

All data collection, analysis and visualization were performed using R (ver 4.2.2) and relevant packages.[17, 18]

## 5.7 Patient and public involvement

Patients and/or the public were not involved in the design, or conduct, or reporting, or dissemination plans of this research.



For peer review only - http://bmjopen.bmj.com/site/about/guidelines.xhtml

# 6 Results

## 6.1 Patient counts

After linkage with both HES and ONS, records for 330,173 hip of knee arthroplasty, corresponding to 235,249 patients were identified in CPRD AURUM; similarly, CPRD GOLD contained information for 88,458 hip or knee joint subjected to arthroplasty related to 62,672 patients. After applying the inclusion/exclusion criteria the records of 174,905 joint (144,048 patients) were identified in CPRD AURUM and 48,419 joint (39,764 patients) in CPRD GOLD (Table S1).

In patients indexed in either CPRD AURUM or GOLD, the most common reason for not completing the study observation period (5 years) was death, 14.67% and 12.44 % in CPRD AURUM and GOLD, respectively. The second most common reason in CPRD AURUM was transfer out of the CPRD database (in 12.91% of cases) while in CPRD GOLD it was data collection terminated before end-of study (in 34.29 % of cases); replacement of the implanted device in the first 5 years after surgery occurred in about 2.3 % of cases in both databases. A diagnosis of PJI in the first years after implant occurred in 1,021 joint (0.58 %) in CPRD AURUM and 228 (0.47 %) joints in CPRD GOLD (Table S2).

Patients not completing the study observation period were follow-up for a median of 726 and 768 days in CPRD AURUM and GOLD, respectively (Table S3).

## 6.2 Baseline characteristics

The demographic and basic characteristics of patients in both CPRD databases were generally similar for all covariates tested (e.g. gender, age, BMI, alcohol and smoking status) (Table S4); the level on unknown BMI was greater in AURUM than GOLD. The characteristics of the arthroplasty exhibited similar distribution in both databases (Table S5).

For peer review only

212 Patients undergoing hip or knee arthroplasty had similar prevalence of most of the diseases included in this study, however chronic kidney disease (CKD), rheumatoid arthritis (RA), myocardial infarction (MI), anaemia and cancer had different distributions in AURUM and GOLD (Table S6). Moreover, the also history of prescriptions in patients with hip and knee arthroplasty in CPRD AURUM and GOLD were comparable (Table S7).

## 6.3 PJI occurrence

Unadjusted Kaplan-Meier curve for time to PJI diagnosis showed a decreasing hazard rate with time; 0.21% and 0.32% of joints in CPRD AURUM developed PJI in the first 6 and 12 months, respectively. After 2 and 4 years the proportion of joints with PJI was 0.45% and 0.61% respectively. The probability of developing PJI in the joints from CPRD GOLD was 0.15%, 0.25%, 0.42% and 0.56% after 6 months, 1 year, 2 years and 4 years respectively. The log-rank test between the two curves had $P < 0.001$ (Figure 1a).

Likewise, the Kaplan-Meier curves for time to PJI in the two CPRD databases exhibited comparable patterns with $P$ values generally < 0.001 (Figure 1b and c).

The distribution of the PJI rate different across the different England regions defined in CPRD ranging from ~100 PJI cases/100,000 patients year in the East Midlands to ~180 PJI cases/100,000 patients year in the North East (Figure 1d).

## 6.4 Risk equations

Survival curves of time to PJI diagnosis in CPRD GOLD and CPRD AURUM were fitted independently with parametric models; for both databases the log-normal distribution returned the best fitting (e.g. lowest AIC, lowest BIC and highest loglik) (Table S8 and Table S9). The coefficients of the log-normal parametric models for both databases were compared with Z-test and generally $P > 0.05$ (Figure 2 and Table S10).



For peer review only - http://bmjopen.bmj.com/site/about/guidelines.xhtml

The risk equations based on the log-normal survival model developed using the pooled cohorts had performance in categorizing a patients as at risk of PJI or not over different follow-up times increasing with longer periods as revealed by the area under the precision-recall curve; the area under the receiver operator curve (AUC) and C-statistic did not exhibit the same pattern of increasing performance with longer follow-up instead (Figure 3 and Table S11).

The stepwise variable selection was employed to reduce the number of covariates in the risk equation to those most significant. The final list of coefficients revealed that gender, age, BMI were patient characteristics that represent risk factors for PJI. The type of surgery (primary or secondary), admission through accident and emergency (A&E) or elective and the resurfacing of the patella were the risk factors related to the surgical procedure. A history of atrial fibrillation (AF), deep vein thrombosis (DVT), diabetes, osteoarthritis (OA), MI and hypertension were all increasing the risk of PJI. Previous PJIs, even in an unrelated joint, were also further medical risk factors for a further PJI. The use of non-steroidal anti-inflammatory drugs (NSAID) and antifungal drugs before arthroplasty also were risk factors (Table 1). Examples of the fitting of the Kaplan-Meier curves with the log-normal stepwise regression model showed a general good agreement (Figure 4).

# 7 Discussion

PJI is a relative rare but highly impactful outcome, both in terms of patients' quality of life and resources consumption,[3, 4, 19-22] of arthroplasty. Prevention or early identification of PJI through stringent medical follow-up is not a viable option because of the large population undergoing hip and knee arthroplasty and the potential long follow-up time, as shown here and in multiple studies PJI can be diagnosed even years after surgery.[2, 23, 24] Because of



For peer review only - http://bmjopen.bmj.com/site/about/guidelines.xhtml

the long follow-up required to capture the full extent of PJI, we chose a study period of 5 years and to allow patients to achieve such observation we restricted the last date of index surgery to 5 years before the last data collection in CPRD extraction analysed. In light of the administrative nature of the CPRD database, medical history was based on the presence of a specific diagnostic code, therefore the absence of such code in a patient records was assumed to represent absence of such event. On the contrary when a record was expected, such in case of BMI, primary or secondary arthroplasty along with alcohol and smoking status, the absence of any entry resulted in this patient having the covariate under consideration categorized as “unknown”.

Patients identified in CPRD AURUM were statistically different than those identified in CPRD GOLD (Table S4-S7) likely as a consequence of the different geographical coverage of England by the two databases associated to the population characteristics variation around the country in term of age and diseases.[25, 26] However, as expected the impact of almost all covariates on the risk of developing PJI was the same ($P > 0.05$) when estimated with the same parametric model. The hazard of PJI (Figure 1) exhibited a monotonically decreasing profile typical of other studies investigating such occurrence [20, 27-30] and, in both databases, the log-normal model returned the best fitting of the data as this model is suitable for this type of risk profile.[31] Similarly, the log-logistic, another model suitable for decreasing hazard,[32] also returned good fitting (Table S8 and Table S9).

The observed PJI incidence in this study (~0.7% after 5 years) is lower than that sometimes reported. One possible explanation for this, was the use of a specific ICD-10 code for the identification of PJI (T84.5) while in our studies reported higher PJI rates (about 2.5% over 5 years) additional ICD-10 codes were employed to identify PJI, for example T81.4 (Infection following a procedure) T84.7 (Infection and inflammatory reaction due to other internal orthopaedic prosthetic devices, implants and grafts)[33]; however such codes appear less

For peer review only



For peer review only - http://bmjopen.bmj.com/site/about/guidelines.xhtml

specific and, for example, T81.4 may lead to consider a superficial infection of the surgical wound as a PJI. while M00 corresponds to a diagnosis of pyogenic arthritis [5]. When using only the presence of ICD-10 code T84.5 the PJI incidence at 5 years observed in this study is similar the 0.89% previously reported. [34]

As regression coefficients in the risk equations obtained from both databases were not statistically different for all covariates included in this study, both cohorts were pooled to increase the population size and, consequently, reduce the error associated with the coefficients estimation.[35, 36] The regression model built using the pooled cohort performance was evaluated using the area under the receiver operator curve that represents the balance between sensitivity (correctly identified cases) and specificity (correctly identified non-cases). The model performance did not significantly vary when applied to the likelihood of developing PJI over different follow-up time, probability due to the unbalance nature of the cohort in terms of patients developing PJI and patients not developing PJI. The area under the precision-recall curve (Figure 3b) instead is not impacted by unbalanced samples and the model performed better with increasing follow-up time.[37]

Stepwise selection is a classic variable reduction technique and was employed here to simplify the model minimizing the loss of performance. Negative coefficients indicate an increased risk of PJI compared to the reference; analogously, positive coefficients indicate a reduced risk of PJI compared to the reference. The greater the absolute value of the coefficient the greater the impact of the risk of PJI. Most of the risk factors identified through this approach are in line with those commonly associated to PJI (Table 1) as male gender, increased BMI and a previous PJI in the same joint or in another joint were retained in the list of covariates after stepwise selection.[2, 9, 29, 38] Age is sometimes not reported as a risk factor for PJI [9, 39], however the protective role of aging on PJI observed in this study was also seen previously. [34] A possible explanation for such correlation is “survival bias” as



For peer review only - http://bmjopen.bmj.com/site/about/guidelines.xhtml

older patients dying at higher rates would be censored at the time of death before having the opportunity of developing PJI. Smoking is sometimes reported as a risk factor PJI [9, 40] because it causes delay in wound healing but sometimes the opposite is reported [41],as here, where patient's smoking status was not included in the final model. Moreover, the active role of numerous medications provides a range of modifiable risk factors that could also inform surgery scheduling when arthroplasty is an elective procedure as the risk of PJI after NSAID and antifungal use decrease with increasing interval between the last use and the arthroplasty. The plausibility of the role of NSAID in PJI could be linked the increased cardiovascular risk resulting from their use [42] that in turn increases the risk of PJI, it is also widely accepted the immunosuppression is a general risk for the development of PJI and such NSAID role in PJI can also be linked to the immunological activity [43]. The models did not identify the use of steroidal injection in the joint as a risk associated to PJI in line with the advice of American Academy of Orthopaedic Surgeons [44]. The use of antifungal drug prior to arthroplasty has not been generally investigated as risk factor for PJI; it is possible that a prescription of antifungal drugs before the joint replacement surgery is a risk factor in our model as this would indicate a patient more susceptible to infections.

Compared to other available models, beside the UK specific population, our model includes patients both with high BMI (generally considered more at risk of PJI) or unknown BMI, a previous risk scoring model excluded patients with either missing BMI information or BMI > 50 [5]. The model developed here considered a wider range of covariates compared to the Mayo Prosthetic Joint Infection Risk Score [45] that included only diabetes or a limited number [46]. Moreover, the model presented here is not specific for specific pathogen in contrast with a *Staphylococcus aureus* specific model [47]; this particular relevant in light of the multitude of pathogens isolated in PJI such Gram+, Gram- and fungi [2]. Additionally,

For peer review only

the risk equation presented here has also consideration of a patient drug history that was very limited, if at all, in the other published models.[5, 45-47].

The model performance after stepwise selection was still high as shown by the C-statistic (Table S11) and the visual fit of the curves (Figure 4) demonstrating how the retained covariates are those pivotal in determining a patients risk to develop PJI after arthroplasty. The model developed here does not simply provide a list of risk factors for PJI but enables an objective quantification of the risk to develop PJI after hip or knee arthroplasty. As most of the identified risks are not modifiable the application of this risk scoring algorithm would not primarily allow a personalized pre-surgery drug prescription consideration but will guide follow-up care pivoting the resources (visits, test etc..) to the subgroup of patients at higher risks of PJI. As in most retrospective studies there were numerous missing data, Missing data have been handled through categorical binning and no mechanisms related to missingness have been hypothesised (i.e. missing at random or missing not at random); in order to avoid selection bias, the analysis has not been restricted to complete cases; however the criteria requiring data at baseline for certain variables may invite bias, as the patient profile of those with complete data may be different to those without. Nevertheless, a workable dataset is required to fulfil the study objectives and, therefore, this practical approach was adopted to identify a suitable study population. Another possible limitation is that comorbidities were considered as binary (present or absent), without accounting for illness severity and only at baseline without accounting for time-varying factors. Despite these acknowledged limitations, the large population size, the quality of the data in the database and the numerous covariate considered are considerable strength of this work. Sensitivity analysis and multiple imputations could be further applied to refine the risk scoring algorithm presented.



For peer review only - http://bmjopen.bmj.com/site/about/guidelines.xhtml

## 8 Conclusions

Patients undergoing hip or knee arthroplasty present in CPRD AURUM and CPRD GOLD exhibit different sociodemographic characteristics and medical/drug history. However, the coefficients of parametric models, fitted to the survival curves were not statistically different allowing for pooling cohorts.

The model developed here demonstrated a good ability to identify patients at risk (based on C-statistic) and is based on the UK specific population. Such capability would allow the NHS to review the current guidelines, potentially devising a targeted program of medical treatment pre-surgery, when appropriate, and of close monitoring to a small fraction of the patients after undergoing hip or knee arthroplasty allowing to reduce the incidence and impact of PJI.

The newly developed risk equations will also provide contemporary inputs for the economic evaluation of new technologies for the prevention of PJI supporting health technology assessment (HTA) from The National Institute for Health and Care Excellence (NICE).

## 9 Data Availability

This study is based in part on data from the Clinical Practice Research Datalink obtained under license from the UK Medicines and Healthcare products Regulatory Agency. The data is provided by patients and collected by the NHS as part of their care and support. The interpretation and conclusions contained in this study are those of the author/s alone.

The authors did not have any special access privileges that others would not have. Other researchers can apply for access to the raw data at the following URL: https://cprd.com/data-access/.



For peer review only - http://bmjopen.bmj.com/site/about/guidelines.xhtml

## 10 Competing interests statement

The authors have not conflict of interest to declare in the relation to this work.

## 11 Authors contribution

SP and PP conceived the idea for the study. SP carried out the statistical analysis and wrote the first draft of the manuscript. PP supervised the study and analysis. All authors reviewed and revised the manuscript. PP acts as guarantor.

## 12 Funding

This work has also been supported by Wellcome Trust (WT109455MA). The funder had no role in the study design, data collection and analysis, decision to publish, or preparation of the manuscript.

## 13 Research Ethics Approval

This study involves human participants and was approved by an Ethics Committee(s) or Institutional Board(s). ISAC committee from CPRD study protocol (19_009).



For peer review only - http://bmjopen.bmj.com/site/about/guidelines.xhtml

# 14 References


1. Osmon, D.R., et al., *Diagnosis and management of prosthetic joint infection: clinical practice guidelines by the Infectious Diseases Society of America.* Clin Infect Dis, 2013. **56**(1): p. e1-e25.
2. Perni, S., B. Bojan, and P. Prokopovich, *A retrospective study of risk factors, causative micro-organisms and healthcare resources consumption associated with prosthetic joint infections (PJI) using the Clinical Practice Research Datalink (CPRD) Aurum database.* PLOS ONE, 2023. **18**(3): p. e0282709.
3. Moore, A.J., et al., *Deep prosthetic joint infection: a qualitative study of the impact on patients and their experiences of revision surgery.* BMJ Open, 2015. **5**(12): p. e009495.
4. Kallala R. F., et al., *Financial analysis of revision knee surgery based on NHS tariffs and hospital costs.* The Bone & Joint Journal, 2015. **97-B**(2): p. 197-201.
5. Bülow, E., et al., *Prediction of Early Periprosthetic Joint Infection After Total Hip Arthroplasty.* Clin Epidemiol, 2022. **14**: p. 239-253.
6. Kuo, F.-C., W.-H. Hu, and Y.-J. Hu, *Periprosthetic Joint Infection Prediction via Machine Learning: Comprehensible Personalized Decision Support for Diagnosis.* The Journal of Arthroplasty, 2022. **37**(1): p. 132-141.
7. Berbari, E., et al., *The Mayo Prosthetic Joint Infection Risk Score: Implication for Surgical Site Infection Reporting and Risk Stratification.* Infection control and hospital epidemiology : the official journal of the Society of Hospital Epidemiologists of America, 2012. **33**: p. 774-81.
8. Klemt, C., et al., *The Use of Artificial Intelligence for the Prediction of Periprosthetic Joint Infection Following Aseptic Revision Total Knee Arthroplasty.* J Knee Surg, 2023(EFirst).
9. Kunutsor, S.K., et al., *Systematic review of risk prediction scores for surgical site infection or periprosthetic joint infection following joint arthroplasty.* Epidemiology & Infection, 2017. **145**(9): p. 1738-1749.
10. Taur, S.R., *Observational designs for real-world evidence studies.* Perspect Clin Res, 2022. **13**(1): p. 12-16.
11. Rassen, J.A., et al., *High-dimensional propensity scores for empirical covariate selection in secondary database studies: Planning, implementation, and reporting.* Pharmacoepidemiology and Drug Safety, 2023. **32**(2): p. 93-106.
12. Wolf, A., et al., *Data resource profile: Clinical Practice Research Datalink (CPRD) Aurum.* International Journal of Epidemiology, 2019. **48**(6): p. 1740-1740g.
13. Jick, S., et al., *Use of the CPRD Aurum Database: Insights Gained from New Data Quality Assessments.* Clin Epidemiol, 2023. **15**: p. 1219-1222.
14. CPRD. *Primary care data for public health research.* 15 March 2023]; Available from: https://cprd.com/primary-care-data-public-health-research.
15. Herrett, E., et al., *Data Resource Profile: Clinical Practice Research Datalink (CPRD).* International Journal of Epidemiology, 2015. **44**(3): p. 827-836.
16. Barzi, F., et al., *Cardiovascular risk prediction tools for populations in Asia.* J Epidemiol Community Health, 2007. **61**(2): p. 115-21.
17. R Core Team, *R: A language and environment for statistical computing.*, in *R Foundation for Statistical Computing.* 2019: Vienna, Austria.
18. Therneau T, *A Package for Survival Analysis in S. version 2.38.* 2015.
19. Bozic, K.J., et al., *Comparative Epidemiology of Revision Arthroplasty: Failed THA Poses Greater Clinical and Economic Burdens Than Failed TKA.* Clin Orthop Relat Res, 2015. **473**(6): p. 2131-8.
20. Kurtz, S.M., et al., *Prosthetic joint infection risk after TKA in the Medicare population.* Clin Orthop Relat Res, 2010. **468**(1): p. 52-6.
21. Li, T., et al., *Risk factors associated with surgical site infections following joint replacement surgery: a narrative review.* Arthroplasty, 2022. **4**(1): p. 11.
22. Garfield, K., et al., *What are the inpatient and day case costs following primary total hip replacement of patients treated for prosthetic joint infection: a matched cohort study using*




For peer review only - http://bmjopen.bmj.com/site/about/guidelines.xhtml

*linked data from the National Joint Registry and Hospital Episode Statistics.* BMC medicine, 2020. **18**(1): p. 335-335.
23. Tande, A.J. and R. Patel, *Prosthetic joint infection.* Clin Microbiol Rev, 2014. **27**(2): p. 302-45.
24. Huotari, K., M. Peltola, and E. Jämsen, *The incidence of late prosthetic joint infections: a registry-based study of 112,708 primary hip and knee replacements.* Acta Orthop, 2015. **86**(3): p. 321-5.
25. NHS Digital. *Patients Registered at GP practices in England as at 1 March 2023*. 2023; Available from: https://digital.nhs.uk/data-and-information/publications/statistical/patients-registered-at-a-gp-practice/march-2023.
26. NHS Digital. *CCG Outcomes Indicator Set - October 2022*. 2022; Available from: https://digital.nhs.uk/data-and-information/publications/statistical/ccg-outcomes-indicator-set/october-2022.
27. Iannotti, F., et al., *Prevention of Periprosthetic Joint Infection (PJI): A Clinical Practice Protocol in High-Risk Patients.* Trop Med Infect Dis, 2020. **5**(4).
28. Izakovicova, P., O. Borens, and A. Trampuz, *Periprosthetic joint infection: current concepts and outlook.* EFORT Open Rev, 2019. **4**(7): p. 482-494.
29. Lenguerrand, E., et al., *Description of the rates, trends and surgical burden associated with revision for prosthetic joint infection following primary and revision knee replacements in England and Wales: an analysis of the National Joint Registry for England, Wales, Northern Ireland and the Isle of Man.* BMJ Open, 2017. **7**(7): p. e014056.
30. Pulido, L., et al., *Periprosthetic joint infection: the incidence, timing, and predisposing factors.* Clinical orthopaedics and related research, 2008. **466**(7): p. 1710-1715.
31. Gupta, R.C., N. Kannan, and A. Raychaudhuri, *Analysis of lognormal survival data.* Mathematical Biosciences, 1997. **139**(2): p. 103-115.
32. Bennett, S., *Log-Logistic Regression Models for Survival Data.* Journal of the Royal Statistical Society Series C: Applied Statistics, 2018. **32**(2): p. 165-171.
33. Weinstein, E.J., et al., *Incidence, Microbiological Studies, and Factors Associated With Prosthetic Joint Infection After Total Knee Arthroplasty.* JAMA Network Open, 2023. **6**(10): p. e2340457-e2340457.
34. The McMaster Arthroplasty Collaborative, *Risk Factors for Periprosthetic Joint Infection Following Primary Total Hip Arthroplasty: A 15-Year, Population-Based Cohort Study.* JBJS, 2020. **102**(6): p. 503-509.
35. Khera, R., et al., *Performance of the Pooled Cohort Equations to Estimate Atherosclerotic Cardiovascular Disease Risk by Body Mass Index.* JAMA Network Open, 2020. **3**(10): p. e2023242-e2023242.
36. Vasan, R.S. and E. van den Heuvel, *Differences in estimates for 10-year risk of cardiovascular disease in Black versus White individuals with identical risk factor profiles using pooled cohort equations: an in silico cohort study.* The Lancet Digital Health, 2022. **4**(1): p. e55-e63.
37. Max Kuhn and K. Johnson, *Applied Predictive Modeling*. 2013: Springer New York, NY.
38. Lenguerrand, E., et al., *Risk factors associated with revision for prosthetic joint infection after hip replacement: a prospective observational cohort study.* The Lancet Infectious Diseases, 2018. **18**(9): p. 1004-1014.
39. Inoue, D., et al., *Age alone is not a risk factor for periprosthetic joint infection.* Journal of Hospital Infection, 2019. **103**(1): p. 64-68.
40. Bojan, B., S. Perni, and P. Prokopovich, *Systematic Review and Meta-Analysis of Tobacco Use as a Risk Factor for Prosthetic Joint Infection After Total Hip Replacement.* Arthroplast Today, 2020. **6**(4): p. 959-971.
41. Ren, X., et al., *Patients' risk factors for periprosthetic joint infection in primary total hip arthroplasty: a meta-analysis of 40 studies.* BMC Musculoskeletal Disorders, 2021. **22**(1): p. 776.
42. Varga, Z., S.R.A. Sabzwari, and V. Vargova, *Cardiovascular Risk of Nonsteroidal Anti-Inflammatory Drugs: An Under-Recognized Public Health Issue.* Cureus, 2017. **9**(4): p. e1144.



For peer review only - http://bmjopen.bmj.com/site/about/guidelines.xhtml

43. Cho, J.Y., *Immunomodulatory effect of nonsteroidal anti-inflammatory drugs (NSAIDs) at the clinically available doses.* Arch Pharm Res, 2007. **30**(1): p. 64-74.
44. American Academy of Orthopaedic Surgeons, *Diagnosis and Prevention of Periprosthetic Joint Infections - Clinical Practice Guideline.* 2019.
45. Berbari, E.F., et al., *The Mayo prosthetic joint infection risk score: implication for surgical site infection reporting and risk stratification.* Infect Control Hosp Epidemiol, 2012. **33**(8): p. 774-81.
46. Tan, T.L., et al., *Development and Evaluation of a Preoperative Risk Calculator for Periprosthetic Joint Infection Following Total Joint Arthroplasty.* JBJS, 2018. **100**(9): p. 777-785.
47. Espindola, R., et al., *Preoperative and perioperative risk factors, and risk score development for prosthetic joint infection due to Staphylococcus aureus: a multinational matched case-control study.* Clin Microbiol Infect, 2022. **28**(10): p. 1359-1366.


For peer review only

# 15 Figure legends

**Figure 1.** Examples of unadjusted Kaplan-Meier curves of cumulative risk of PJI (and 95% confidence intervals) for the entire cohorts (a) and stratified based on gender (b) and history of diabetes at baseline (c). Geographical distribution of PJI rates in the CPRD regions of England (d)

**Figure 2.** Coefficients for time to PJI multivariate regression with log-normal model for data from CPRD GOLD (——) and AURUM database (——).AC Active cancer; AF Atrial Fibrillation; BMI Body mass index; CKD Chronic kidney disease; COPD Chronic obstructive pulmonary disease; DMRAD Disease modifying rheumatoid arthritis drug; DOAC Direct oral anticoagulant; DVT Deep vein thrombosis; HF Heart failure; IHD Ischaemic heart disease; MI Myocardial infarction; MTX Methotrexate; NSAID Non-steroidal anti-inflammatory drug; PE Pulmonary embolism; OA Osteoarthritis; RA Rheumatoid arthritis

**Figure 3.** Time dependent (—— 1 year —— 2 years —— 3 years —— 4 years —— 5 years) Receiver operating curve (ROC) (a) and Precision-Recall curves (b) of pooled log-normal equation.

**Figure 4.** Examples of Kaplan-Meier curves of risk of PJI and prediction using pooled log-normal parametric model after stepwise selection for both AURUM and GOLD CPRD database (a) gender and history of (b) Atrial Fibrillation, (c) Osteo-arthritis or (d) hypertension at baseline.

For peer review only

**Table 1.** Coefficients and SE of pooled lognormal parametric model of time to PJI after stepwise regression.

| Variable | Coefficient | SE |
|---|---|---|
| **Log(Scale)** | 1.644 | 0.025 |
| **Intercept** | 20.724 | 0.657 |
| **Male** | -0.827 | 0.111 |
| **Age** | | |
| ≤ 45 | Reference | |
| 46 – 55 | 1.036 | 0.37 |
| 56 – 65 | 1.469 | 0.343 |
| 66 – 75 | 1.823 | 0.341 |
| 76 – 85 | 1.917 | 0.349 |
| > 85 | 3.388 | 0.452 |
| **BMI** | | |
| ≤ 20 | Reference | |
| 20 – 25 | -0.462 | 0.477 |
| 26 – 30 | -0.424 | 0.467 |
| 31 – 35 | -0.812 | 0.471 |
| 36 – 50 | -1.257 | 0.478 |
| > 50 | -1.803 | 0.886 |
| Unknown | -1.15 | 0.461 |
| **Primary arthroplasty** | | |
| Yes | Reference | |
| No | -2.558 | 0.158 |
| Unknown | -2.467 | 0.606 |
| **Admission type** | | |
| Elective | Reference | |
| A&E | -1.064 | 0.185 |
| Unknown | 0.295 | 0.976 |
| **Patella resurfacing** | | |
| No | Reference | |
| Yes | 0.393 | 0.409 |
| N/A | 0.714 | 0.118 |
| **AF** | -0.484 | 0.193 |
| **DVT** | -0.392 | 0.23 |
| **Diabetes** | -0.357 | 0.148 |
| **OA** | -0.237 | 0.125 |
| **MI** | -0.329 | 0.182 |
| **Hypertension** | -0.312 | 0.105 |
| **PJI before in same joint** | -2.585 | 0.302 |
| **PJI before in another joint** | -1.818 | 0.257 |
| **NSAID** | | |
| No Use | Reference | |

For peer review only

| | | |
|---|---|---|
| Use < 3 months | -0.768 | 0.138 |
| Use 3 - 6 months | -0.589 | 0.22 |
| Use > 6 months | 0.112 | 0.14 |
| **Antifungal** | | |
| No Use | Reference | |
| Use < 3 months | -0.725 | 0.315 |
| Use 3 - 6 months | -0.096 | 0.457 |
| Use > 6 months | -0.585 | 0.161 |

For peer review only

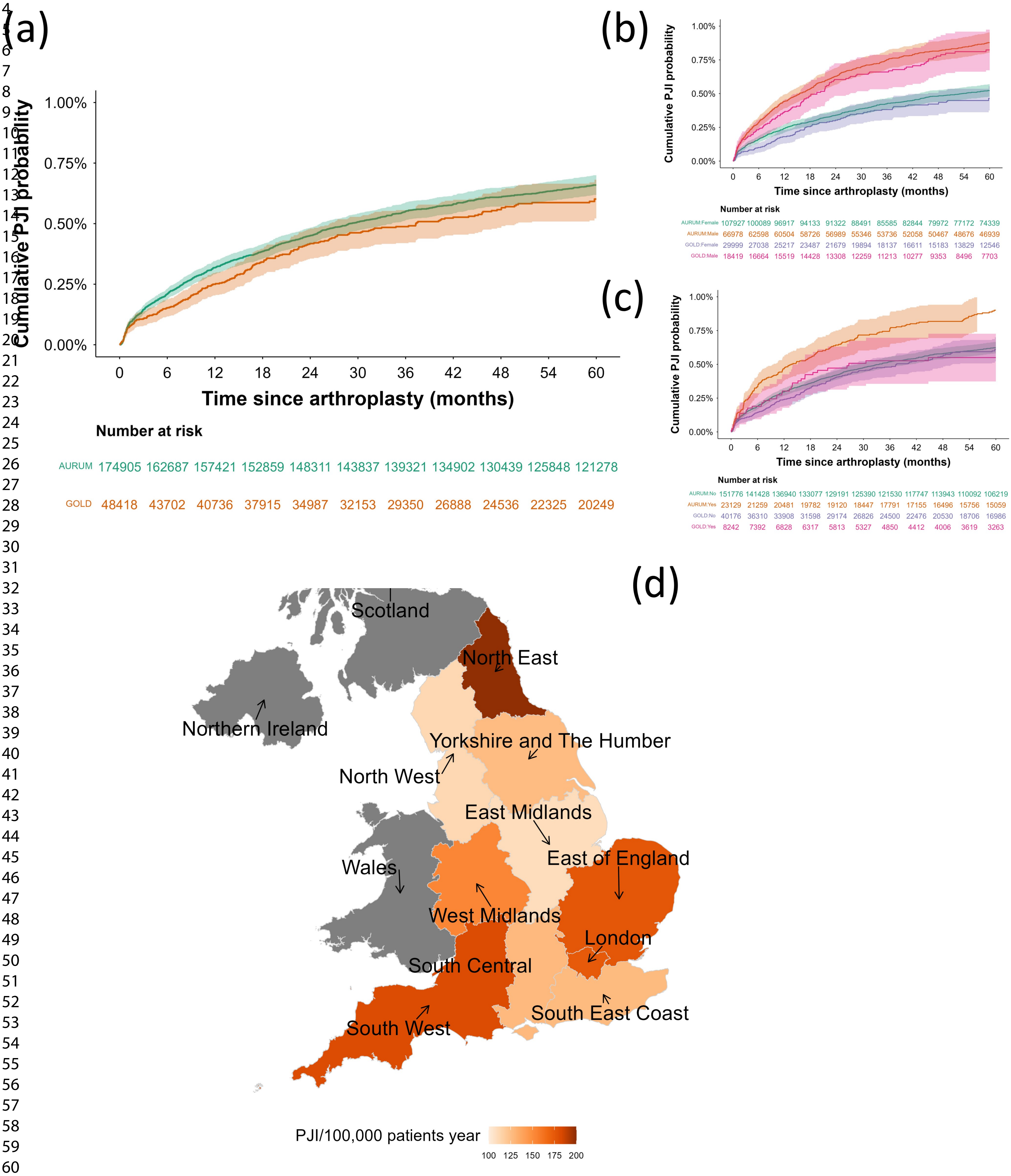
(a)
Cumulative PJI probability
1.00%
0.75%
0.50%
0.25%
0.00%
0 6 12 18 24 30 36 42 48 54 60
Time since arthroplasty (months)
Number at risk
AURUM 174905 162687 157421 152859 148311 143837 139321 134902 130439 125848 121278
GOLD 48418 43702 40736 37915 34987 32153 29350 26888 24536 22325 20249
(b)
Cumulative PJI probability
1.00%
0.75%
0.50%
0.25%
0.00%
0 6 12 18 24 30 36 42 48 54 60
Time since arthroplasty (months)
Number at risk
AURUM:Female 107927 100089 96917 94133 91322 88491 85585 82844 79972 77172 74339
AURUM:Male 66978 62598 60504 58726 56989 55346 53736 52058 50467 48676 46939
GOLD:Female 29999 27038 25217 23487 21679 19894 18137 16611 15183 13829 12546
GOLD:Male 18419 16664 15519 14428 13308 12259 11213 10277 9353 8496 7703
(c)
Cumulative PJI probability
1.00%
0.75%
0.50%
0.25%
0.00%
0 6 12 18 24 30 36 42 48 54 60
Time since arthroplasty (months)
Number at risk
AURUM:No 151776 141428 136940 133077 129191 125390 121530 117747 113943 110092 106219
AURUM:Yes 23129 21259 20481 19782 19120 18447 17791 17155 16496 15756 15059
GOLD:No 40176 36310 33908 31598 29174 26826 24500 22476 20530 18706 16986
GOLD:Yes 8242 7392 6828 6317 5813 5327 4850 4412 4006 3619 3263
(d)
Scotland
North East
Northern Ireland
Yorkshire and The Humber
North West
East Midlands
Wales
East of England
West Midlands
London
South Central
South East Coast
South West
PJI/100,000 patients year
100 125 150 175 200

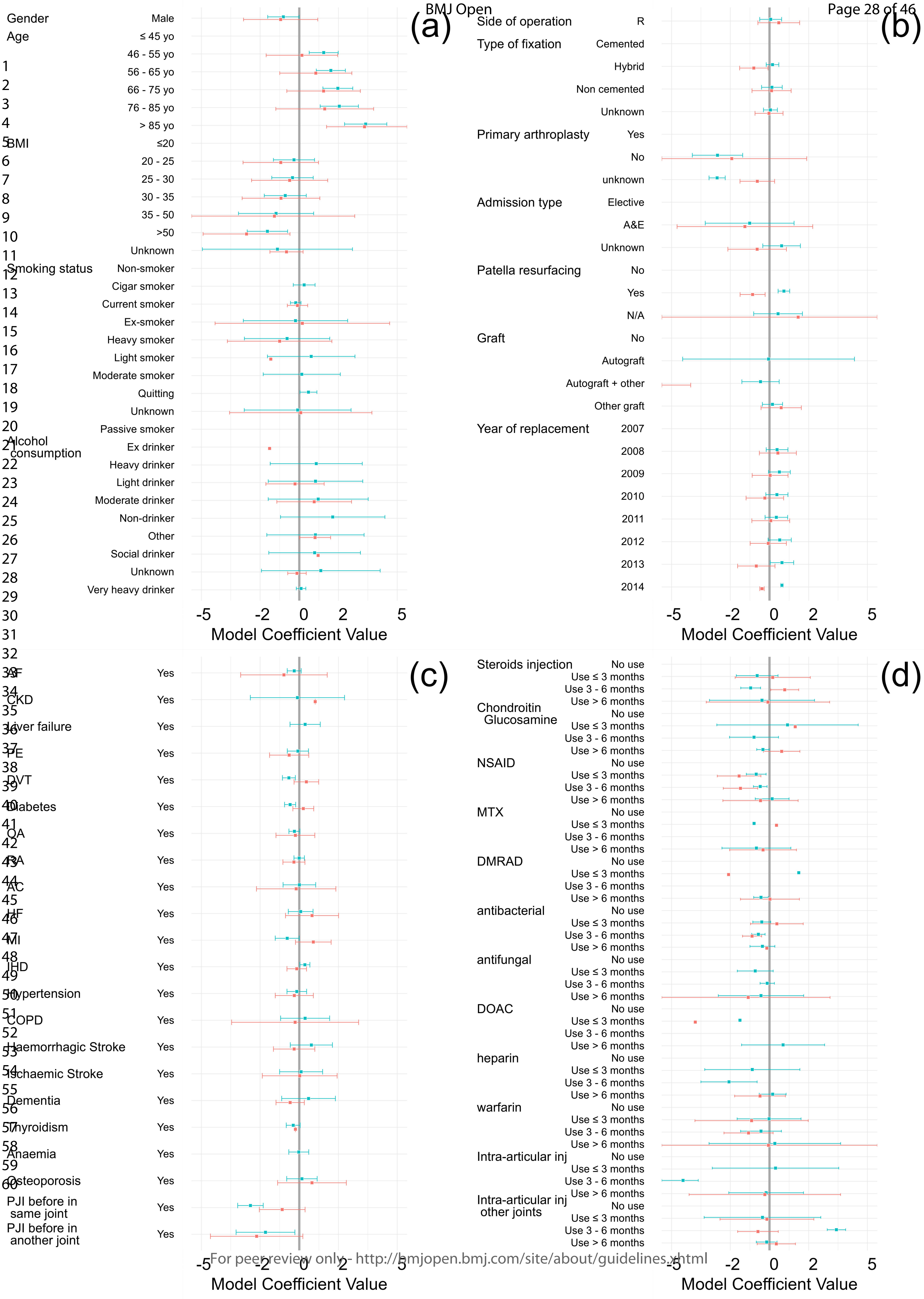
(a)
Gender
Male
Age
≤ 45 yo
46 - 55 yo
56 - 65 yo
66 - 75 yo
76 - 85 yo
> 85 yo
BMI
≤20
20 - 25
25 - 30
30 - 35
35 - 50
>50
Unknown
Smoking status
Non-smoker
Cigar smoker
Current smoker
Ex-smoker
Heavy smoker
Light smoker
Moderate smoker
Quitting
Unknown
Passive smoker
Alcohol consumption
Ex drinker
Heavy drinker
Light drinker
Moderate drinker
Non-drinker
Other
Social drinker
Unknown
Very heavy drinker
-5
-2
0
2
5
Model Coefficient Value
(b)
Side of operation
R
Type of fixation
Cemented
Hybrid
Non cemented
Unknown
Primary arthroplasty
Yes
No
unknown
Admission type
Elective
A&E
Unknown
Patella resurfacing
No
Yes
N/A
Graft
No
Autograft
Autograft + other
Other graft
Year of replacement
2007
2008
2009
2010
2011
2012
2013
2014
-5
-2
0
2
5
Model Coefficient Value
(c)
AF
Yes
CKD
Yes
Liver failure
Yes
PE
Yes
DVT
Yes
Diabetes
Yes
OA
Yes
RA
Yes
AC
Yes
HF
Yes
MI
Yes
IHD
Yes
Hypertension
Yes
COPD
Yes
Haemorrhagic Stroke
Yes
Ischaemic Stroke
Yes
Dementia
Yes
Thyroidism
Yes
Anaemia
Yes
Osteoporosis
Yes
PJI before in same joint
Yes
PJI before in another joint
Yes
-5
-2
0
2
5
Model Coefficient Value
(d)
Steroids injection
No use
Use ≤ 3 months
Use 3 - 6 months
Use > 6 months
Chondroitin Glucosamine
No use
Use ≤ 3 months
Use 3 - 6 months
Use > 6 months
NSAID
No use
Use ≤ 3 months
Use 3 - 6 months
Use > 6 months
MTX
No use
Use ≤ 3 months
Use 3 - 6 months
Use > 6 months
DMRAD
No use
Use ≤ 3 months
Use 3 - 6 months
Use > 6 months
antibacterial
No use
Use ≤ 3 months
Use 3 - 6 months
Use > 6 months
antifungal
No use
Use ≤ 3 months
Use 3 - 6 months
Use > 6 months
DOAC
No use
Use ≤ 3 months
Use 3 - 6 months
Use > 6 months
heparin
No use
Use ≤ 3 months
Use 3 - 6 months
Use > 6 months
warfarin
No use
Use ≤ 3 months
Use 3 - 6 months
Use > 6 months
Intra-articular inj
No use
Use ≤ 3 months
Use 3 - 6 months
Use > 6 months
Intra-articular inj other joints
No use
Use ≤ 3 months
Use 3 - 6 months
Use > 6 months
-5
-2
0
2
5
Model Coefficient Value

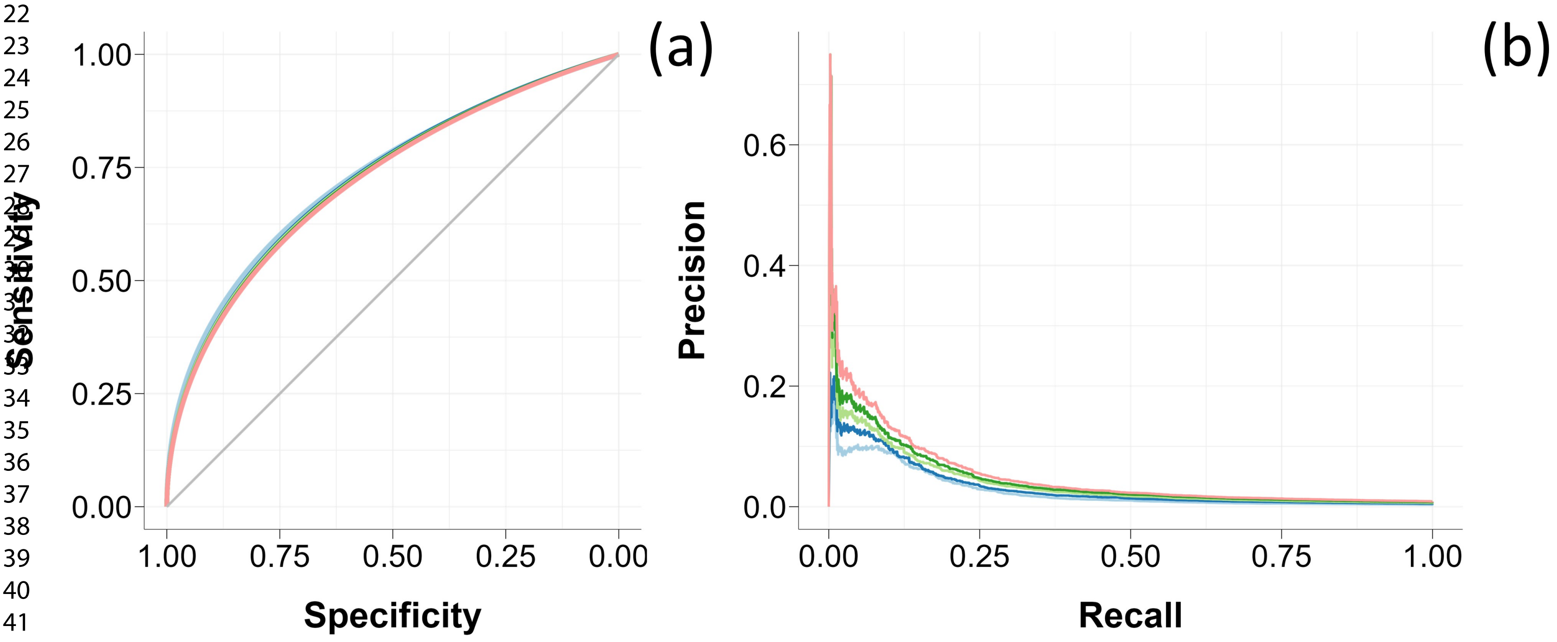
(a)
Sensitivity
1.00
0.75
0.50
0.25
0.00
Specificity
(b)
Precision
0.6
0.4
0.2
0.0
0.00
0.25
0.50
0.75
1.00
Recall

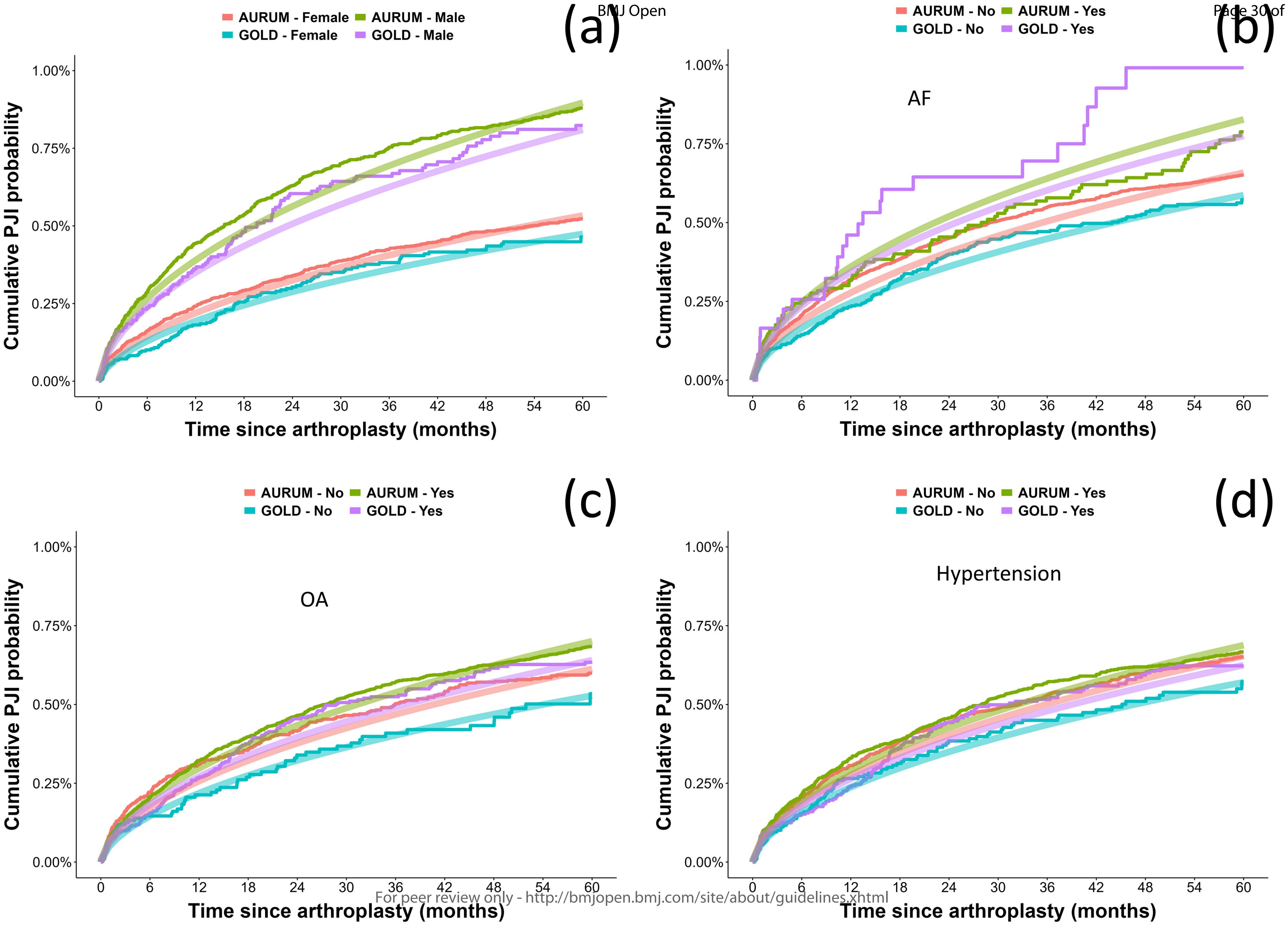
(a)
AURUM - Female
AURUM - Male
GOLD - Female
GOLD - Male
Cumulative PJI probability
1.00%
0.75%
0.50%
0.25%
0.00%
0 6 12 18 24 30 36 42 48 54 60
Time since arthroplasty (months)
(b)
AURUM - No
AURUM - Yes
GOLD - No
GOLD - Yes
AF
Cumulative PJI probability
Time since arthroplasty (months)
(c)
AURUM - No
AURUM - Yes
GOLD - No
GOLD - Yes
OA
Cumulative PJI probability
Time since arthroplasty (months)
(d)
AURUM - No
AURUM - Yes
GOLD - No
GOLD - Yes
Hypertension
Cumulative PJI probability
Time since arthroplasty (months)

# Retrospective study in UK using the Clinical Practice Research Datalink (CPRD) AURUM and GOLD database for the determination of risk equations for prosthetic joint infections (PJI)

*Stefano Perni[1] and Polina Prokopovich[1,*]*

School of Pharmacy and Pharmaceutical Sciences, Cardiff University, Cardiff, UK

# Supplementary Material

For peer review only

**Table S 1.** Flow of patients through initial data extraction.

| | AURUM | | GOLD | |
|---|---|---|---|---|
| | Number of joints | Number of patients | Number of joints | Number of patients |
| Knee or hip replacement in database | 330,173 | 235,249 | 88,458 | 62,672 |
| Over 31 years old at index date | 329,720 | 235,071 | 88,268 | 62,584 |
| Arthroplasty surgery after 2007 | 288,124 | 223,450 | 71,467 | 54,888 |
| With minimum 6 months lookback | 284,048 | 222,060 | 71,467 | 54,888 |
| After removing implausible data | 283,789 | 221,826 | 71,072 | 54,833 |
| Surgery before 2015 | 174,905 | 144,048 | 48,419 | 39,764 |

For peer review only

**Table S 2.** Number of joints not compleating the observation study period (5 years) and reason for loss during follow-up.

| Reason for not completing study period | AURUM | | GOLD | |
|---|---|---|---|---|
| | n | Frequency | n | Frequency |
| Developing PJI | 1,021 | 0.58% | 228 | 0.47% |
| Death | 25,657 | 14.67% | 6,021 | 12.44% |
| Last collection date before end of observation period | 749 | 0.43% | 16,604 | 34.29% |
| Device replaced for any reason | 4,108 | 2.35% | 1,090 | 2.25% |
| Transferred out of CPRD | 22,586 | 12.91% | 4,323 | 8.93% |

**Table S 3.** Follow-up (days) duration in patients not completed study period (5 years).

| | AURUM | GOLD |
|---|---|---|
| Mean (SD) | 769.61 (578.90) | 798.96 (538.44) |
| Median | 726 | 768 |
| IQR | 197 – 1,281 | 316 – 1,246 |

**Table S 4.** Characteristics of patients at the time of hip or knee implant surgery.

| Variable | All | AURUM | GOLD |
|---|---|---|---|
| **Gender** | | | |
| Female | 137,926 (61.76%) | 107,927 (61.71 %) | 29,999 (61.96 %) |
| Male | 85,398 (38.24%) | 66,979 (38.29 %) | 18,419 (38.04 %) |
| **Age** | | | |
| ≤ 45 | 3,221 (1.44%) | 2,593 (1.48 %) | 628 (1.30 %) |
| 46 – 55 | 13,170 (5.90%) | 10,332 (5.91 %) | 2,838 (5.86 %) |
| 56 – 65 | 43,379 (19.42%) | 34,012 (19.45 %) | 9,367 (19.35 %) |
| 66 – 75 | 75,181 (33.66%) | 58,764 (33.60 %) | 16,417 (33.91 %) |
| 76 – 85 | 65,288 (29.23%) | 51,157 (29.25 %) | 14,131 (29.19 %) |
| > 85 | 23,085 (10.34%) | 18,048 (10.32 %) | 5,037 (10.40 %) |
| Mean (SD) | 71.98 | 71.97 (10.97) | 72.03 (10.88) |
| Median | 73 | 73 | 73 |
| IQR | 65.00 - 80.00 | 65.00 - 80.00 | 65.00 - 80.00 |
| Min, max | 31.00, 109.00 | 31.00, 109.00 | 31.00, 107.00 |
| **BMI** | | | |
| ≤ 20 | 6,364 (2.85%) | 4,111 (2.35 %) | 2,253 (4.65 %) |
| 20 – 25 | 31,896 (14.28%) | 21,173 (12.11 %) | 10,723 (22.15 %) |
| 26 – 30 | 55,729 (24.95%) | 39,077 (22.34 %) | 16,652 (34.39 %) |
| 31 – 35 | 38,395 (17.19%) | 27,950 (15.98 %) | 10,445 (21.57 %) |
| 36 – 50 | 23,629 (10.58%) | 17,849 (10.20 %) | 5,780 (11.94 %) |
| > 50 | 540 (0.24%) | 472 (0.27 %) | 68 (0.14 %) |
| Unknown | 66,771 (29.90%) | 64,274 (36.75 %) | 2,497 (5.16 %) |
| Mean (SD) | 29.22 | 29.52 (6.12) | 28.52 (5.74) |
| Median | 28.6 | 28.9 | 28 |
| IQR | 25.10 - 32.70 | 25.40 - 33.00 | 24.60 - 31.90 |
| Min, max | 10.46, 73.00 | 10.46, 73.00 | 14.50, 67.50 |
| **Smoking status** | | | |
| Non-smoker | 122,373 (54.80%) | 95,096 (54.37 %) | 27,277 (56.34 %) |
| Cigar | 165 (0.07%) | 165 (0.09 %) | 0 (0.00 %) |
| Current | 12,227 (5.48%) | 8,080 (4.62 %) | 4,147 (8.56 %) |
| Ex-smoker | 70,667 (31.64%) | 54,288 (31.04 %) | 16,379 (33.83 %) |
| Heavy | 298 (0.13%) | 230 (0.13 %) | 68 (0.14 %) |
| Light | 772 (0.35%) | 573 (0.33 %) | 199 (0.41 %) |
| Moderate | 496 (0.22%) | 496 (0.28 %) | 0 (0.00 %) |
| Quitting | 674 (0.30%) | 674 (0.39 %) | 0 (0.00 %) |
| Passive smoker | 15 (0.01%) | 0 (0.00 %) | 15 (0.03 %) |
| Unknown | 15,637 (7.00%) | 15,304 (8.75 %) | 333 (0.69 %) |
| **Alcohol consumption** | | | |
| Non-drinker | 8,628 (3.86%) | 3,266 (1.87 %) | 5,362 (11.07 %) |
| Ex drinker | 384 (0.17%) | 270 (0.15 %) | 114 (0.24 %) |
| Heavy drinker | 959 (0.43%) | 899 (0.51 %) | 60 (0.12 %) |

For peer review only

| | | | |
|---|---|---|---|
| Light drinker | 40,588 (18.17%) | 32,762 (18.73 %) | 7,826 (16.16 %) |
| Moderate drinker | 7,284 (3.26%) | 5,416 (3.10 %) | 1,868 (3.86 %) |
| Social drinker | 4,080 (1.83%) | 2,891 (1.65 %) | 1,189 (2.46 %) |
| Very heavy | 690 (0.31%) | 338 (0.19 %) | 352 (0.73 %) |
| Other | 1,546 (0.69%) | 1,080 (0.62 %) | 466 (0.96 %) |
| Unknown | 159,165 (71.27%) | 127,984 (73.17 %) | 31,181 (64.40 %) |

For peer review only

**Table S 5.** Characteristics of hip or knee implant surgery.

| Variable | All | AURUM | GOLD |
|---|---|---|---|
| **Joint replaced** | | | |
| Femur | 31,766 (14.22%) | 24,963 (14.27 %) | 6,803 (14.05 %) |
| Hip | 96,267 (43.11%) | 75,439 (43.13 %) | 20,828 (43.02 %) |
| Knee | 95,291 (42.67%) | 74,504 (42.60 %) | 20,787 (42.93 %) |
| **Laterality of operation** | | | |
| Left | 104,858 (46.95%) | 82,039 (46.90 %) | 22,819 (47.13 %) |
| Right | 118,466 (53.05%) | 92,867 (53.10 %) | 25,599 (52.87 %) |
| **Fixation method** | | | |
| Cemented | 145,726 (65.25%) | 113,248 (64.75 %) | 32,478 (67.08 %) |
| Hybrid | 13,399 (6.00%) | 10,447 (5.97 %) | 2,952 (6.10 %) |
| Non cemented | 53,836 (24.11%) | 43,256 (24.73 %) | 10,580 (21.85 %) |
| Unknown | 10,363 (4.64%) | 7,955 (4.55 %) | 2,408 (4.97 %) |
| **Primary arthroplasty** | | | |
| Yes | 205,321 (91.94%) | 160,883 (91.98 %) | 44,438 (91.78 %) |
| No | 17,226 (7.71%) | 13,404 (7.66 %) | 3,822 (7.89 %) |
| Unknown | 777 (0.35%) | 619 (0.35 %) | 158 (0.33 %) |
| **Admission type** | | | |
| Elective | 184,138 (82.45%) | 144,145 (82.41 %) | 39,993 (82.60 %) |
| A&E | 38,494 (17.24%) | 30,270 (17.31 %) | 8,224 (16.99 %) |
| Unknown | 692 (0.31%) | 491 (0.28 %) | 201 (0.42 %) |
| **Patella resurfacing (* only for knee replacement surgeries)** | | | |
| No | 91,375 (40.92%) | 71,166 (40.69 %) | 20,209 (41.74 %) |
| Yes | 3,916 (1.75%) | 3,338 (1.91 %) | 578 (1.19 %) |
| **Graft** | | | |
| No | 219,645 (98.35%) | 171,999 (98.34 %) | 47,646 (98.41 %) |
| Autograft | 1,673 (0.75%) | 1,331 (0.76 %) | 342 (0.71 %) |
| Autograft + other | 67 (0.03%) | 58 (0.03 %) | 9 (0.02 %) |
| Other graft | 1,939 (0.87%) | 1,518 (0.87 %) | 421 (0.87 %) |
| **Replacement year** | | | |
| 2007 | 24,539 (10.99%) | 18,787 (10.74 %) | 5,752 (11.88 %) |
| 2008 | 26,289 (11.77%) | 20,182 (11.54 %) | 6,107 (12.61 %) |
| 2009 | 26,426 (11.83%) | 20,507 (11.72 %) | 5,919 (12.22 %) |
| 2010 | 28,061 (12.57%) | 21,742 (12.43 %) | 6,319 (13.05 %) |
| 2011 | 28,433 (12.73%) | 22,180 (12.68 %) | 6,253 (12.91 %) |
| 2012 | 29,069 (13.02%) | 22,961 (13.13 %) | 6,108 (12.62 %) |
| 2013 | 29,718 (13.31%) | 23,753 (13.58 %) | 5,965 (12.32 %) |
| 2014 | 30,788 (13.79%) | 24,793 (14.18 %) | 5,995 (12.38 %) |

For peer review only

**Table S 6.** Medical history of patients at the time of hip or knee implant surgery.

| Variable | All | AURUM | GOLD |
|---|---|---|---|
| **Atrial Fibrillation** | | | |
| No | 204,588 (91.61%) | 159,979 (91.47 %) | 44,609 (92.13 %) |
| Yes | 18,736 (8.39%) | 14,927 (8.53 %) | 3,809 (7.87 %) |
| **Liver failure** | | | |
| No | 222,841 (99.78%) | 174,529 (99.78 %) | 48,312 (99.78 %) |
| Yes | 483 (0.22%) | 377 (0.22 %) | 106 (0.22 %) |
| **CKD** | | | |
| No | 193,781 (86.77%) | 146,389 (83.70 %) | 47,392 (97.88 %) |
| Yes | 29,543 (13.23%) | 28,517 (16.30 %) | 1,026 (2.12 %) |
| **Pulmonary Embolism** | | | |
| No | 218,350 (97.77%) | 171,043 (97.79 %) | 47,307 (97.71 %) |
| Yes | 4,974 (2.23%) | 3,863 (2.21 %) | 1,111 (2.29 %) |
| **Deep Vein Thrombosis** | | | |
| No | 213,096 (95.42%) | 167,939 (96.02 %) | 45,157 (93.26 %) |
| Yes | 10,228 (4.58%) | 6,967 (3.98 %) | 3,261 (6.74 %) |
| **Diabetes** | | | |
| No | 191,953 (85.95%) | 151,777 (86.78 %) | 40,176 (82.98 %) |
| Yes | 31,371 (14.05%) | 23,129 (13.22 %) | 8,242 (17.02 %) |
| **Osteoarthritis** | | | |
| No | 72,266 (32.36%) | 55,705 (31.85 %) | 16,561 (34.20 %) |
| Yes | 151,058 (67.64%) | 119,201 (68.15 %) | 31,857 (65.80 %) |
| **Rheumatoid Arthritis** | | | |
| No | 175,330 (78.51%) | 130,011 (74.33 %) | 45,319 (93.60 %) |
| Yes | 47,994 (21.49%) | 44,895 (25.67 %) | 3,099 (6.40 %) |
| **Active Cancer** | | | |
| No | 150,190 (67.25%) | 113,646 (64.98 %) | 36,544 (75.48 %) |
| Yes | 73,134 (32.75%) | 61,260 (35.02 %) | 11,874 (24.52 %) |
| **Heart Failure** | | | |
| No | 218,251 (97.73%) | 170,804 (97.65 %) | 47,447 (97.99 %) |
| Yes | 5,073 (2.27%) | 4,102 (2.35 %) | 971 (2.01 %) |
| **Myocardial Infarction** | | | |
| No | 202,834 (90.82%) | 156,757 (89.62 %) | 46,077 (95.17 %) |
| Yes | 20,490 (9.18%) | 18,149 (10.38 %) | 2,341 (4.83 %) |
| **Ischaemic Heart Disease** | | | |
| No | 198,515 (88.89%) | 154,504 (88.34 %) | 44,011 (90.90 %) |
| Yes | 24,809 (11.11%) | 20,402 (11.66 %) | 4,407 (9.10 %) |
| **Hypertension** | | | |
| No | 99,688 (44.64%) | 79,303 (45.34 %) | 20,385 (42.10 %) |
| Yes | 123,636 (55.36%) | 95,603 (54.66 %) | 28,033 (57.90 %) |
| **COPD** | | | |
| No | 207,899 (93.09%) | 162,676 (93.01 %) | 45,223 (93.40 %) |

For peer review only

| | | | |
|---|---|---|---|
| Yes | 15,425 (6.91%) | 12,230 (6.99 %) | 3,195 (6.60 %) |
| **Haemorrhagic stroke** | | | |
| No | 219,436 (98.26%) | 171,293 (97.93 %) | 48,143 (99.43 %) |
| Yes | 3,888 (1.74%) | 3,613 (2.07 %) | 275 (0.57 %) |
| **Ischemic stroke** | | | |
| No | 215,807 (96.63%) | 170,417 (97.43 %) | 45,390 (93.75 %) |
| Yes | 7,517 (3.37%) | 4,489 (2.57 %) | 3,028 (6.25 %) |
| **Dementia** | | | |
| No | 216,525 (96.96%) | 169,637 (96.99 %) | 46,888 (96.84 %) |
| Yes | 6,799 (3.04%) | 5,269 (3.01 %) | 1,530 (3.16 %) |
| **Thyroidism** | | | |
| No | 215,894 (96.67%) | 173,581 (99.24 %) | 42,313 (87.39 %) |
| Yes | 7,430 (3.33%) | 1,325 (0.76 %) | 6,105 (12.61 %) |
| **Anaemia** | | | |
| No | 199,438 (89.30%) | 151,549 (86.65 %) | 47,889 (98.91 %) |
| Yes | 23,886 (10.70%) | 23,357 (13.35 %) | 529 (1.09 %) |
| **Osteoporosis** | | | |
| No | 205,345 (91.95%) | 161,061 (92.08 %) | 44,284 (91.46 %) |
| Yes | 17,979 (8.05%) | 13,845 (7.92 %) | 4,134 (8.54 %) |
| **PJI before in same joint** | | | |
| No | 222,353 (99.57%) | 174,100 (99.54 %) | 48,253 (99.66 %) |
| Yes | 971 (0.43%) | 806 (0.46 %) | 165 (0.34 %) |
| **PJI before in another joint (* not only hip or knee)** | | | |
| No | 220,838 (98.89%) | 173,074 (98.95 %) | 47,764 (98.65 %) |
| Yes | 2,486 (1.11%) | 1,832 (1.05 %) | 654 (1.35 %) |

For peer review only

**Table S 7.** Treatment history of patients at the time of hip or knee implant surgery.

| Variable | All | AURUM | GOLD |
|---|---|---|---|
| **Steroids injection** | | | |
| No use | 206,245 (92.35%) | 164,047 (93.79 %) | 42,198 (87.15 %) |
| Use < 3 months | 1,256 (0.56%) | 810 (0.46 %) | 446 (0.92 %) |
| Use 3 - 6 months | 2,276 (1.02%) | 1,456 (0.83 %) | 820 (1.69 %) |
| Use > 6 months | 13,547 (6.07%) | 8,593 (4.91 %) | 4,954 (10.23 %) |
| **Chondroitin glucosamine** | | | |
| No use | 219,306 (98.20%) | 173,018 (98.92 %) | 46,288 (95.60 %) |
| Use < 3 months | 930 (0.42%) | 482 (0.28 %) | 448 (0.93 %) |
| Use 3 - 6 months | 259 (0.12%) | 134 (0.08 %) | 125 (0.26 %) |
| Use > 6 months | 2,829 (1.27%) | 1,272 (0.73 %) | 1,557 (3.22 %) |
| **NSAID** | | | |
| No use | 97,243 (43.54%) | 89,492 (51.17 %) | 7,751 (16.01 %) |
| Use < 3 months | 48,354 (21.65%) | 37,077 (21.20 %) | 11,277 (23.29 %) |
| Use 3 - 6 months | 13,169 (5.90%) | 9,851 (5.63 %) | 3,318 (6.85 %) |
| Use > 6 months | 64,558 (28.91%) | 38,486 (22.00 %) | 26,072 (53.85 %) |
| **Methotrexate** | | | |
| No use | 218,503 (97.84%) | 171,414 (98.00 %) | 47,089 (97.26 %) |
| Use < 3 months | 3,255 (1.46%) | 2,559 (1.46 %) | 696 (1.44 %) |
| Use 3 - 6 months | 174 (0.08%) | 124 (0.07 %) | 50 (0.10 %) |
| Use > 6 months | 1,392 (0.62%) | 809 (0.46 %) | 583 (1.20 %) |
| **DMRAD** | | | |
| No use | 219,948 (98.49%) | 172,997 (98.91 %) | 46,951 (96.97 %) |
| Use < 3 months | 1,626 (0.73%) | 1,188 (0.68 %) | 438 (0.90 %) |
| Use 3 - 6 months | 155 (0.07%) | 112 (0.06 %) | 43 (0.09 %) |
| Use > 6 months | 1,595 (0.71%) | 609 (0.35 %) | 986 (2.04 %) |
| **Antibacterial** | | | |
| No use | 91,794 (41.10%) | 68,716 (39.29 %) | 23,078 (47.66 %) |
| Use < 3 months | 35,730 (16.00%) | 34,370 (19.65 %) | 1,360 (2.81 %) |
| Use 3 - 6 months | 15,961 (7.15%) | 14,903 (8.52 %) | 1,058 (2.19 %) |
| Use > 6 months | 79,839 (35.75%) | 56,917 (32.54 %) | 22,922 (47.34 %) |
| **Antifungal** | | | |
| No use | 192,800 (86.33%) | 148,117 (84.68 %) | 44,683 (92.29 %) |
| Use < 3 months | 4,900 (2.19%) | 4,747 (2.71 %) | 153 (0.32 %) |
| Use 3 - 6 months | 2,842 (1.27%) | 2,721 (1.56 %) | 121 (0.25 %) |
| Use > 6 months | 22,782 (10.20%) | 19,321 (11.05 %) | 3,461 (7.15 %) |
| **Direct Oral Anticoagulant** | | | |
| No use | 222,761 (99.75%) | 174,417 (99.72 %) | 48,344 (99.85 %) |
| Use < 3 months | 324 (0.15%) | 288 (0.16 %) | 36 (0.07 %) |
| Use 3 - 6 months | 54 (0.02%) | 51 (0.03 %) | 3 (0.01 %) |
| Use > 6 months | 185 (0.08%) | 150 (0.09 %) | 35 (0.07 %) |
| **Heparin** | | | |
| No use | 221,208 (99.05%) | 173,345 (99.11 %) | 47,863 (98.85 %) |

For peer review only

| | | | |
|---|---|---|---|
| Use < 3 months | 334 (0.15%) | 317 (0.18 %) | 17 (0.04 %) |
| Use 3 - 6 months | 163 (0.07%) | 142 (0.08 %) | 21 (0.04 %) |
| Use > 6 months | 1,619 (0.72%) | 1,102 (0.63 %) | 517 (1.07 %) |
| **Warfarin** | | | |
| No use | 208,608 (93.41%) | 164,436 (94.01 %) | 44,172 (91.23 %) |
| Use < 3 months | 9,494 (4.25%) | 7,499 (4.29 %) | 1,995 (4.12 %) |
| Use 3 - 6 months | 879 (0.39%) | 695 (0.40 %) | 184 (0.38 %) |
| Use > 6 months | 4,343 (1.94%) | 2,276 (1.30 %) | 2,067 (4.27 %) |
| **Intra-articular injections (n)** | | | |
| 0 | 215,281 (96.40%) | 168,424 (96.29 %) | 46,857 (96.78 %) |
| 1 | 5,856 (2.62%) | 4,709 (2.69 %) | 1,147 (2.37 %) |
| 2 | 1,320 (0.59%) | 1,063 (0.61 %) | 257 (0.53 %) |
| 3 | 444 (0.20%) | 367 (0.21 %) | 77 (0.16 %) |
| 4 | 164 (0.07%) | 130 (0.07 %) | 34 (0.07 %) |
| 5 | 117 (0.05%) | 96 (0.05 %) | 21 (0.04 %) |
| > 5 | 142 (0.06%) | 117 (0.07 %) | 25 (0.05 %) |
| **Last intra-articular injection** | | | |
| No use | 215,281 (96.40%) | 168,424 (96.29 %) | 46,857 (96.78 %) |
| Use < 3 months | 341 (0.15%) | 279 (0.16 %) | 62 (0.13 %) |
| Use 3 - 6 months | 1,309 (0.59%) | 1,036 (0.59 %) | 273 (0.56 %) |
| Use > 6 months | 6,393 (2.86%) | 5,167 (2.95 %) | 1,226 (2.53 %) |
| **Intra-articular injection in other joints (n)** | | | |
| 0 | 200,901 (89.96%) | 156,820 (89.66 %) | 44,081 (91.04 %) |
| 1 | 258 (0.12%) | 200 (0.11 %) | 58 (0.12 %) |
| 2 | 3,014 (1.35%) | 2,401 (1.37 %) | 613 (1.27 %) |
| 3 | 6,579 (2.95%) | 5,270 (3.01 %) | 1,309 (2.70 %) |
| 4 | 4,315 (1.93%) | 3,438 (1.97 %) | 877 (1.81 %) |
| 5 | 1,407 (0.63%) | 1,130 (0.65 %) | 277 (0.57 %) |
| > 5 | 6,850 (3.07%) | 5,647 (3.23 %) | 1,203 (2.48 %) |
| **Last intra-articular injection in other joints** | | | |
| No use | 200,901 (89.96%) | 156,820 (89.66 %) | 44,081 (91.04 %) |
| Use < 3 months | 974 (0.44%) | 777 (0.44 %) | 197 (0.41 %) |
| Use 3 - 6 months | 2,885 (1.29%) | 2,305 (1.32 %) | 580 (1.20 %) |
| Use > 6 months | 18,564 (8.31%) | 15,004 (8.58 %) | 3,560 (7.35 %) |

For peer review only

**Table S 8.** Summary of fitting performance measuremement for fitting results from CPRD AURUM database with different parametric models.

| Parametric model | AIC | BIC | Loglik |
|---|---|---|---|
| Exponential | 26671.5 | 27749.2 | -13730.0 |
| Gaussian | 28030.7 | 29118.5 | -15416.5 |
| Logistic | 28097.6 | 29185.4 | -14439.8 |
| Log-logistic | 26190.8 | 27278.6 | -13479.9 |
| Lognormal | 26187.4 | 27275.2 | -13459.8 |
| Weibull | 26192.6 | 27280.4 | -13480.2 |

**Table S 9**. Summary of fitting performance measuremement for fitting results from CPRD GOLD database with different parametric models.

| Parametric model | AIC | BIC | Loglik |
|---|---|---|---|
| Exponential | 7674.76 | 8573.56 | -3927.22 |
| Gaussian | 8097.86 | 9005.83 | -4134.94 |
| Logistic | 8116.71 | 9024.69 | -4146.93 |
| Log-logistic | 7543.99 | 8451.96 | -3857.65 |
| Lognormal | 7463.78 | 7793.95 | -3852.10 |
| Weibull | 7544.66 | 8452.64 | -3857.71 |

For peer review only

**Table S 10.** Regression coefficients and standard error (SE) for log-normal risk equation of time to PJI derived from CPRD AURUM and CPRD GOLD.

| Variable | AURUM | GOLD | SE AURUM | SE GOLD | *P* value |
|---|---|---|---|---|---|
| **Scale** | 5.194 | 4.759 | 1.404 | 1.650 | 0.841 |
| **Intercept** | 19.470 | 20.618 | 0.132 | 0.264 | < 0.001 |
| **Male** | -0.813 | -0.952 | 0.408 | 0.967 | 0.895 |
| **Age** | | | | | |
| ≤ 45 | Reference | | | | |
| 46 – 55 | 1.247 | 0.134 | 0.378 | 0.931 | 0.268 |
| 56 – 65 | 1.611 | 0.843 | 0.379 | 0.939 | 0.448 |
| 66 – 75 | 1.968 | 1.241 | 0.389 | 0.958 | 0.482 |
| 76 – 85 | 2.044 | 1.299 | 0.498 | 1.274 | 0.586 |
| > 85 | 3.390 | 3.329 | 0.550 | 0.988 | 0.957 |
| **BMI** | | | | | |
| ≤ 20 | Reference | | | | |
| 20 - 25 | -0.271 | -0.946 | 0.535 | 0.983 | 0.546 |
| 25 - 30 | -0.347 | -0.493 | 0.54 | 0.992 | 0.897 |
| 30 - 35 | -0.715 | -0.934 | 0.547 | 1.012 | 0.849 |
| 35 - 50 | -1.186 | -1.271 | 0.979 | 2.093 | 0.971 |
| >50 | -1.628 | -2.693 | 0.526 | 1.132 | 0.394 |
| Unknown | -1.116 | -0.653 | 1.955 | 0.432 | 0.817 |
| **Smoking status** | | | | | |
| Non-smoker | Reference | | | | |
| Cigar smoker | 0.249 | NA | 0.284 | NA | NA |
| Current smoker | -0.181 | -0.091 | 0.137 | 0.265 | 0.763 |
| Ex-smoker | -0.194 | 0.157 | 1.359 | 2.274 | 0.895 |
| Heavy smoker | -0.623 | -1.003 | 1.112 | 1.361 | 0.829 |
| Light smoker | 0.609 | -1.455 | 1.140 | 0.001 | 0.070 |
| Moderate smoker | 0.127 | NA | 1.004 | NA | NA |
| Quitting | 0.469 | NA | 0.22 | NA | NA |
| Unknown | -0.083 | 0.074 | 1.391 | 1.854 | 0.946 |
| Passive smoker | NA | NA | NA | 1.483 | NA |
| **Alcohol consumption** | | | | | |
| Non-drinker | Reference | | | | |
| Ex drinker | 0.866 | NA | 1.199 | 0.465 | NA |
| Heavy drinker | 0.828 | -0.218 | 1.231 | 0.759 | 0.47 |
| Light drinker | 0.966 | 0.761 | 1.302 | 0.973 | 0.901 |
| Moderate drinker | 1.706 | NA | 1.36 | NA | NA |
| Other | 0.825 | 0.805 | 1.267 | 0.405 | 0.988 |
| Social drinker | 0.783 | 0.962 | 1.194 | 0.002 | 0.881 |
| Unknown | 1.098 | -0.118 | 1.547 | 0.241 | 0.437 |
| Very heavy drinker | 0.091 | NA | 0.124 | 1.334 | NA |
| **Right side of operation** | 0.060 | 0.474 | 0.285 | 0.544 | 0.504 |
| **Type of fixation** | | | | | |

For peer review only

| | | | | | |
|---|---|---|---|---|---|
| Cemented | Reference | | | | |
| Hybrid | 0.155 | -0.801 | 0.164 | 0.369 | 0.018 |
| Non cemented | 0.123 | 0.106 | 0.269 | 0.51 | 0.976 |
| Unknown | 0.048 | -0.024 | 0.181 | 0.365 | 0.860 |
| **Primary arthroplasty** | | | | | |
| Yes | Reference | | | | |
| No | -2.655 | -1.927 | 0.656 | 1.953 | 0.724 |
| Unknown | -2.674 | -0.622 | 0.211 | 0.451 | < 0.001 |
| **Admission type** | | | | | |
| Elective | Reference | | | | |
| A&E | -1.013 | -1.256 | 1.157 | 1.767 | 0.908 |
| Unknown | 0.618 | -0.630 | 0.490 | 0.763 | 0.169 |
| **Patella resurfacing** | | | | | |
| No | NA | NA | NA | NA | NA |
| Yes | 0.736 | -0.862 | 0.151 | 0.326 | < 0.001 |
| N/A | 0.437 | 1.463 | 0.635 | 81880.48 | 1 |
| **Graft** | | | | | |
| No | Reference | | | | |
| Autograft | -0.048 | 26.046 | 2.238 | 2.630 | < 0.001 |
| Autograft + other | -0.458 | -6.485 | 0.486 | 1.256 | < 0.001 |
| Other graft | 0.152 | 0.597 | 0.263 | 0.525 | 0.449 |
| **Replacement year** | | | | | |
| 2007 | Reference | | | | |
| 2008 | 0.387 | 0.428 | 0.283 | 0.482 | 0.942 |
| 2009 | 0.506 | 0.031 | 0.284 | 0.467 | 0.385 |
| 2010 | 0.378 | -0.238 | 0.288 | 0.489 | 0.278 |
| 2011 | 0.35 | 0.065 | 0.294 | 0.493 | 0.62 |
| 2012 | 0.521 | -0.062 | 0.302 | 0.472 | 0.298 |
| 2013 | 0.637 | -0.674 | 0.305 | 0.487 | 0.023 |
| 2014 | 0.635 | -0.383 | 0.028 | 0.058 | < 0.001 |
| **Atrial Fibrillation** | -0.259 | -0.784 | 0.182 | 1.128 | 0.646 |
| **CKD** | -0.094 | 0.813 | 1.227 | 0 | 0.46 |
| **Liver failure** | 0.299 | NA | 0.394 | 0.751 | NA |
| **Pulmonary Embolism** | -0.071 | -0.517 | 0.276 | 0.511 | 0.443 |
| **Deep Vein Thrombosis** | -0.531 | 0.362 | 0.168 | 0.321 | 0.014 |
| **Diabetes** | -0.468 | 0.205 | 0.145 | 0.272 | 0.029 |
| **Osteoarthritis** | -0.249 | -0.199 | 0.143 | 0.506 | 0.924 |
| **Rheumatoid Arthritis** | -0.005 | -0.274 | 0.136 | 0.287 | 0.397 |
| **Active Cancer** | 0.005 | -0.159 | 0.425 | 1.034 | 0.883 |
| **Heart Failure** | 0.071 | 0.651 | 0.322 | 0.691 | 0.447 |
| **Myocardial Infarction** | -0.616 | 0.717 | 0.312 | 0.464 | 0.017 |
| **Ischaemic Heart Disease** | 0.286 | -0.130 | 0.130 | 0.255 | 0.146 |

For peer review only

| | | | | | |
|---|---|---|---|---|---|
| **Hypertension** | -0.127 | -0.254 | 0.256 | 0.496 | 0.820 |
| **COPD** | 0.298 | -0.213 | 0.639 | 1.655 | 0.773 |
| **Haemorrhagic Stroke** | 0.616 | -0.263 | 0.549 | 0.539 | 0.253 |
| **Ischaemic Stroke** | 0.094 | 0.022 | 0.563 | 0.975 | 0.949 |
| **Dementia** | 0.473 | -0.466 | 0.701 | 0.369 | 0.235 |
| **Thyroidism** | -0.309 | -0.197 | 0.182 | 0 | 0.538 |
| **Anaemia** | -0.026 | NA | 0.260 | 0.557 | NA |
| **Osteoporosis** | 0.135 | 0.648 | 0.397 | 0.895 | 0.600 |
| **PJI before in same joint** | -2.493 | -0.872 | 0.333 | 0.596 | 0.018 |
| **PJI before in another joint** | -1.726 | -2.178 | 0.766 | 1.204 | 0.751 |
| **Steroids injection** | | | | | |
| No use | Reference | | | | |
| Use ≤ 3 months | -0.629 | 0.165 | 0.543 | 0.979 | 0.478 |
| Use 3 - 6 months | -0.962 | 0.777 | 0.258 | 0.364 | < 0.001 |
| Use > 6 months | -0.383 | -0.079 | 1.368 | 1.607 | 0.885 |
| **Chondroitin Glucosamine** | | | | | |
| No use | Reference | | | | |
| Use ≤ 3 months | 0.920 | 1.314 | 1.841 | 0 | 0.831 |
| Use 3 - 6 months | -0.791 | NA | 0.638 | 0.692 | NA |
| Use > 6 months | -0.334 | 0.622 | 0.162 | 0.474 | 0.056 |
| **NSAID** | | | | | |
| No use | Reference | | | | |
| Use ≤ 3 months | -0.678 | -1.552 | 0.257 | 0.568 | 0.161 |
| Use 3 - 6 months | -0.472 | -1.482 | 0.177 | 0.448 | 0.036 |
| Use > 6 months | 0.136 | -0.459 | 0.440 | 0.980 | 0.580 |
| **Methotrexate** | | | | | |
| No use | Reference | | | | |
| Use ≤ 3 months | -0.787 | 0.358 | 0 | 0 | < 0.001 |
| Use 3 - 6 months | NA | NA | 0.771 | 0.936 | NA |
| Use > 6 months | -0.668 | -0.329 | 0.897 | 0.87 | 0.786 |
| **DMRAD** | | | | | |
| No use | Reference | | | | |
| Use ≤ 3 months | 1.494 | -2.089 | 0 | 0 | < 0.001 |
| Use 3 - 6 months | NA | NA | 0.875 | 0.826 | NA |
| Use > 6 months | -0.434 | 0.023 | 0.19 | 0.767 | 0.563 |
| **Antibacterial** | | | | | |
| No use | Reference | | | | |
| Use ≤ 3 months | -0.394 | 0.376 | 0.233 | 0.686 | 0.288 |
| Use 3 - 6 months | -0.568 | -0.889 | 0.174 | 0.249 | 0.291 |
| Use > 6 months | -0.362 | -0.136 | 0.324 | 0 | 0.485 |
| **Antifungal** | | | | | |
| No use | Reference | | | | |
| Use ≤ 3 months | -0.728 | NA | 0.467 | 0 | NA |

For peer review only

| | | | | | |
|---|---|---|---|---|---|
| Use 3 - 6 months | -0.115 | NA | 0.186 | 0.364 | NA |
| Use > 6 months | -0.434 | -1.086 | 1.112 | 2.132 | 0.786 |
| **Direct Oral Anticoagulant** | | | | | |
| No use | Reference | | | | |
| Use ≤ 3 months | -1.508 | -3.789 | 0 | 0 | < 0.001 |
| Use 3 - 6 months | NA | NA | 2.132 | 0 | NA |
| Use > 6 months | 0.694 | NA | 1.081 | 0 | NA |
| **Heparin** | | | | | |
| No use | Reference | | | | |
| Use ≤ 3 months | -0.878 | NA | 1.239 | 0 | NA |
| Use 3 - 6 months | -2.067 | NA | 0.732 | 1.011 | NA |
| Use > 6 months | 0.162 | -0.484 | 0.350 | 0.661 | 0.388 |
| **Warfarin** | | | | | |
| No use | Reference | | | | |
| Use ≤ 3 months | -0.021 | -0.914 | 0.832 | 1.476 | 0.598 |
| Use 3 - 6 months | -0.433 | -1.07 | 0.531 | 0.640 | 0.444 |
| Use > 6 months | 0.273 | -0.053 | 1.714 | 3527.096 | 1 |
| **Intra-articular injection** | | | | | |
| No use | Reference | | | | |
| Use ≤ 3 months | 0.308 | 19.912 | 1.647 | 0 | < 0.001 |
| Use 3 - 6 months | -4.414 | NA | 0.396 | 0.915 | NA |
| Use > 6 months | -0.171 | -0.238 | 0.973 | 1.973 | 0.976 |
| **Intra-articular injections in other joints** | | | | | |
| No use | Reference | | | | |
| Use ≤ 3 months | -0.358 | -0.131 | 1.519 | 1.222 | 0.907 |
| Use 3 - 6 months | 3.417 | -0.589 | 0.239 | 0.529 | < 0.001 |
| Use > 6 months | -0.144 | 0.349 | 0.270 | 0.498 | 0.384 |

For peer review only

**Table S 11.** C- statistic values for pooled risk equation at different follow-up.

| Follow-up | Mean | 95% CI |
|---|---|---|
| 1 year | 0.733 | 0.712 - 0.756 |
| 2 years | 0.727 | 0.708 - 0.744 |
| 3 years | 0.725 | 0.708 - 0.743 |
| 4 years | 0.723 | 0.708 - 0.738 |
| 5 years | 0.721 | 0.706 - 0.736 |

For peer review only

# TRIPOD Checklist: Prediction Model Development and Validation

| Section/Topic | Item | | Checklist Item | Page |
|---|---|---|---|---|
| **Title and abstract** | | | | |
| Title | 1 | D;V | Identify the study as developing and/or validating a multivariable prediction model, the target population, and the outcome to be predicted. | 1 |
| Abstract | 2 | D;V | Provide a summary of objectives, study design, setting, participants, sample size, predictors, outcome, statistical analysis, results, and conclusions. | 2 |
| **Introduction** | | | | |
| Background and objectives | 3a | D;V | Explain the medical context (including whether diagnostic or prognostic) and rationale for developing or validating the multivariable prediction model, including references to existing models. | 4 |
| | 3b | D;V | Specify the objectives, including whether the study describes the development or validation of the model or both. | 4-5 |
| **Methods** | | | | |
| Source of data | 4a | D;V | Describe the study design or source of data (e.g., randomized trial, cohort, or registry data), separately for the development and validation data sets, if applicable. | 5 |
| | 4b | D;V | Specify the key study dates, including start of accrual; end of accrual; and, if applicable, end of follow-up. | 6 |
| Participants | 5a | D;V | Specify key elements of the study setting (e.g., primary care, secondary care, general population) including number and location of centres. | 5-6 |
| | 5b | D;V | Describe eligibility criteria for participants. | 6 |
| | 5c | D;V | Give details of treatments received, if relevant. | 6 |
| Outcome | 6a | D;V | Clearly define the outcome that is predicted by the prediction model, including how and when assessed. | 7 |
| | 6b | D;V | Report any actions to blind assessment of the outcome to be predicted. | NA |
| Predictors | 7a | D;V | Clearly define all predictors used in developing or validating the multivariable prediction model, including how and when they were measured. | 6-7 |
| | 7b | D;V | Report any actions to blind assessment of predictors for the outcome and other predictors. | NA |
| Sample size | 8 | D;V | Explain how the study size was arrived at. | 9 |
| Missing data | 9 | D;V | Describe how missing data were handled (e.g., complete-case analysis, single imputation, multiple imputation) with details of any imputation method. | 7 |
| Statistical analysis methods | 10a | D | Describe how predictors were handled in the analyses. | 8 |
| | 10b | D | Specify type of model, all model-building procedures (including any predictor selection), and method for internal validation. | 7-8 |
| | 10c | V | For validation, describe how the predictions were calculated. | 7-8 |
| | 10d | D;V | Specify all measures used to assess model performance and, if relevant, to compare multiple models. | 8 |
| | 10e | V | Describe any model updating (e.g., recalibration) arising from the validation, if done. | NA |
| Risk groups | 11 | D;V | Provide details on how risk groups were created, if done. | NA |
| Development vs. validation | 12 | V | For validation, identify any differences from the development data in setting, eligibility criteria, outcome, and predictors. | 8 |
| **Results** | | | | |
| Participants | 13a | D;V | Describe the flow of participants through the study, including the number of participants with and without the outcome and, if applicable, a summary of the follow-up time. A diagram may be helpful. | 9 |
| | 13b | D;V | Describe the characteristics of the participants (basic demographics, clinical features, available predictors), including the number of participants with missing data for predictors and outcome. | 9-10 |
| | 13c | V | For validation, show a comparison with the development data of the distribution of important variables (demographics, predictors and outcome). | NA |
| Model development | 14a | D | Specify the number of participants and outcome events in each analysis. | 10 |
| | 14b | D | If done, report the unadjusted association between each candidate predictor and outcome. | NA |
| Model specification | 15a | D | Present the full prediction model to allow predictions for individuals (i.e., all regression coefficients, and model intercept or baseline survival at a given time point). | Table 1 |
| | 15b | D | Explain how to the use the prediction model. | 9-10 |
| Model performance | 16 | D;V | Report performance measures (with CIs) for the prediction model. | 10-11 |
| Model-updating | 17 | V | If done, report the results from any model updating (i.e., model specification, model performance). | NA |
| **Discussion** | | | | |
| Limitations | 18 | D;V | Discuss any limitations of the study (such as nonrepresentative sample, few events per predictor, missing data). | 13 |
| Interpretation | 19a | V | For validation, discuss the results with reference to performance in the development data, and any other validation data. | 12 |
| | 19b | D;V | Give an overall interpretation of the results, considering objectives, limitations, results from similar studies, and other relevant evidence. | 11-13 |
| Implications | 20 | D;V | Discuss the potential clinical use of the model and implications for future research. | 11-14 |
| **Other information** | | | | |
| Supplementary information | 21 | D;V | Provide information about the availability of supplementary resources, such as study protocol, Web calculator, and data sets. | 14 |
| Funding | 22 | D;V | Give the source of funding and the role of the funders for the present study. | 14 |

*Items relevant only to the development of a prediction model are denoted by D, items relating solely to a validation of a prediction model are denoted by V, and items relating to both are denoted D;V. We recommend using the TRIPOD Checklist in conjunction with the TRIPOD Explanation and Elaboration document.
